# Elicitation of liver-stage immunity by nanoparticle immunogens displaying *P. falciparum* CSP-derived antigens

**DOI:** 10.1038/s41541-025-01140-x

**Published:** 2025-05-05

**Authors:** Mark D. Langowski, Joseph R. Francica, Alex L. Roederer, Nicholas K. Hurlburt, Justas V. Rodarte, Lais Da Silva Pereira, Barbara J. Flynn, Brian Bonilla, Marlon Dillon, Patience Kiyuka, Rashmi Ravichandran, Connor Weidle, Lauren Carter, Mangala Rao, Gary R. Matyas, Marion Pepper, Azza H. Idris, Robert A. Seder, Marie Pancera, Neil P. King

**Affiliations:** 1 Institute for Protein Design, University of Washington, Seattle, WA USA; 2 Department of Biochemistry, University of Washington, Seattle, WA USA; 3 Graduate Program in Molecular and Cellular Biology, University of Washington, Seattle, WA USA; 4 Vaccine Research Center, National Institute of Allergy and Infectious Diseases, National Institutes of Health, Bethesda, MD USA; 5 Vaccine and Infectious Disease Division, Fred Hutchinson Cancer Center, Seattle, WA USA; 6 Henry M. Jackson Foundation for the Advancement of Military Medicine, Bethesda, MD USA; 7 U.S. Military HIV Research Program, Center for Infectious Diseases Research, Walter Reed Army Institute of Research, Silver Spring, MD USA; 8 Department of Immunology, University of Washington, Seattle, WA USA

**Keywords:** Vaccines, Malaria, Protein vaccines, Recombinant vaccine

## Abstract

A vaccine that provides robust, durable protection against malaria remains a global health priority. Although a breakthrough in the fight against malaria has recently been achieved by the licensure of two vaccines based on the circumsporozoite protein (CSP), the effectiveness and durability of protection can still be improved. Both vaccines contain a portion of CSP that does not include epitopes targeted by recently identified, potently protective monoclonal antibodies, suggesting that newer immunogens can expand the breadth of immunity and potentially increase protection. Here we explored >100 alternative CSP-based immunogens and evaluated the immunogenicity and protection of a large number of candidates, comparing several to the licensed R21 vaccine. The data highlight several general features that improve the stability and immunogenicity of CSP-based vaccines, such as inclusion of the C-terminal domain and high-density display on protein nanoparticle scaffolds. We also identify antigen design strategies that do not warrant further exploration, such as synthetic repeat regions that include non-native repeat cadences. The benchmark R21 vaccine outperformed our best immunogen for immunogenicity and protection. Overall, our data provide valuable insights on the inclusion of junctional region epitopes that will guide the development of potent and durable vaccines against malaria.

## Introduction

According to the WHO malaria report, malaria caused an estimated 249 million cases and 608,000 deaths in 2022^1^. The majority of these deaths were caused by *Plasmodium falciparum* (Pf), primarily in children under 5 years of age in Africa. Though control efforts have reduced these numbers over the past two decades, disruptions from the COVID-19 pandemic and growing artemisinin and insecticide resistance threaten advances in treatment and vector control^1^. The substantial morbidity, mortality, and economic impact of malaria motivate the development of immune interventions such as highly effective vaccines or protective antibodies to prevent and ultimately eliminate malaria worldwide.

Although malaria vaccine development is complicated by the complex, multi-stage life cycle of *Plasmodium*, the potential of preventing infection has led many vaccine efforts to focus on the pre-erythrocytic stage. These vaccines aim to prevent the relatively small number of sporozoites that enter the dermis or blood from reaching the liver, thereby providing sterile protection^2^. However, because sporozoites access the liver within 1-3 hours and a single parasite can initiate liver infection, an effective vaccine must elicit high and durable levels of protective antibodies^3^. The circumsporozoite protein (CSP) is the most abundantly expressed protein on the surface of sporozoites^4^ and is the antigenic component of the RTS,S/AS01 and R21/Matrix-M vaccines, which showed 55.8% and 68% efficacy in standard sites at 1 year respectively in clinical trials^5,6^ and are approved by European regulators and African countries, and recommended by the WHO^7,8^. Although there is no structure of full-length PfCSP and its function remains unclear, the protein comprises three distinct regions. The N-terminal domain (NTD) includes a signal peptide, a free cysteine, a pair of *Plasmodium* Export Element (PEXEL) sites and protease cleavage sites that are suspected to be necessary for hepatocyte invasion^9–11^. The central part of the protein consists of a series of tetrapeptide repeats, the exact number and sequence of which vary across *P. falciparum* strains^12^. In the prototypic strain 3D7, the central repeat region begins with a “junctional region” which consists of a single “junctional” NPDP motif, followed by three “major” NANP and “minor” NVDP tandem repeats. The rest of the repeat region consists of 35 copies of the NANP repeat with a single NVDP repeat at its center. Finally, the C-terminal domain (CTD), for which a crystal structure is available, adopts a thrombospondin-like fold followed by a GPI anchor sequence^13^. RTS,S and R21 both contain a truncated portion of PfCSP comprising 18.5 NANP repeats and the CTD, which may limit antibody responses to other epitopes that could contribute to protection^14,15^. Indeed, until recently, the majority of known anti-CSP protective antibodies targeted the central NANP repeat region. However, human antibodies against novel sites of vulnerability not present in RTS,S/R21—including the junctional and minor epitopes—have now been identified^14–17^. Passive immunization of adults with one such antibody targeting the junctional epitope, CIS43LS, led to a 75% (10 mg/kg dose) and 88% (40 mg/kg dose) reduction in infection over 6 months in a recent Phase II clinical trial^18^, and passive immunization of children with minor repeat targeting L9LS showed up to 77% efficacy against clinical malaria in a Phase II clinical trial^19^. These results establish that junctional and minor repeat epitope sites on PfCSP are indeed neutralizing sites to be used for the design and evaluation of vaccines beyond those present in RTS,S and R21. Such vaccines may increase the breadth and potency of the immune response to potentially overcome the waning immune responses observed with RTS,S/AS01 and R21/Matrix-M^3,20^.

Structural biology is a powerful tool that informs vaccine development by visualizing antigen in complex with protective (and non-protective) antibodies^21^. The two most successful examples of structure-based vaccine design to date, both of which led to licensed vaccines, targeted RSV^22,23^ and SARS-CoV-2^24–26^. In both cases, structures of the viral fusion glycoprotein (or those of other betacoronaviruses in the case of SARS-CoV-2^27,28^) in its native-like prefusion conformation enabled the design of prefusion-stabilized antigens that elicit potent neutralizing antibody responses^28,29^. By contrast, the lack of detailed information on the structure of the full-length PfCSP on the surface of the parasite has hindered structure-based vaccine design. However, over the last several years there have been major advances in the structural information on protective monoclonal antibodies (mAbs) targeting the major repeats, minor repeats, and junctional region of PfCSP^14–16,30–37^. These structures, which mainly comprise mAbs bound to PfCSP-derived peptides or truncated proteins, and other biophysical analyses, have revealed a structural heterogeneity in the antigens that suggests the repeat region of PfCSP is relatively disordered and can adopt various conformations^38^. Although designed antigens based on these structures have not yet been reported, they have led to the exploration of immunogens that include additional regions of CSP beyond the major repeats and CTD. Most of these have comprised peptide antigens based on the junctional region^39–44^, although in one case a peptide antigen fused to a major repeat-targeting Fab was displayed on a self-assembling nanoparticle to favor the acquisition of homotypic Fab-Fab contacts during affinity maturation^45^, a phenomenon observed in some major repeat-targeting antibodies and the minor repeat-targeting antibody L9^36,46^.

Multivalent display of antigens on self-assembling or particulate scaffolds has been shown to improve vaccine-elicited antibody responses by enhancing vaccine trafficking, antigen presentation, and B cell activation^47–50^. Multivalent antigen display has been extensively explored as an approach to malaria vaccine design, and in fact RTS,S was until recently the only licensed vaccine in which an antigen is displayed on a heterologous self-assembling scaffold, in this case Hepatitis B virus surface antigen virus-like particles^51^. Beyond RTS,S and R21, numerous groups have used virus-like particles, ferritin, lumazine synthase, or self-assembling peptides to improve the potency of CSP-derived antigens in preclinical studies^39–41,45,52–57^. Computationally designed self-assembling protein nanoparticles have recently emerged as a robust and versatile platform for multivalent antigen display that enables many structural and antigenic characteristics of the immunogen to be precisely varied^58–63^. Furthermore, a computationally designed two-component nanoparticle vaccine for SARS-CoV-2, SKYCovione™, recently became the second licensed vaccine—after RTS,S—in which an antigen is displayed on a heterologous self-assembling protein scaffold, establishing the clinical and commercial viability of the platform^62,64^.

Here we combined extensive exploration of different PfCSP-based antigens with multivalent display on self-assembling protein scaffolds, including computationally designed protein nanoparticles, and benchmark them against the state-of-the-art vaccine R21. We found that the epitope specificity of vaccine-elicited antibodies could be tuned by displaying antigens comprising different target epitopes in PfCSP, and that the most potently protective responses were elicited by nanoparticle immunogens primarily displaying the major repeats.

## Results

### Design, immunogenicity, and protective efficacy of stabilized PfCSP variants displayed on nanoparticles

**Fig. 1:**
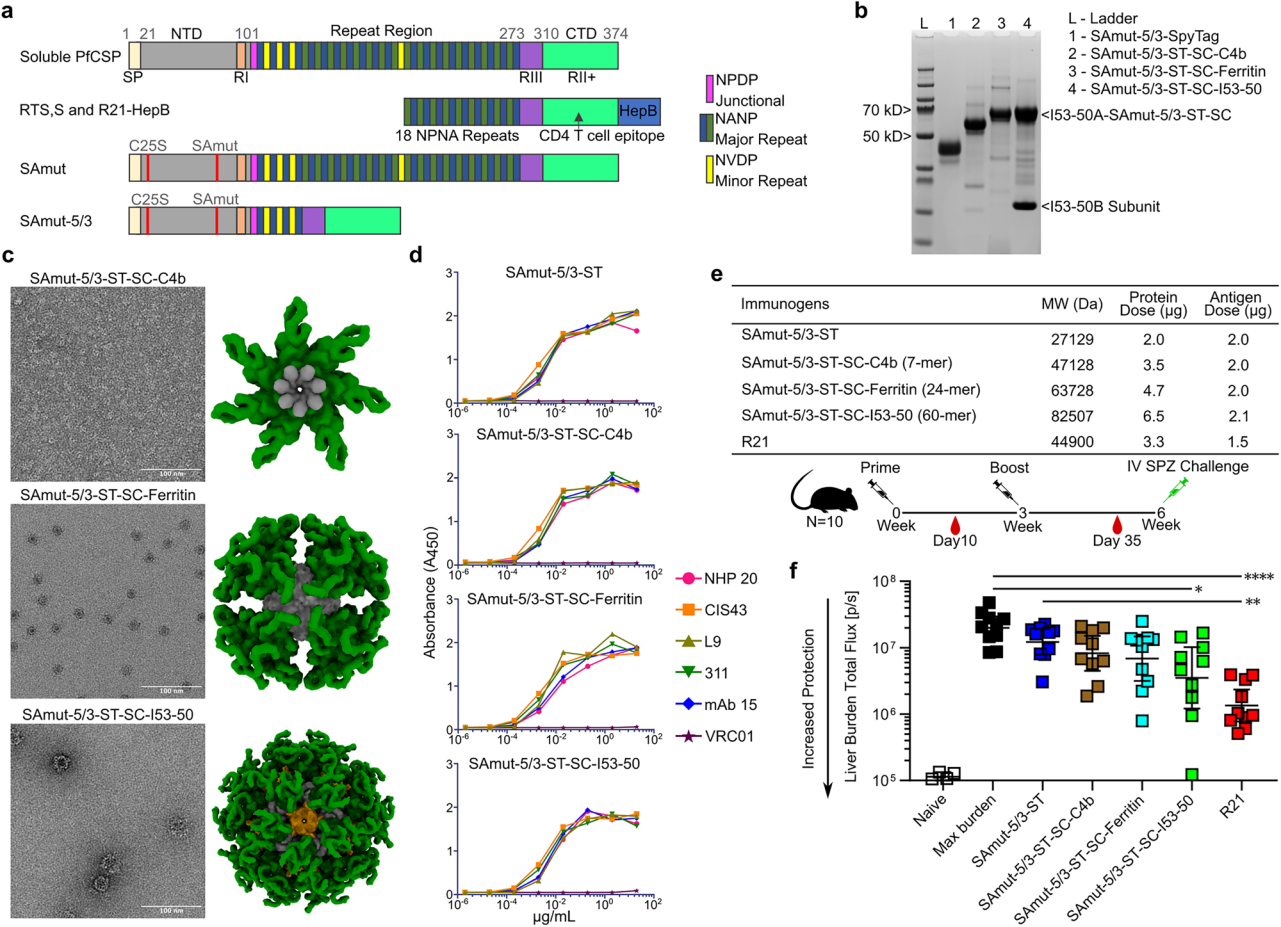
Design, biophysical characterization, antigenicity, and immunogenicity of SAmut-5/3 multimers. **a** Primary structures of PfCSP variants, including the R21 immunogen. SP, signal peptide; RI, Region I; RIII, Region III; RII + , Region II; GPI, glycosylphosphatidylinositol anchor sequence. Red lines indicate sites of cysteine and PEXEL mutations. **b** Reducing SDS-PAGE of purified SAmut-5/3 immunogens. **c** nsEM of the SAmut-5/3 multimers. Micrographs and structural models of each multimer are shown. **d** Binding of multimers to PfCSP-directed mAbs measured by ELISA. NHP20 is an unpublished antibody isolated from a non-human primate immunized with WT PfCSP that binds to the NTD, CIS43 is a dual binder for the junctional epitope and major repeats, L9 is a dual binder for the minor epitope and major repeats, 311 binds the major repeats, and mAb15 binds the CTD^16^. VRC01 is an anti-HIV-1 antibody used as a negative control. **e** Immunization regimen and details. IV, intravenous; SPZ, sporozoite. **f** Parasite burden in the liver after challenge with transgenic sporozoites. R21 was used as a benchmark immunogen. Naive refers to uninfected negative control mice and max burden to infected positive control mice. *p < 0.1; **p < 0.01; ***p < 0.001; ****p < 0.0001 as calculated by Kruskal–Wallis test with multiple comparisons.

We expressed PfCSP so that we could use it as a benchmark antigen in our studies and noticed that the soluble wild-type (WT) protein provided low yield after purification from HEK293E cells (50 µg per liter culture; Fig. 1a, Supplementary Fig. 1a), and was sensitive to cleavage when left overnight at 4 °C^65^. We performed N-terminal sequencing of a degraded protein sample and found it was cleaved between _66_KKNSR_70_ and _71_SLGENDD_77_, within the PEXEL II sequence (_70_RSLGE_74_; ref. ^66^) (Supplementary Fig. 1b). Since the _66_KKNSR_70_ sequence preceding the cleavage site resembles a furin cleavage site, we used site-directed mutagenesis to mutate the positively charged residues (Lys and Arg) to serine and alanine (_66_SSNSA_70_; Supplementary Fig. 1b), similar to what was done for viral glycoproteins^67–69^. We expressed this construct, named C25-SAmut, and observed a 400-fold increase in protein expression to 20 mg/L (Supplementary Fig. 1c). However, a dimer peak during SEC was observed, likely due to an unpaired cysteine, and we further mutated C25-SAmut to generate SAmut by mutating the cysteine to serine as previously described^70^. SAmut expressed well (20 mg/L) and eluted during SEC as a single symmetric peak (Supplementary Fig. 1d). We produced a truncated form of this construct, SAmut-5/3 (Fig. 1a), which contains the 3D7 junctional region in place of the full repeat domain, to focus responses to the junctional epitope and minor epitopes targeted by CIS43 and L9 (ref. ^14^)_._ A panel of mAbs spanning the NTD, the repeat domain, and the CTD all bound SAmut-5/3 as expected, similar to soluble PfCSP (Supplementary Fig. 1e).

To evaluate the effect of multimerization on PfCSP immunogenicity, we used the SpyTag-SpyCatcher (ST-SC) “plug-and-display” technology^71^ to display SpyTagged SAmut-5/3 on a variety of self-assembling protein nanoparticles—C4b^72^, ferritin^73^, and I53-50 (ref. ^58^)—which present 7, 24, and 60 copies of SC, respectively. We expressed and purified SAmut-5/3-ST protein and the homomeric SC-nanoparticles (SC-C4b and SC-ferritin) separately and then conjugated the antigen to each SC-nanoparticle (Fig. 1b, c**;** Supplementary Fig. 1f, g). For the two-component SC-I53-50 nanoparticle, we conjugated SAmut-5/3-ST to the trimeric SC-I53-50A component, assembled the nanoparticle by adding the I53-50B.4PT1 pentamer^58^, and purified the assembled nanoparticle by SEC. SDS-PAGE revealed little unconjugated SC-bearing protein in each case, suggesting highly efficient conjugation (Supplementary Fig. 1f, g), and SEC and negative stain electron microscopy (nsEM) indicated that each nanoparticle immunogen retained the expected size and morphology after conjugation (Fig. 1b, c**;** Supplementary Fig. 1f, g). All of the mAbs in the panel described earlier bound each nanoparticle by ELISA, establishing that SAmut-5/3 retained its antigenicity when multimerized (Fig. 1d).

To evaluate the effect of multimerization and antigen copy number on immunogenicity and protection, we immunized groups of 10 C57BL/6 mice with a constant molar dose of antigen at weeks 0 and 3 using ALFQ adjuvant^74^ (Fig. 1e). ALFQ is a liposome-based adjuvant with a synthetic monophosphoryl lipid A analog (3D-PHAD^®^) and QS-21, similar to the composition of the AS01 adjuvant used with the RTS,S vaccine. We used R21 as a benchmark immunogen, also adjuvanting it with ALFQ as we could not access RTS,S/AS01B (Mosquirix^TM^) or R21/Matrix-M for these studies. We note that although R21 is typically adjuvanted with Matrix-M (Novavax; ref. ^75^), it has also been shown to be protective in combination with liposomal adjuvants containing QS-21 similar to ALFQ^76^. We intravenously challenged the mice at week 6 with 2000 transgenic *P. berghei* parasites that express PfCSP in place of endogenous PbCSP and GFP/luciferase for measuring liver burden (Pb-PfCSP-GFP/LUC)^77^. A clear trend in liver burden measurements taken 2 days after challenge suggested that increased antigen copy number may improve protection (Fig. 1f). However, none of the differences between the C4b, ferritin, or I53-50 nanoparticles or soluble antigen were statistically significant. Among the immunogens tested, only R21 and the I53-50 nanoparticle displaying ~60 copies of SAmut-5/3 significantly reduced liver burden compared to an unimmunized control group (“max burden”). R21 alone conferred significantly higher protection than the soluble SAmut-5/3. The superior performance of R21 may be due to the different antigen it displays (Fig. 1a) or other features of the HBsAg particle.

### Design and characterization of nanoparticle immunogens comprising the CSP junctional region

**Fig. 2:**
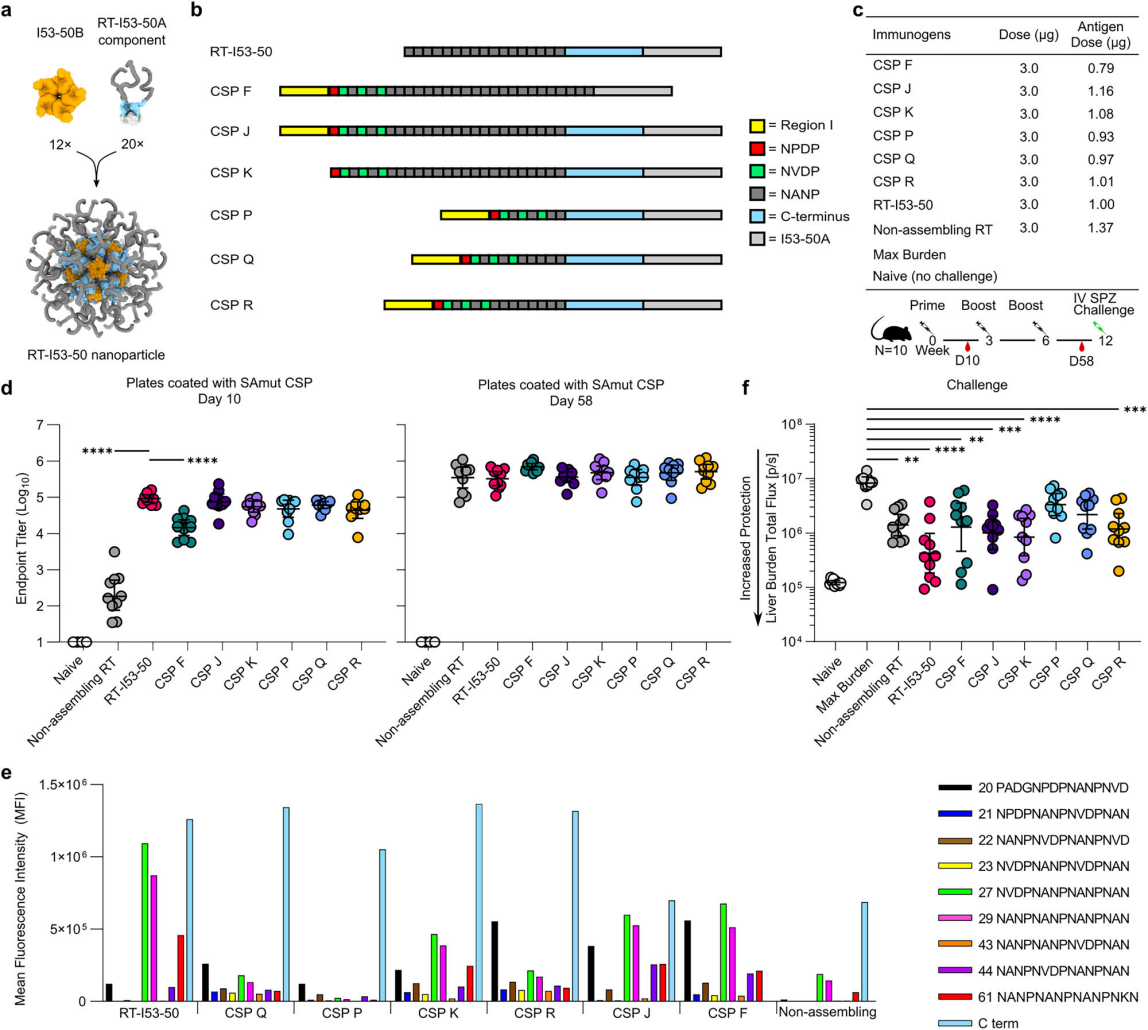
Design, characterization, and immunogenicity of nanoparticle immunogens comprising the CSP junctional region. **a** Models of the trimeric RT-I53-50A (RT in dark gray and blue, I53-50A in light gray) and pentameric I53-50B (orange) components, and an assembled RT-I53-50 nanoparticle. **b** Schematics of junctional region antigens. Each antigen was genetically fused to I53-50A. **c** Immunization regimen and details of the study. **d** Serum antibody titers against SAmut, determined by ELISA using sera obtained 1–2 weeks after the primary and third immunizations. Statistical significance was calculated by one-way ANOVA with multiple comparisons. **e** Peptide mapping ELISAs using pooled sera from each group, measured using mesoscale discovery (MSD) -multi-spot assay system. **f** Parasite burden in the liver after challenge with transgenic sporozoites. RT-I53-50 was used as the benchmark immunogen. *p < 0.1; **p < 0.01; ***p < 0.001; ****p < 0.0001 as calculated by Kruskal–Wallis test with multiple comparisons.

Given that the I53-50-based immunogen showed good protection in our initial study and this nanoparticle has proven capable of displaying a wide variety of antigens^60–62,78^, we selected I53-50 as a platform for iterative CSP-based nanoparticle vaccine design. Our overall aims were to evaluate the contribution of each region of CSP to immunogenicity and protection, and to evaluate whether variants of the repeat region could increase the likelihood of eliciting protective antibodies that bind the junctional and minor epitopes that are not present in RTS,S and R21 (refs. ^14–16^). We used genetic fusion for these studies rather than SC-ST conjugation to generate well-defined immunogens and to simplify our workflow by eliminating the need for a conjugation step. We began by genetically fusing the truncated repeat region and CTD of CSP found in RTS,S and R21 (i.e., the “RT” antigen) to I53-50A, the trimeric component of I53-50 (Fig. 2a, b). In vitro assembly of RT-I53-50A with I53-50B.4PT1 followed by preparative SEC yielded monodisperse nanoparticles of the expected size and morphology (Supplementary Fig. 2). We then replaced RT with a series of antigens that included the full NTD or Region I (RI), comprised several different variations of the central repeat region, and lacked the CTD (CSP A-H, Supplementary Table 1). Though the CTD contains T cell helper epitopes^79,80^, we explored whether it could be excluded because it has been shown that antibodies targeting it are weakly neutralizing or do not inhibit parasite traversal/development^81,82^. These designs all expressed but were prone to aggregation except for CSP F, the only design which contained a truncated N-terminal domain comprising only the RI (Fig. 2b). We were able to successfully assemble and characterize CSP F nanoparticles that closely resembled RT-I53-50 (Supplementary Fig. 2). We then tested whether similarly truncating the N-terminal domain in the other proteins would improve their solution properties, but the new constructs (CSP A2-H2; Supplementary Table 1) also aggregated and were not pursued further.

We then tested a new family of designs that included the CTD, as it appeared to be important for the solution properties of I53-50A fusion proteins displaying CSP-derived antigens. Four designs contained RI, the junctional region, and increasing numbers of major repeats (up to 17; CSP P, Q, R, and J), while a fifth was identical to CSP J except that it lacked the RI (CSP K; Fig. 2b). All of these constructs were successfully expressed in *E. coli*, purified, and assembled into nanoparticles by mixing with I53-50B.4PT1 pentamer. In each case, analytical SEC, dynamic light scattering (DLS), and nsEM revealed monodisperse populations with the expected morphology, and binding of mAbs specific to various regions of PfCSP showed the expected patterns (Supplementary Fig. 2). Interestingly, similar constructs that contained the entire repeat region of PfCSP failed to express (CSP L and M; Supplementary Table 1). In summary, we generated a series of nanoparticle immunogens displaying various CSP-derived antigens that would allow us to evaluate the contribution of each region of the protein to immunogenicity and protection.

### Immunogenicity of nanoparticle immunogens comprising the CSP junctional region

To evaluate immunogenicity and the protection afforded by this series of nanoparticle immunogens, groups of 10 mice were injected intramuscularly with 3 µg of each nanoparticle formulated with ALFQ, followed by two homologous boosts given 3 weeks apart (Fig. 2c). As benchmarks, mice were immunized with RT-I53-50 nanoparticles and non-assembled RT-I53-50A trimers. Serum was collected 1–2 weeks after each immunization and measured anti-SAmut titers by ELISA (Fig. 2d). Although the RT-I53-50 nanoparticles elicited significantly higher levels of antigen-specific antibodies than the RT-I53-50A trimer and CSP F nanoparticles after a single immunization, all groups had similar titers in the anti-SAmut ELISA after three immunizations.

To determine the epitopes targeted by vaccine-elicited antibodies, we conducted a peptide binding ELISA using pooled sera from each group of mice. We used a series of overlapping peptides, spanning the repeat region and CTD of PfCSP, which we refer to as peptides 20–61 and C-term (Fig. 2e; ref. ^14^). As expected, sera from mice vaccinated with immunogens containing the CTD (i.e., all except CSP-F) showed a strong response to this domain. Also as expected, serum antibodies from RT-I53-50-vaccinated mice had a strong preference for binding to NANP-containing peptides 27 (NVDPNANPNANPNAN), 29 (NANPNANPNANPNAN), and 61 (NANPNANPNANPNKN), but did not bind well to peptides containing the junctional epitope or minor repeats. Sera from mice that received immunogens containing the junctional region displayed more balanced binding across the set of peptides, although with varying magnitudes that roughly correlated with the total number of repeats in each immunogen. For example, CSP P elicited weak responses against repeat peptides while CSP Q and R showed stronger binding across all peptides tested, including peptide 20, which spans the junctional epitope (PADGNPDPNANPNVD). CSP F, J, and K, which included more copies of the major repeat, showed stronger binding that was more balanced than RT-I53-50 but skewed more toward the major repeats (i.e., peptides 27 and 29) than CSP Q and R.

Six weeks after the second boost, we challenged the mice intravenously with 2000 sporozoites and measured liver burden 2 days later by IVIS (Fig. 2f). Although all of the immunogens other than CSP P and Q provided significantly better protection than the max burden control group, only CSP K reached the same level of statistical significance as RT-I53-50. Considered together with the peptide ELISA data (Fig. 2e), these results indicate that immunogens with higher major repeat content (i.e., RT-I53-50 and CSP F, J, K, and R) induced better protection than those focused only on the junctional region or containing a reduced number of major repeats (i.e., CSP P & Q). Furthermore, the data show that the non-assembling RT-I53-50A trimer confers less protection than the RT-I53-50 nanoparticle, and that pre-challenge anti-SAmut ELISA titers alone cannot be used to reliably predict protection^83,84^. Overall, our data demonstrate that we were able to modulate the epitope specificities of vaccine-elicited antibodies by displaying various CSP-derived antigens on I53-50, but that none of the novel immunogens was able to induce better protection than our benchmark RT-I53-50 nanoparticle immunogen.

### Design, characterization, and immunogenicity of non-native CSP-repeat nanoparticles

**Fig. 3:**
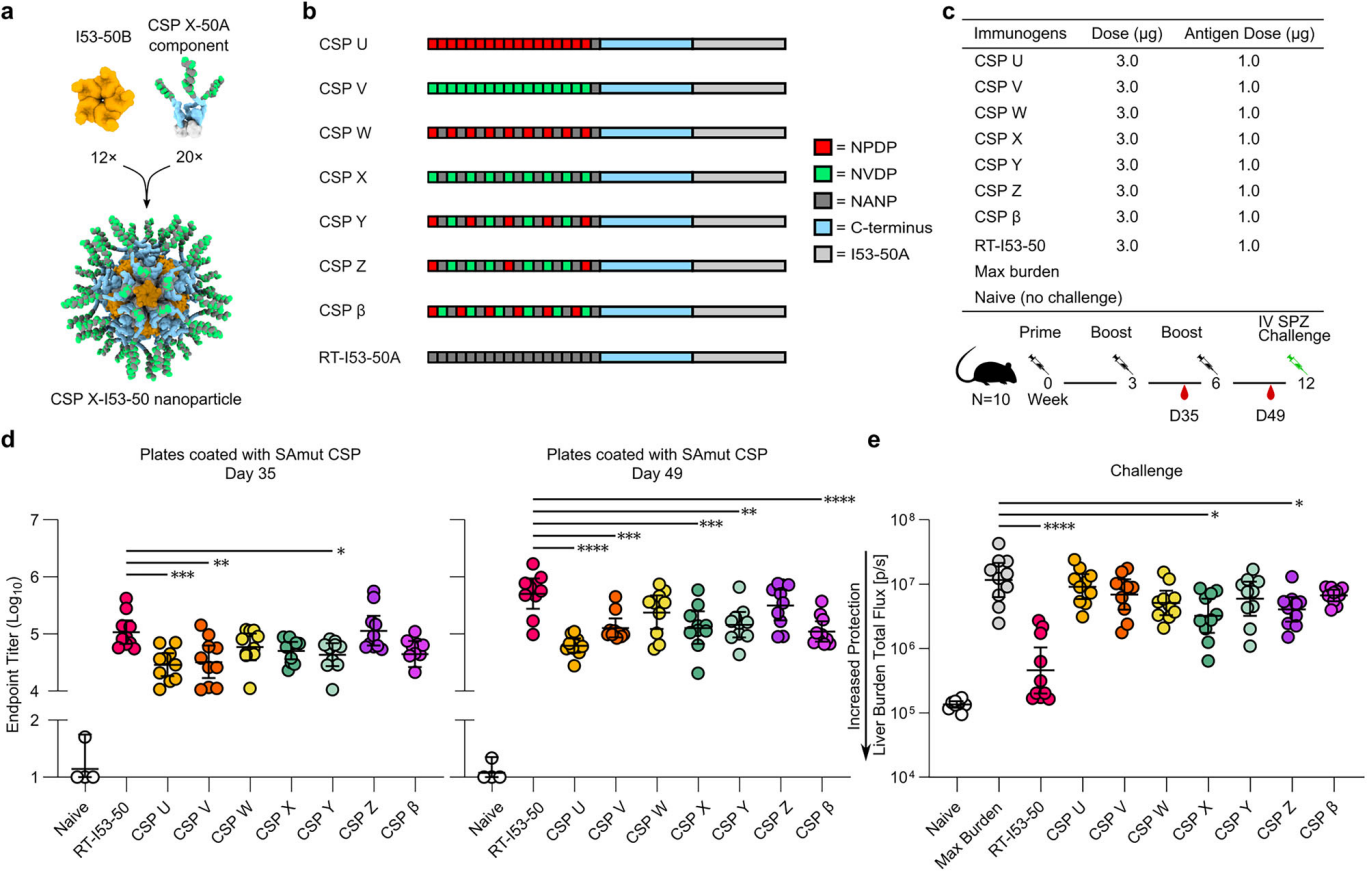
Design, characterization, and immunogenicity of non-native CSP-repeat nanoparticles. **a** Models of the CSP X-I53-50A trimer (CSP X in green, dark gray, and blue; I53-50A in light gray), I53-50B (orange), and an assembled CSP X-I53-50 nanoparticle. **b** Schematics of non-native CSP-repeat antigens. Each antigen was genetically fused to I53-50A. **c** Immunization regimen and details of the study. **d** Serum antibody titers against SAmut, determined by ELISA using sera obtained after the second and third immunizations. Statistical significance was calculated by one-way ANOVA test with multiple comparisons. **e** Parasite burden in the liver after challenge with transgenic sporozoites. RT-I53-50 was used as the benchmark immunogen. *p < 0.1; **p < 0.01; ***p < 0.001; ****p < 0.0001 as calculated by Kruskal–Wallis test with multiple comparisons.

We next designed a series of I53-50A trimers bearing non-native repeat-based antigens to attempt to further focus the vaccine-elicited immune response towards the junctional region or minor repeats (Fig. 3a, b). Each antigen in the series comprised 18 total repeats, always ending with a major repeat to provide a native-like junction with the C-terminal domain. We designed constructs that displayed alternating forms of the CSP junctional region (CSP Y, Z), alternating junctional-major or minor-major repeats (CSP W, X), completely non-native sequences that included junctional or minor repeats only (CSP U, V) as well as a tandem junctional-minor-major repeat antigen (CSP β). All of these I53-50A fusion proteins were expressed in *E. coli*, purified using IMAC and SEC, and mixed with I53-50B to generate nanoparticle immunogens. Analytical SEC, DLS, and nsEM again indicated the formation of monodisperse I53-50-based nanoparticle immunogens (Supplemental Fig. 3). Antigenicity was characterized by ELISA and showed that most of the mAbs in our panel bound each of the immunogens, though most notably a decrease or loss in binding for CIS43 was observed for CSP U, V, W, and β (Supplemental Fig. 4a).

Like our previous immunization study, we immunized groups of 10 mice three times intramuscularly with 3 µg of RT-I53-50 or each non-native repeat nanoparticle formulated with ALFQ adjuvant (Fig. 3b, c). In this instance, each group of mice received nearly the same number of moles of nanoparticle immunogen because of their nearly identical molecular weights. Anti-SAmut CSP titers measured by ELISA after the second and third immunizations showed that RT-I53-50 induced the highest CSP-specific antibody titers (Fig. 3d). CSP Z, which comprised two tandem repeats of the entire junctional region of PfCSP, elicited the highest SAmut CSP-specific antibody titers among the non-native repeat nanoparticles. As before, the mice were challenged with 2000 sporozoites six weeks after the second boost and parasite load in the liver was measured (Fig. 3e). RT-I53-50, CSP-X, and CSP-Z were the only three immunogens that significantly reduced liver burden compared to the max burden control group, with RT-I53-50 providing the best protection. These results may be explained by the fact that these immunogens contained more native-like repeat cadences, while the others had higher proportions of non-native sequences of repeats such as NANP-NPDP or NPDP-NVDP (Fig. 3b).

To map the epitopes targeted by antibodies elicited by these three protective immunogens, we used a variation of the peptide binding ELISA in which serum antibody binding to a combined peptide 20–23 or a major repeat peptide coated on the ELISA plate (PADGNPDPNANPNVDPNANPNVDPNAN and (NANP)_9_, respectively) competed with binding to free peptides pre-incubated with the pooled sera (Supplementary Fig. 4b, c). The peptides used for competition (peptides 20–61) spanned the repeat region of PfCSP. We first gauged the performance of the assay using three mAbs that bind the major, junctional, and minor repeat regions (mAb4 [ref. ^14^], CIS43, and L9, respectively) and found that the preferred peptide epitopes of each mAb competed with its binding to the plated antigens as expected. Specifically, mAb4 bound (NANP)_9_ more strongly than peptide 20–23 and exhibited the greatest reduction in binding in the presence of major repeat-containing peptides (peptides 27, 29, 61), while L9 bound peptide 20–23 more strongly and showed the greatest reduction in signal in the presence of peptides containing minor repeats (peptides 20, 21, 22, 23, 43, and 44). CIS43 bound both peptides strongly as expected^14^ and showed a clear and consistent rank-ordering of peptide competition (peptide 21 > 20, 23 > 27 > 22, 43 > 44 > 29 > 61). Based on these mAb benchmarking data, we concluded that the peptide competition assay provided a sensitive readout of epitope specificity. Serum antibodies elicited by CSP X and CSP Z bound both ELISA antigens roughly equivalently, whereas the sera from mice receiving RT-I53-50 clearly bound (NANP)_9_ more strongly than peptide 20–23 as expected. For all three immunogens, the rank-ordering of competing peptides was consistent across both ELISA antigens. Anti-RT-I53-50 sera were most strongly competed by peptides containing higher numbers of major repeats (peptides 27, 29, and 61) and less so by peptides from the junctional region (peptides 20, 21, 22, and 23). By contrast, peptides containing minor repeats and those from the junctional region most effectively prevented CSP X- and Z-elicited antibodies from binding to the plated antigens, respectively. For both of the latter two immunogens, the major repeat peptides (peptides 29, 43, and 61) provided the weakest competition.

In summary, our data show that although nanoparticles displaying non-native CSP repeat cadences can focus responses toward junctional and minor repeat epitopes, they do not elicit antibodies that reduce liver burden to the same extent as the same nanoparticle displaying the RT antigen.

### Immunogenicity and protection afforded by mosaic and cocktail nanoparticle immunogens

Previous analyses have indicated that the most potently protective anti-CSP mAbs tend to be those that bind the major repeats with high affinity while also cross-reacting with the junctional and minor epitopes^15,33,85^. To explore whether we could elicit protective levels of such cross-reactive antibodies by vaccination, we conducted another mouse immunogenicity and challenge study in which we compared a series of mosaic and cocktail nanoparticle immunogens based on CSP X, CSP Z, and RT-I53-50. Several studies over the last few years have evaluated mosaic nanoparticle immunogens that co-display multiple antigenic variants on the same nanoparticle for their ability to elicit B cell and antibody responses of greater breadth than monovalent nanoparticles or mixtures thereof (“cocktails”)^63,72,73,86–96^. Two-component assemblies like I53-50 facilitate the production of mosaic nanoparticles since multiple antigens can be co-displayed by simply adding I53-50B pentamer to mixtures of multiple different antigen-bearing I53-50A trimers^63,72,73,86–89,97,98^.

We generated mosaic nanoparticles co-displaying RT and CSP X, RT and CSP Z, or CSP X and Z at 50% valency (i.e., 30 copies) each, as well as a mosaic nanoparticle co-displaying all three antigens at 33% valency (i.e., 20 copies) by adding a molar equivalent of I53-50B pentamer to appropriate mixtures of antigen-bearing I53-50A trimer components. We also made corresponding cocktail immunogens (i.e., RT + X, RT + Z, and RT + X + Z) by individually assembling and purifying each monovalent nanoparticle and then mixing them together. Analytical SEC, DLS, and nsEM indicated that the mosaic and cocktail nanoparticle immunogens assembled as intended (Supplementary Fig. 5).

**Fig. 4:**
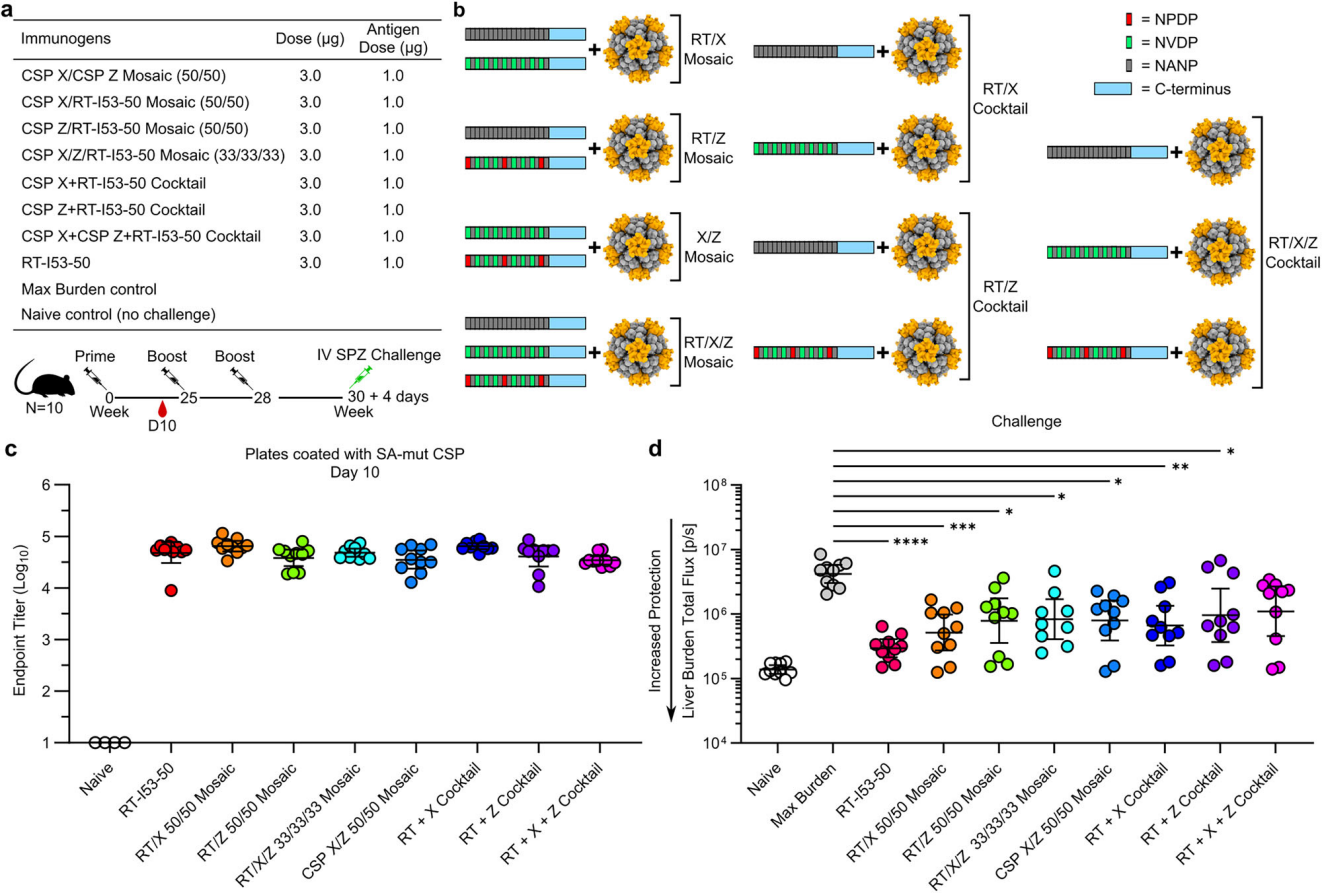
Immunogenicity and protection afforded by mosaic and cocktail nanoparticle immunogens. **a** Immunization regimen and details of the study. **b** Schematics depicting the antigenic composition of each immunogen: either mosaic nanoparticles with 33 or 50% valency of each antigen, or groups (cocktails) of monovalent nanoparticles. **c** ELISA endpoint titer of each immunogen to SAmut-coated plates post-prime. Statistical significance was calculated by one-way ANOVA test with multiple comparisons. **d** Parasite burden in the liver after challenge with transgenic sporozoites. RT-I53-50 was used as the benchmark immunogen. *p < 0.1; **p < 0.01; ***p < 0.001; ****p < 0.0001 as calculated by Kruskal–Wallis test with multiple comparisons.

Following our previous immunization regimens, we administered 3 µg of each mosaic or cocktail nanoparticle immunogen to groups of 10 mice intramuscularly with ALFQ adjuvant, followed by two boosts (Fig. 4a, b). We again included RT-I53-50 as a benchmark immunogen as well as naive and max burden control groups. Ten days post-prime, mice were bled and anti-SAmut CSP titers were measured by ELISA (Fig. 4c). All vaccine groups had similar anti SAmut CSP titers post-prime. Due to restrictions imposed during the COVID-19 pandemic, the study was put on hold after the primary immunization, resulting in an interval of 25 weeks between the prime and the first boost. Six weeks after the second boost, mice were challenged IV with 2000 sporozoites and parasite liver load was measured by IVIS (Fig. 4d). Despite having similar post-prime anti-SAmut CSP titers, the immunogens conferred various reductions in liver burden compared to the mock-immunized control. Unfortunately, due to COVID-19 related restrictions, immunogenicity data post-boost was not collected, preventing direct correlation of boost immunogenicity with observed liver burden. RT-I53-50 was again the most protective immunogen with the lowest liver burden (*p* < 0.0001), while the mosaic RT/X nanoparticle performed second-best (*p* < 0.001) and the cocktail RT + X nanoparticle performed third-best (*p* < 0.01). One potential reason for immunogens based on RT and CSP X being more protective compared to those based on CSP Z may be that they contain a higher proportion of native-like repeat cadences (e.g., NVDP-NANP and NANP-NANP vs. NANP-NPDP) and thus more protective epitopes, an interpretation that is also supported by our previous study (Fig. 3). Our data did not allow us to distinguish between the mosaic and cocktail immunogens; more detailed studies would be required to determine whether differences exist in the B cell and antibody responses elicited by each.

### Comparison of R21 and RT-I53-50 adjuvanted with ALFQ

As we repeatedly observed that RT-I53-50 conferred better protection than immunogens containing junctional or minor repeats, we compared it against R21 in a head-to-head immunogenicity and challenge study. We also included an I53-50 nanoparticle displaying full-length SAmut CSP as a genetic fusion to determine if inclusion of the NTD and the entire repeat region improved protection. A comparator group received soluble (i.e., non-particulate) SAmut CSP to control for the effect of multivalent display. The SAmut-CSP-I53-50A trimer was expressed in *E. coli*, purified by immobilized metal affinity chromatography (IMAC) and SEC, and assembled with I53-50B to form nanoparticles. The resultant SAmut-CSP-I53-50 nanoparticles were purified by SEC to remove residual components, and the purified assemblies were evaluated by DLS and analytical SEC, both of which indicated monodisperse nanoparticles of the expected size and morphology (Supplementary Fig. 6).

**Fig. 5:**
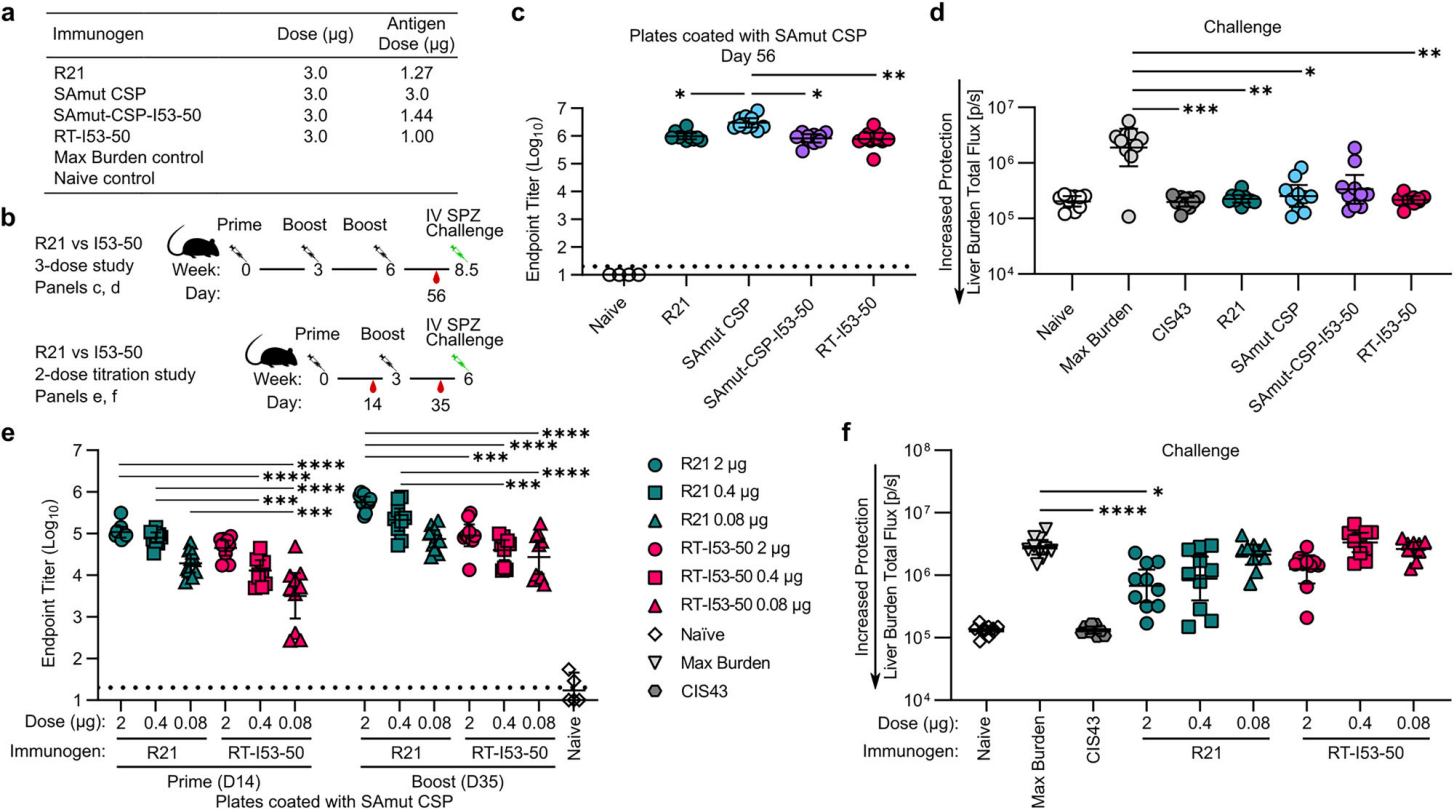
Comparison of R21 and RT-I53-50 adjuvanted with ALFQ. **a** Groups and doses used. **b** Immunization regimens and details for the three-dose and two-dose experiments. **c** ELISA endpoint titer for each immunogen in the three-dose study to SAmut-coated plates after the second boost and before the challenge. Statistical significance was calculated by one-way ANOVA test with multiple comparisons. **d** Parasite burden in the liver after three immunizations followed by challenge with transgenic sporozoites. For the CIS43 group, 300 μg of CIS43 was administered 2 h prior to infection. *p < 0.1, **p < 0.01, ***p < 0.001, ****p < 0.0001 as calculated by Kruskal–Wallis with multiple comparisons test compared to infected (max burden) control. **e** ELISA endpoint titer for each immunogen in the two-dose titration study to SAmut-coated plates after the prime and boost immunizations. Statistical significance was calculated by one-way ANOVA test with multiple comparisons. **f** Parasite burden in the liver after two immunizations followed by challenge with transgenic sporozoites. *p < 0.1; **p < 0.01; ***p < 0.001; ****p < 0.0001 as calculated by Kruskal–Wallis test with multiple comparisons compared to max burden control.

Groups of 10 mice were immunized intramuscularly three times with a 3 μg total protein dose of R21, SAmut CSP, SAmut-CSP-I53-50, or RT-I53-50 formulated in ALFQ (Fig. 5a, b). We also included a group that received 300 μg of CIS43 2 hours prior to infection as a fully protective control. Anti-SAmut CSP ELISA using sera obtained 2 weeks after the final immunization revealed that R21, RT-I53-50, and SAmut-CSP-I53-50 induced similar levels of anti-CSP antibodies, all three of which were lower than monomeric SAmut CSP (Fig. 5c). Following challenge, immunization with R21, RT-I53-50, and monomeric SAmut CSP all significantly reduced liver burden compared to the max burden control group, with R21 and RT-I53-50 exhibiting the greatest levels of liver burden reduction using this immunization regimen (Fig. 5d).

To attempt to resolve potential differences in the levels of protection provided by R21 and RT-I53-50, we conducted another study in which we lowered the total protein dose to 2, 0.4, and 0.08 μg and reduced the number of immunizations to two (Fig. 5b). At each dose, R21 elicited higher levels of anti-SAmut CSP serum antibody titers post-prime and -boost, with comparable titers between R21 at 0.08 μg and RT-I53-50 at 2 μg (corresponding to 0.03 and 0.66 μg of RT antigen, respectively; Fig. 5e). This result was consistent with the parasite challenge results, with R21 at 2 μg being the only group other than the CIS43 mAb-treated control that significantly reduced liver burden compared to the mock-immunized group (Fig. 5f). Altogether, our data show that although immunization with RT-I53-50 and R21 in ALFQ induces equivalently robust protection after 3 doses, R21 is more protective at lower doses and using a reduced number of immunizations.

## Discussion

Here we analyzed the functional effects of including N-terminal, junctional region, minor, and major repeat epitopes in CSP subunit and protein nanoparticle vaccines. Our work complements and extends recent studies that have evaluated peptide-based antigens displayed on self-assembling carrier proteins or nanoparticles^39–41,43,44,52^. These studies have focused on one or a few antigens comprising epitopes from the junctional region of CSP, either alone or in combination with major repeat sequences. We employed an iterative, empirical approach in which we attempted expression of 130 constructs and evaluated liver burden protection offered by 27 different CSP-based immunogens. These immunogens displayed many different native-like or synthetic CSP-derived sequences and were compared head-to-head in a stringent intravenous sporozoite challenge model. In each challenge study, we included either RT-I53-50 or R21 so that we could compare the experimental antigens against high-performing benchmark immunogens. This strategy allowed us to directly evaluate the contribution of each region of CSP to protective immunity and explore approaches to CSP-based antigen design that had not previously been tested to our knowledge. Our data support several conclusions that may help guide the design of next-generation CSP-based vaccines.

First, by comparing a series of nanoparticles displaying the same total amount of stabilized CSP-derived antigen, but in different numbers of copies on each nanoparticle, we established a correlation between increased antigen valency and improved protection against sporozoite challenge. This result is consistent with previous work from our groups and others using viral glycoprotein antigens^48,60,99,100^, though it should be noted that different T cell epitope content between C4b, Ferritin, and I53-50 also may have contributed to differences in protection. In our experiments, R21 also provided better protection than the series of SAmut-5/3 nanoparticle immunogens we tested, indicating that antigen valency is not the only determinant of immunogenicity and protection. Other salient differences between R21 and the other nanoparticles include the different T cell epitope content in each nanoparticle^101^, the fact that HBsAg is a lipoprotein complex^102,103^, and the displayed antigens. Regarding the latter, SAmut-5/3 contains only 8 repeats (4 major, 3 minor, 1 junctional), while R21 contains 18 major repeats, which may improve B cell receptor cross-linking, B cell activation, and the induction of anti-CSP antibodies^47^. It is also possible that inclusion of the N-terminal region of CSP in SAmut-5/3 interferes with elicitation of protective anti-repeat antibodies, as this highly disordered^104^ and weakly immunogenic domain is presumably the most exposed portion of the antigen when displayed on nanoparticles.

Second, although we found that including the junctional region and minor repeats in I53-50-displayed antigens successfully induced antibodies targeting these epitopes, these immunogens consistently underperformed against parasite challenge compared to RT-I53-50, which displayed only major repeats. Furthermore, by directly comparing a series of nearly identical nanoparticle immunogens that comprised variable numbers of major repeats, we observed a correlation between increasing numbers of major repeats and improved protection. As discussed above, this correlation may be explained by improved B cell receptor cross-linking or major repeat epitope accessibility in the nanoparticles containing more major repeats. The latter explanation is supported by our observation that RT-I53-50 conferred better protection than nanoparticle immunogens comprising more major repeats that also included the junctional region and N terminus (CSP F, J, and K). It should also be noted that despite differences in liver burden reduction (Fig. 2f), anti-SAmut titers between junctional region-containing nanoparticles and RT-I53-50 were equivalent before challenge (Fig. 2d). This may be due to differences in antibody avidity between groups and is a limitation of our study that will need to be addressed in future experiments. The importance of the NANP repeat as a central motif in the epitopes of anti-repeat antibodies^33^ was further highlighted by our non-native CSP repeat cadence nanoparticles, which again were outperformed by the benchmark RT-I53-50 nanoparticle in challenge studies. CSP X and Z were the most native-like antigens in this series, comprising repeated instances of the NVDP-NANP cadence observed in the junctional region of CSP, and were the only two that significantly reduced liver parasite burden, although not as significantly as RT-I53-50.

Although several groups have reported the induction of protective responses by displaying junctional region epitopes on various nanoparticle platforms^41,44,52^, few have actually done direct, side-by-side comparisons against similar vaccines that comprise only major repeats^39,42,105^. This complicates direct comparisons between studies, as do several other differences in the models used to evaluate protection. For example, mosquito bite challenge may be less stringent than the intravenous challenge model we used here, but is a more physiologically relevant challenge route that may discern contributions from antibodies that cross-react with the junctional region and have distinct activities in the skin^106^ is a limitation of this study and will need to be addressed in the future. Furthermore, it is possible that the murine antibody repertoire is not suited to producing antibodies with mechanisms similar to antibodies targeting the junctional region^15^, which could lead to underestimation of the performance of immunogens containing the junctional and minor epitopes. We suggest that future studies of novel CSP-based vaccines include R21 as a benchmark immunogen whenever possible.

Third, we found that the CTD was beneficial for the manufacturability and immunogenicity of our nanoparticle vaccines. There is evidence that antibodies against the CTD of CSP correlate with protection^84,107,108^, but such antibodies appear to be rarely elicited by immunization with sporozoites^81^, are weakly protective as mAbs^82,109^, and may be susceptible to escape by antigenic variability^81,110^. Interestingly, we found that most of our designed immunogens lacking the CTD did not express solubly in *E. coli* or were prone to aggregation, suggesting that CTD has a stabilizing effect. The one nanoparticle immunogen we were able to produce without the CTD, CSP F, elicited significantly lower antibody titers post-prime than the RT-I53-50 comparator. This result suggests that a significant fraction of the antibody response elicited by the other immunogens was directed at the CTD (which is supported by our epitope mapping data), that known T cell epitopes in the CTD augment the vaccine-elicited antibody response^111^, or both. We note that other next-generation CSP-based vaccine candidates do not contain the CTD^39,40,52^. Our data recommend its inclusion, although its potential elicitation of strain-specific antibodies will need to be addressed.

Finally, comparing our I53-50-based nanoparticle immunogens to R21 indicated that properties other than the displayed antigen significantly contribute to anti-malarial immunity. RT-I53-50 reduced liver burden after challenge equivalently to R21 after three doses, establishing it as a benchmark comparator. SAmut and SAmut-I53-50 showed weaker protection, with the latter potentially being affected by N-terminal interference or differences in nanoparticle stability. A follow-up two-dose titration study between RT-I53-50 and R21 showed that R21 was superior by anti-SAmut titer and liver burden reduction at all doses compared to RT-I53-50. As the CSP-based antigen displayed by R21 and RT-I53-50 in these studies was identical, the differences observed must be due to other factors, potentially including antigen copy number^48,60,99^, display geometry^112^, T cell epitope content^40,113^, or potential adjuvanting effects of the R21 lipoprotein complex^102^. Our head-to-head comparisons, combined with recent reports from others demonstrating robust protection from CSP-based nanoparticle vaccines that include exogenous T cell help and N-linked glycans^40^, suggest that engineering these additional features into I53-50-based vaccines could improve their performance. These features may be particularly important for antigens like PfCSP that lack N-linked glycans and have substantially reduced sequence complexity compared to many viral glycoprotein antigens, and may partly explain why RT-I53-50 did not perform as well as R21.

In conclusion, we extensively explored multivalent display of CSP-based antigens on self-assembling nanoparticle scaffolds. Our benchmarked studies identified several antigen design approaches that are likely not worth exploring further, such as designing non-native repeat-based sequences, while highlighting other approaches that merit further consideration. Combining these approaches on a clinically validated nanoparticle platform like I53-50 may yield an optimal CSP-based immunogen, which will be the key component of next-generation vaccines that target multiple stages of the parasite life cycle^114^.

## Methods

### PfCSP plasmid construction and mutations

The plasmid construction of WT PfCSP was described previously^14^. Briefly, mammalian codon-optimized PfCSP was cloned into a CMV/R-expression system with a C-terminal Avi-Tag, HRV3C cleavage site, and a 6x His-tag (GenScript). Mutations to CSP and repeat truncations were generated using site-directed mutagenesis with the QuikChange XL kit (Agilent). In brief, the K66S, K67S, and R70A mutagenesis was performed on the PfCSP plasmid followed by the C25S mutagenesis. The PfCSP-C25S-SAmut-19/3 truncation was generated from the full length plasmid. The PfCSP-C25S-SAmut-5/3 truncation was generated from the PfCSP-C25S-SAmut-19/3 plasmid.

### PfCSP expression and purification

PfCSP or mutants were expressed through transient transfection in HEK293E cells (National Research Council of Canada (under license)) using the Freestyle 293 F expression system at 37 °C, 6% CO_2_ for 6 days. Protein was purified from culture supernatants using Ni-NTA affinity resin followed by size exclusion chromatography (SEC) using a HiLoad 16/600 Superdex 200 pg column (Cytiva). Fractions containing protein were pooled, concentrated, flash-frozen in liquid nitrogen, and stored at −80 °C.

### N-terminal sequencing

N-terminal Edman sequencing was performed at the Protein and Nucleic Acid (PAN) Biotechnology Facility at Stanford.

### SAmut-5/3-SpyTag expression and purification

A codon-optimized gBlock (IDT) of the last 100 bp of CSP-SAmut with a C-term 6xHisTag and SpyTag was synthesized. The gBlock was amplified using Platinum SuperFi II PCR Master Mix (Invitrogen), and PfCSP-C25S-SAmut-5/3 amplified similarly. The construct was assembled with the Infusion HD Cloning Plus kit (Takara Bio). Primer designs are available in the Key Resources Table. The construct was transformed into NEB5α *E. coli* cells (New England BioLabs) and DNA isolated for transfection by MidiPrep (Qiagen). The construct was expressed in HEK293E cells (National Research Council of Canada (under license)), with 500ug DNA, 2 mg PEI, and 38 mL PBS per 1 L of culture at 1 million cells/mL. Cultures were harvested after 6 days at 37 °C, 5% CO_2_, and 140 rpm. Supernatant was sterile filtered (0.22 µm), batch bound to Ni-NTA resin overnight at 4 °C and 120 rpm, and eluted with 5 mM Tris buffer containing 300 mM imidazole. The complex was concentrated using a 10 kDa Amicon® (Millipore Sigma) spin column, sterile filtered (UltraFree-CL, Millipore Sigma), and injected onto a Superdex 200 16/600 column (Cytiva) equilibrated in HEPES (5 mM HEPES, 150 mM NaCl, pH 7.5). Purified protein was concentrated, and flash frozen for long term storage.

### SpyTag CSP-SC-I53-50 NP assembly

Purified CSP-SAmut-5/3 protein with SpyTag was mixed with I53-50A-Spy-Catcher subunit in a 1:1 molar ratio and incubated overnight at 4 °C. The complex was purified over a Superdex 200 16 × 600 column (GE healthcare) in 5 mM HEPES, 150 mM NaCl, pH 7.5. The complex was sterile filtered (UltraFree-CL, Millipore Sigma) and combined with an equal molar ratio of I53-50B and incubated overnight at 4˚C to form nanoparticles. As needed, L-arginine up to 150 mM was added to the nanoparticles to prevent aggregation. Nanoparticles were applied to a Superose 6 increase 10/300 GL column (GE healthcare) in 5 mM HEPES, 150 mM NaCl, pH 7.5 buffer to isolate the nanoparticles. Concentrations. were measured with a UV-Vis spectrometer and flash frozen in liquid nitrogen for long term storage.

### SpyTag CSP-SC-Ferritin NP assembly

Purified ferritin nanoparticles with a 6x-Histag and SpyCatcher were mixed with purified SpyTag CSP-SAmut-5/3 protein in a 1:1 molar ratio of CSP to Ferritin/SpyCatcher particle subunit. 50mM L-arginine was added to prevent precipitation, and the conjugation was allowed to run overnight at 4˚C. The complex was purified over a Superose 6 Increase 10/100 GL column in 5 mM HEPES, 150 mM NaCl, pH 7.5 buffer, and concentrated using a 30 kDa Amicon® (Millipore Sigma) spin column before flash freezing in liquid nitrogen.

### SpyTag CSP-SC-C4b NP assembly

Purified C4b-SpyCatcher nanoparticles were mixed in a 2:1 molar ratio of CSP:C4b in HEPES buffer (in 5 mM HEPES, 150 mM NaCl, pH 7.5 buffer) and the reaction was allowed to go overnight at 4 °C. The complex was purified over a Superdex 200 16 × 600 column in 5 mM HEPES, 150 mM NaCl, pH 7.5 buffer. The complex was concentrated using a 30 kDa Amicon® (Millipore Sigma) spin column and flash frozen in liquid nitrogen for storage.

### IgG expression and purification

Recombinant IgG were expressed through transient transfection in HEK293E cells (National Research Council of Canada (under license)) using the Freestyle 293 F expression system at 37 °C, 6% CO_2_ for 6 days. IgG was purified from cell culture supernatant using Protein A resin (GoldBio) and eluted from resin using IgG Elution Buffer (Pierce). IgG were further purified by SEC using HiLoad 16/600 Superdex 200 pg column (GE Healthcare* now Cytiva).

### PfCSP peptides

All peptides for this study were directly synthesized and biotinylated by GenScript. These include linear peptides numbered 20–61 that were 15 amino acids in length and overlapped by 11 residues spanning the central repeat region of PfCSP, a 36mer peptide (NANP)_9_, a 27mer peptide 20–23 (PADGNPDPNANPNVDPNANPNVDPNAN), and C-Terminal domain.

### Antigenicity and immunogenicity ELISAs

96-well Immulon 2HB (Thermo Scientific) microtiter plates were coated with 50 ng/mL of PfCSP or mutant overnight at 4 °C. Plates were washed 4X with phosphate-buffered saline (PBS) with 0.02% Tween-20 (wash buffer). Plates were blocked with 250 μL of 10% non-fat milk and 0.02% Tween-20 in PBS (blocking buffer) for 1 h at 37 °C. Plates were washed 4X with wash buffer. CSP-binding monoclonal IgG or VRC01, a HIV Env binding antibody used as a negative control, was diluted to 20 μg/mL in blocking buffer and added to the first row of plate. IgG was diluted in tenfold serial dilutions in blocking buffer and incubated for 1 h at 37 °C. After washing 4X with wash buffer, goat anti-human Ig-HRP (Southern Biotech) was added at a 1:3000 dilution and incubated at 37 °C for 1 h. Plates were washed 4X with wash buffer and 50 μL of SureBlue Reserve TMB Peroxidase Substrate (SeraCare) was added and incubated for 4 min followed by addition of 100 μL of 1 N H_2_SO_4_. The optical density at 450 nm was measured using a SpectraMax M2 plate reader (Molecular Devices). Wash steps were performed using a BioTek 405 Select microplate washer. Immune responses to full-length SAmut-CSP were measured by ELISA as described above using individual mouse sera (serum from each mouse was diluted in blocking buffer to 1:20 with 10-fold serial dilutions).

### Epitope mapping and competition ELISAs

Epitope mapping of the immune responses was performed with C-terminal domain (Genscript) and linear overlapping peptides (peptides 20–61; Genscript) that span the central repeat region of PfCSP using the MSD U-Plex Assay platform (Meso Scale Discovery) according to the manufacturer’s instructions. Briefly, MSD Gold microtiter plates (Meso Scale Discovery) were blocked with PBS + 5% BSA (20 µl/well), then coated with 10 µl/well of biotinylated peptides (240 pmol, Genscript) in PBS + 1% BSA for 1 h at room temperature. The coated plates were incubated for 2 h at room temperature with 10 µl of PfCSP control mAbs (5×10^−7^–5.0 µg/mL) or polyclonal mouse sera (pooled per group then diluted in blocking buffer to 1:20 with 10-fold serial dilutions). Plates were then incubated for 1 h at room temperature with 10 µl/well of 1.0 µg/mL of appropriate secondary (either anti-human or anti-mouse) IgG SULFO-TAG (Meso Scale Discovery) in PBS with 1% BSA/0.05% Tween-20. Plates were washed five times with PBS-Tween between each step. After a final wash, 35 µl of 1X MSD Read T Buffer (Meso Scale Discovery) was added to each well and plates were analyzed on an MSD Sector Image 2400 instrument.

Competitive ELISA was also performed using peptides 20–61. Briefly, streptavidin-coated plates (Meso Scale Discovery, MSD) were blocked with 5% BSA in PBS for 30 min at room temperature, washed five times (wash buffer, 0.05% Tween-20 in PBS), then coated for 1 h at room temperature with biotinylated-recombinant PfCSP (0.2 mg/mL, Genscript), peptide 20–23 (PADGNPDPNANPNVDPNANPNVDPNAN) or repeat peptide (NANP)_9_ (240 pmol, Genscript) in PBS with 1% BSA. PfCSP control mAbs (all at 10 ng/mL), or polyclonal mouse sera (pooled per group then diluted 1:250) were preincubated with varying concentrations (0–1000 ug/mL) of selected PfCSP peptides in PBS with 1% BSA/0.05% Tween-20 for 2 h at 37 C, then added onto the rPfCSP or peptide- coated plates. ELISA was performed on the MSD platform as described above.

### Expression and purification of CSP I53-50A immunogen designs

Designs were codon-optimized in pET29b, containing a C-terminal His-tag, for expression in BL21 (DE3) or LEMO21 cells. They were transformed and transferred to ZY Autoinduction medium (ZY media, 50XM Salts, 50 × 5052, 1 M MgSO4, and 1000X Trace metals). Cultures were incubated for 2 h at 37 °C, then the temperature was reduced to 18 °C overnight. Cultures were then centrifuged at 3500 rpm at 4 °C for 20 min in a Beckman Avanti JXN26 (Brea, CA, USA). The supernatant was discarded, and the pellet was resuspended in a buffer containing 50 mM Tris, 500 mM NaCl, 1 mM DTT, 30 mM imidazole, pH 8.0, 1 mM PMSF, 10 µg/mL DNase. The suspension was homogenized and run through a Microfluidizer (M-110P, Microfluidics, USA) to lyse and collected in a 50 mL conical. Conicals were then centrifuged at 4 °C for 30 min at 4000 rpm and the supernatant was transferred to a column containing Ni-NTA resin (Qiagen, Venlo, Netherlands), pre-equilibrated with Wash Buffer (25 mM Tris pH 8.0, 500 mM NaCl, 30 mM Imidazole). The resin was washed with one column volume of wash buffer and the protein eluted with a buffer containing 50 mM Tris, 500 mM NaCl, and 500 mM imidazole. Protein solution was concentrated and underwent size exclusion chromatography (SEC) on a Superdex 200 (GE Healthcare, Chicago, Il, USA) in a buffer containing 50 mM Tris, 500 mM NaCl. Fractions containing pure protein as controlled on SDS-PAGE were pooled and concentrated for further use.

### CSP-I53-50 NP assembly

CSP-I53-50A fusion proteins were sterile filtered using a 0.22 μm spin column and combined with an equal molar ratio of I53-50B and incubated for 1 hour at room temp or overnight at 4 °C with light agitation to form nanoparticles. Nanoparticles were applied to a Superose 6 increase 10/300 GL column (GE healthcare) in 50 mM Tris, 500 mM NaCl to remove unassembled components. Protein concentrations were measured using a UV-Vis spectrometer, diluted to the concentration needed for mouse immunization and flash frozen in Liquid nitrogen for long-term storage.

### Negative-stain EM

CSP-I53-50 NPs were added to carbon-covered 300 or 400 mesh copper grids and stained with 2% uranyl formate. Micrographs were imaged on a Tecnai F12 Spirit microscope with a 4k FEI Eagle CCD or a Talos 120 C microscope with a Ceta camera. Leginon and Appio were used to collect and process micrographs. Class average examples were generated using the 2D Classification job in CryoSparc v3.3.1.

### Dynamic Light Scattering (DLS)

Dynamic light scattering (DLS) was performed to determine the hydrodynamic radii and polydispersity (PDI) of purified CSP-I53-50 immunogens. Measurements were performed on either a Wyatt DLS using a 10 mm quartz cuvette or an UNcle Nano-DSF (UNchained Laboratories) using quartz capillary cassette (UNi, UNchained Laboratories). Four to ten acquisitions were collected using auto attenuation of the laser.

### Mice

Six- to eight-week-old female B6(Cg)-Tyrc-2J/J albino (B6-albino) mice were procured from The Jackson Laboratory. These animals were maintained in facilities accredited by the American Association for Accreditation of Laboratory Animal Care and cared for according to their standards. All procedures involving animals were approved by the Institutional Animal Care and Use Committees of the National Institute of Allergy and Infectious Diseases, National Institutes of Health, (Animal Study Protocols VRC-17-702 and VRC-20-0855).

### Parasites and mosquitoes

Transgenic *Plasmodium berghei* (strain ANKA 676m1C11, MRA-868) parasites expressing full-length (3D7 strain) *P. falciparum* CSP and a green fluorescent protein/luciferase fusion protein (Pb-PfCSP-GFP/LUC SPZ) were propagated and used to evaluate the efficacy of the PfCSP-based vaccines, as previously described^115^. Briefly, BALB/c mice were infected by intraperitoneal (IP) injection of Pb-PfCSP-GFP/LUC SPZ-infected RBCs. *Anopheles stephensi* (Nijmegan) mosquitoes were reared at the Laboratory of Malaria and Vector Research (National Institute of Allergy and Infectious Diseases, National Institutes of Health). Female mosquitoes were allowed to feed on the parasitized mice which were anesthetized by IP injection of ketamine (50 mg/kg) and xylazine (10 mg/kg) mixture. After feeding, mice were euthanized via CO_2_ inhalation, followed by cervical dislocation. Blood fed mosquitoes were then maintained in a humidified incubator at 19–20 °C and supplied with 10% sucrose. For challenge studies, sporozoites were harvested from mosquito salivary glands at day 18–21 after an infectious blood meal, as previously described^115^.

### Immunizations and SPZ IV challenge

Nanoparticle immunogens and R21 were diluted in sterile PBS to the indicated doses 0.08–3 µg and formulated with ALFQ, a liposomal adjuvant formulation containing 3D-PHAD^®^ and QS-21^74^, in a final volume of 50 µL^116^. Female B6-albino mice were immunized intramuscularly in the quadriceps at weeks 0, 3, and 6 (or as indicated in the figure). Serum samples were collected via tail veins at the indicated time points (10 days after 1st, and 2 weeks after 2nd and 3rd immunizations) to assess immunogenicity.

Challenges were conducted 2–6 weeks after the final immunization as indicated, where mice were challenged intravenously via the tail vein with 2000 freshly harvested Pb-PfCSP-GFP/LUC sporozoites^115^. Then, 40–42 hours post-challenge, mice received an IP injection of 150 µL of D-Luciferin (30 mg/mL), were anesthetized with isoflurane (5% for induction, 1–3% for maintenance). Luciferase activity in mice was visualized through imaging of whole bodies using the IVIS® Spectrum in vivo imaging system (PerkinElmer) 10 minutes post-injection. A group of uninfected control mice were referred to as naive, and infected control mice as max burden were used for each challenge experiment. Upon completion of the experiments, mice were euthanized via CO_2_ inhalation followed by cervical dislocation. To measure the burden of parasite infection in the liver, a region of interest (ROI) in the upper abdominal area (at the location of the liver) was analyzed and the total flux or bioluminescent radiance (photons/sec) emitted by Pb-PfCSP-GFP/LUC-SPZ was calculated using the manufacturer’s software (Living Image 4.5, PerkinElmer).

### Nanoparticle modeling and images

Nanoparticle models were generated from AlphaFold2^117^ predictions for CSP I53-50A components and the crystal structure for I53-50B^58^ arranged in icosahedral symmetry. Images were generated using ChimeraX molecular modeling software^118^.

## Supplementary information

Supplementary information

Supplementary Table 1

## Data Availability

All data are available in the manuscript or the supplementary materials. Further information and requests for resources and reagents should be directed to and will be fulfilled by the corresponding authors (mpancera@fredhutch.org and neilking@uw.edu).
