# Supplementary material for: Elicitation of liver-stage immunity by nanoparticle immunogens displaying P. falciparum CSP-derived antigens
Source: NPJ Vaccines. 2025 May 5;10:87. doi: 10.1038/s41541-025-01140-x (PMC12053698; doi:10.1038/s41541-025-01140-x)
Supplement: Supplementary file 1 — Supplementary information [file 41541_2025_1140_MOESM1_ESM.pdf]

Supplementary Figures

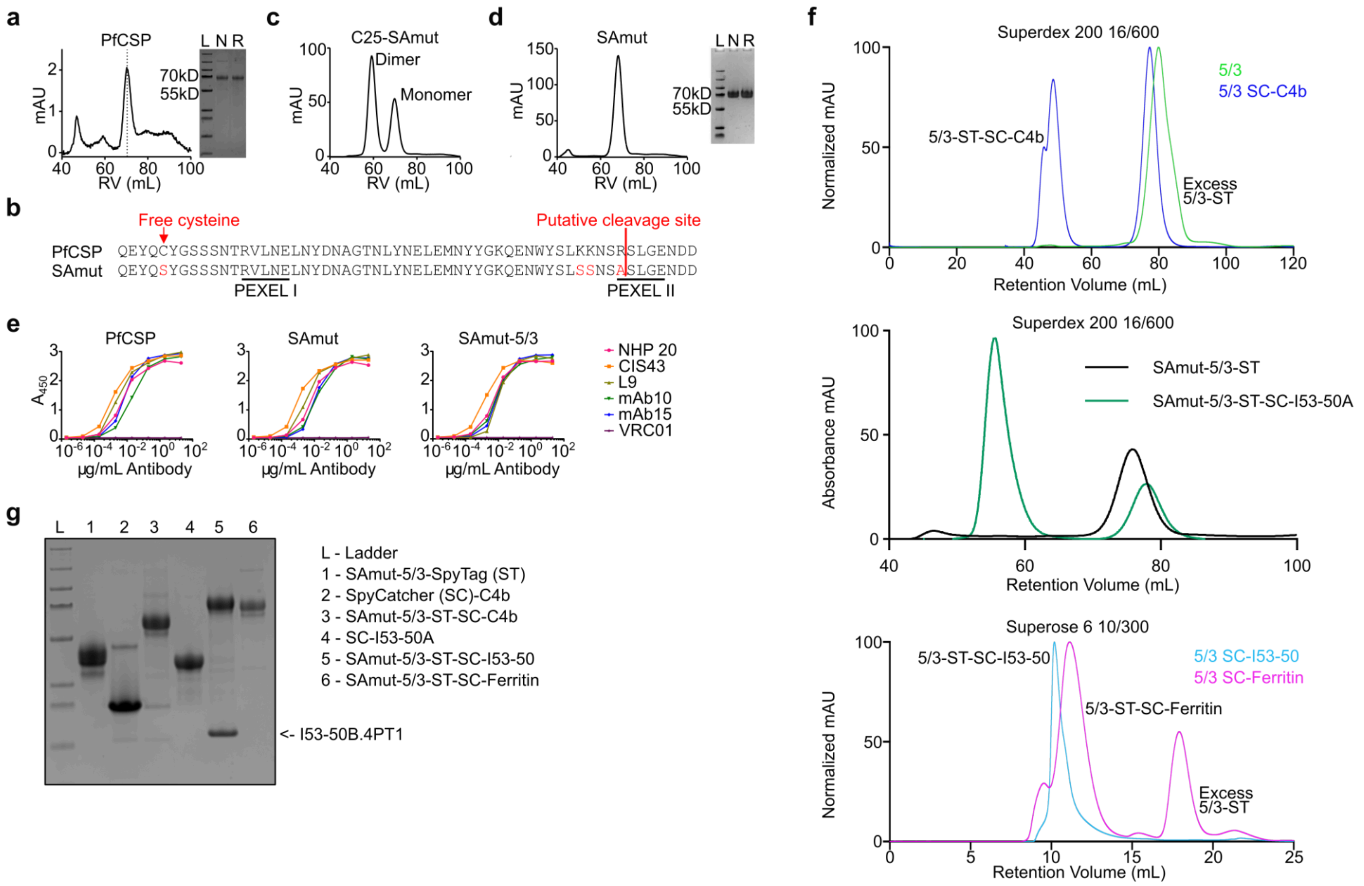

Supplementary Figure 1. Characterization data for stabilized SAmut-5/3 on nanoparticles. a SEC and SDS-PAGE of WT PfCSP. L, ladder; N, non-reduced; R, reduced. **b** Sequences of the WT PfCSP and SAmut NTDs. **c** SEC and SDS-PAGE of C25-SAmut and **d** SAmut. **e** Binding of WT PfCSP and SAmut variants to PfCSP-directed mAbs measured by ELISA. NHP20 binds the NTD, CIS43 is a dual binder for the junctional epitope and major repeats, L9 is a dual binder for the minor epitope and major repeats, mAb10 is a major repeat-only directed antibody, and mAb15 binds the CTD. VRC01 is an anti-HIV-1 antibody used as a negative control. **f** SEC profiles of each multimer. Overlaid SEC chromatograms of unconjugated SAmut-5/3-ST and conjugated SAmut-5/3-ST-SC-C4b (top), unconjugated SAmut-5/3-ST and conjugated SAmut-5/3-ST-SC-I53-50A component (middle), and for SAmut-5/3-ST-SC-HpFerritin and assembled SAmut-5/3-ST-SC-I53-50 nanoparticles. **g** SDS-PAGE of SpyCatcher (SC) and SpyTag (ST) constructs before and after conjugation.

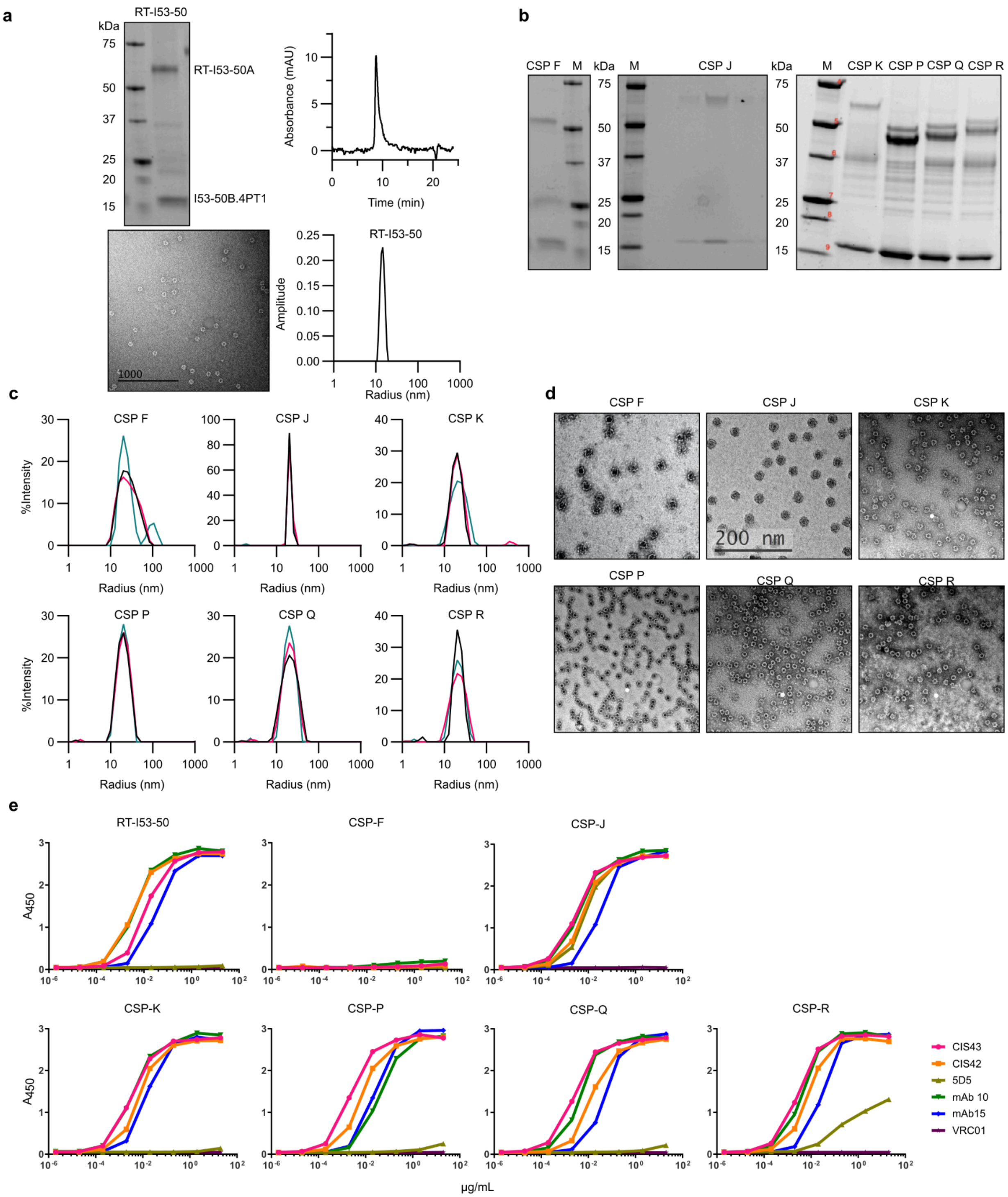

Supplementary Figure 2. Characterization data for I53-50 nanoparticle immunogens comprising the CSP junctional region. a (Clockwise, from upper left) SDS-PAGE, SEC, nsEM, and DLS of purified RT-I53-50 nanoparticles used in Figure 3. **b** SDS-PAGE, **c** DLS, and **d** nsEM for additional nanoparticle immunogens used in Figure 3. **e** Antigenicity ELISA curves for each nanoparticle immunogen against a panel of mAbs targeting the junctional epitope (CIS43 & CIS42), Region I (5D5), the major repeats (mAb10), and the C-terminal domain (mAb15). VRC01 was used as a negative control.

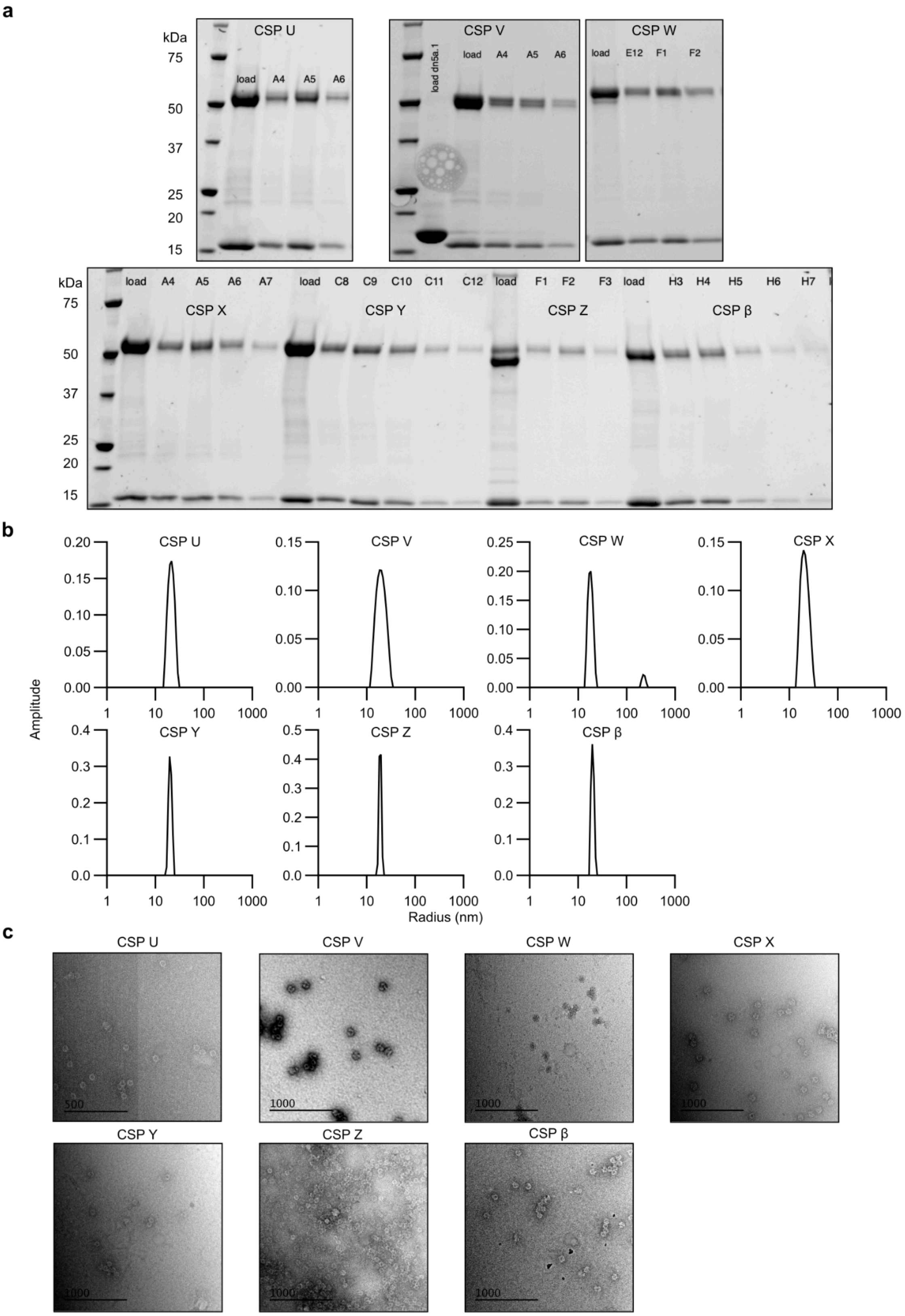

Supplementary Figure 3. Characterization data for I53-50 nanoparticle immunogens comprising non-native CSP repeats. a SDS-PAGE, **b** DLS, and **c** nsEM of nanoparticles used for immunization in non-native CSP repeat study.

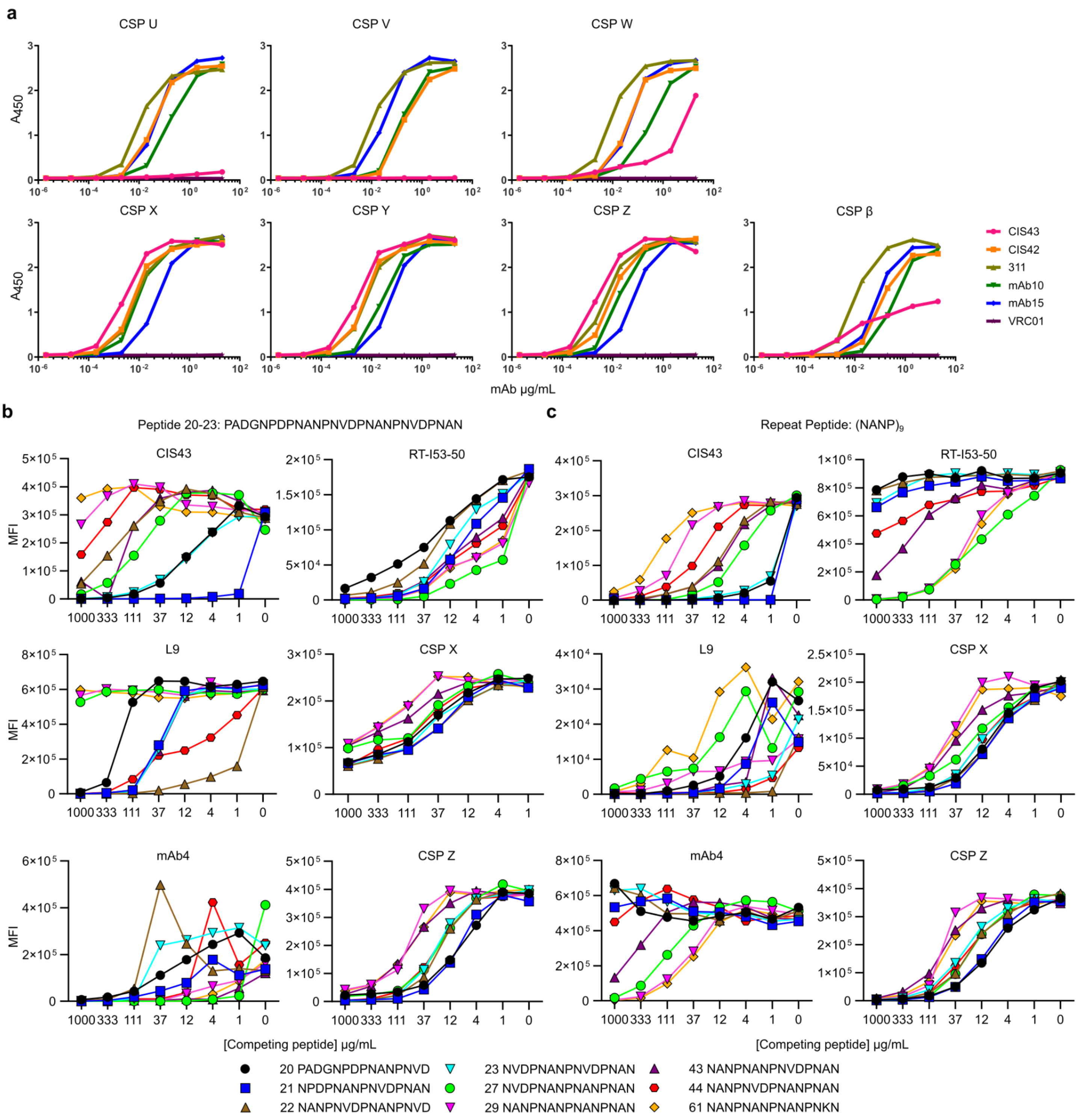

Supplementary Figure 4. Non-native CSP I53-50 nanoparticle antigenicity and epitope mapping by peptide competition assay. a Antigenicity ELISA curves for each nanoparticle immunogen against a panel of mAbs targeting the junctional epitope (CIS43 & CIS42), Region I (5D5), the major repeats (mAb10), and the C-terminal domain (mAb15). VRC01 was used as a negative control. **b** Peptide competition assay using pooled mouse sera after the 3rd immunization from each group with peptide 20-23 as the plated antigen. **c** Peptide competition assay with the repeat peptide $(NANP)_9$ as the plated antigen.

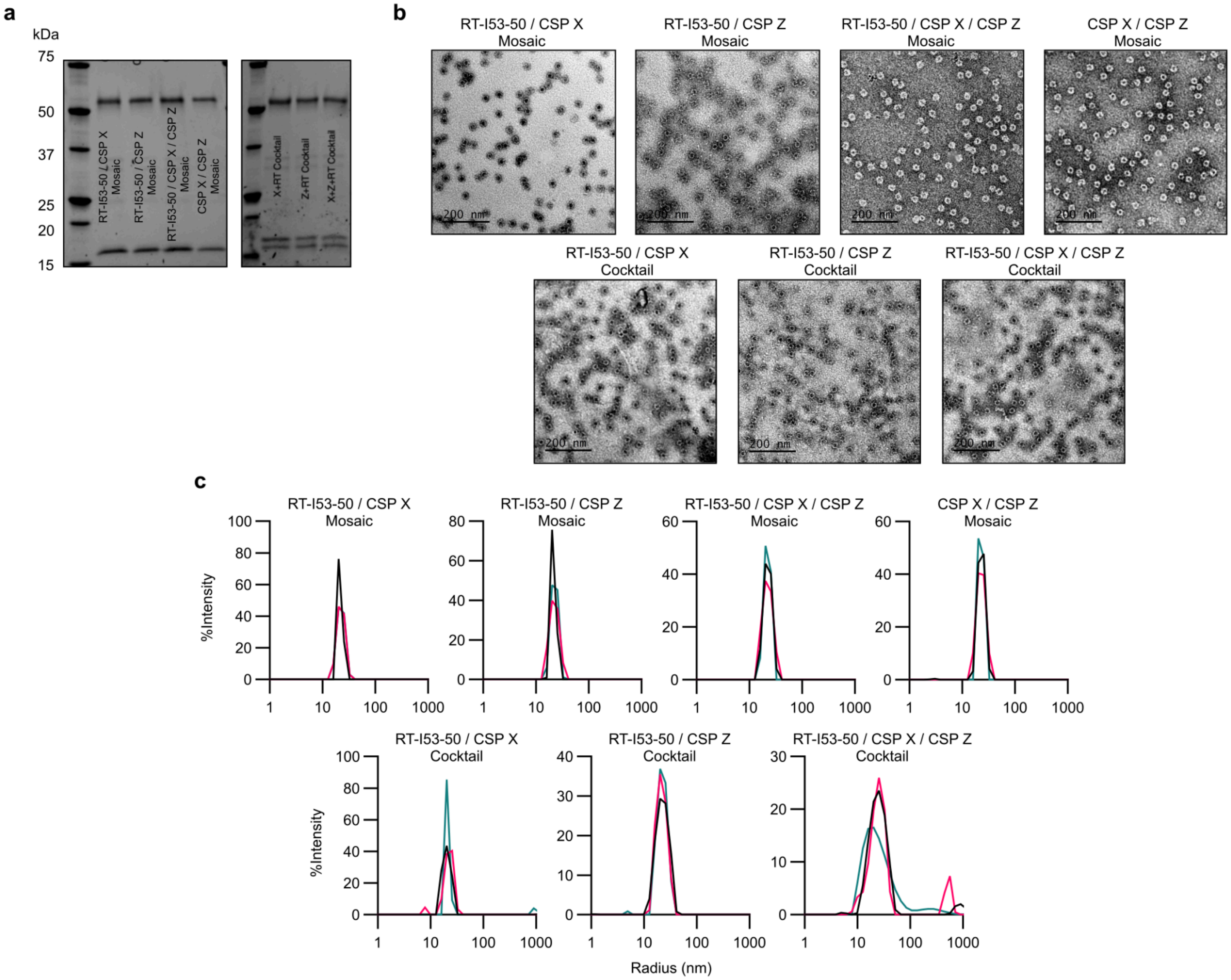

Supplementary Figure 5. Characterization data for CSP mosaic and cocktail nanoparticles. a SDS-PAGE, **b** nsEM, and **c** DLS data for cocktail and mosaic CSP-repeat nanoparticles.

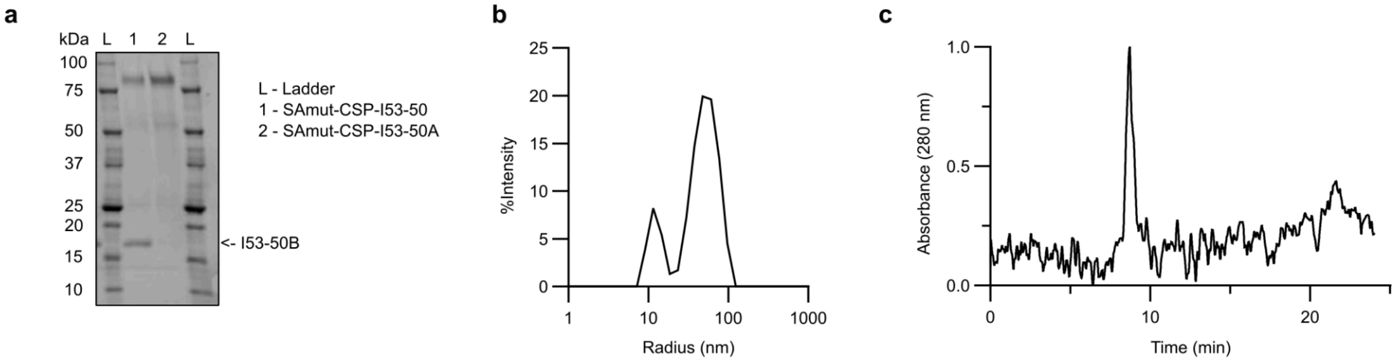

Supplementary Figure 6. Characterization data for SAmut-CSP-I53-50. a SDS-PAGE, **b** DLS, and **c** SEC chromatogram of SAmut-CSP-I53-50 nanoparticle used for immunizations in Figure 6.

Plasmid Name	Parent Scaffold	Description	Expressed?	Point of Failure	Nanoparticle Made?	Mouse Study	Sequence
**RT-I53-50**	I53-50	18 NANP repeats and C-term of CSP on I53-50A	Yes	N/A	Yes	Figure 2, 3, 4, 5	MSSNSKMDPNANPNANPNANPNAN<br>PNANPNANPNANPNANPNANPNANP<br>NANPNANPNANPNANPNANPNANPN<br>ANPNANPNKNNQGNGQGHNMPNDP<br>NRNVDENANANSAVKNNNNEEPSDK<br>HIKEYLNKIQNSLSTEWSPCSVTCGN<br>GIQVRIKPGSANKPKDELDYANDIEKK<br>ICKMEKCSSVRTMKMEELFKKHKIVA<br>VLRANSVEEAIEKAVAVFAGGVHLIEIT<br>FTVPDADTVIKALSVLKEKGAIIGAGT<br>VTSVEQCRKAVESGAEFIVSPHLDEEI<br>SQFCKEKGVFYMPGVMTPTELVKAM<br>KLGHTILKLFPGEVVGPQFVKAMKGP<br>FPNVKFVPTGGVNLDNVCEWFKAGV<br>LAVGVGSALVKGTPDEVREKAKAFVE<br>KIRGCTELEHHHHHH
**CSP-A**	I53-50	N-term + R1 + Natural Repeats + 7 NANPs on I53-50A	Yes	Aggregates	No	N/A	MQEYQSYGSSSNTRVLNELNYDNAG<br>TNLYNELEMNYYGKQENWYSLKKNS<br>RSLGENDDGNNEDNEKLRKPKHKKL<br>KQPADGNPDPNANPNVDPNANPNVD<br>PNANPNVDPNANPNANPNANPNANP<br>NANPNANPEKAAKAEEAARKMEELF<br>KKHKIVAVLRANSVEEAIEKAVAVFAG<br>GVHLIEITFTVPDADTVIKALSVLKEKG<br>AIIGAGTVTSVEQCRKAVESGAEFIVS<br>PHLDEEISQFCKEKGVFYMPGVMTPT<br>ELVKAMKLGHDILKLFPGEVVGPQFV<br>KAMKGPFFPNVKFVPTGGVNLDNVCE<br>WFKAGVLAVGVGDALVKGDPDEVRE<br>KAKKFVEKIRGCTELEHHHHHH
**CSP-B**	I53-50	N-term + R1 + Natural Repeats - NPDP + 7 NANPs on I53-50A	Yes	Aggregates	No	N/A	MQEYQSYGSSSNTRVLNELNYDNAG<br>TNLYNELEMNYYGKQENWYSLKKNS<br>RSLGENDDGNNEDNEKLRKPKHKKL<br>KQPADGNANPNANPNVDPNANPNVD<br>PNANPNVDPNANPNANPNANPNANP<br>NANPNANPEKAAKAEEAARKMEELF<br>KKHKIVAVLRANSVEEAIEKAVAVFAG<br>GVHLIEITFTVPDADTVIKALSVLKEKG<br>AIIGAGTVTSVEQCRKAVESGAEFIVS<br>PHLDEEISQFCKEKGVFYMPGVMTPT<br>ELVKAMKLGHDILKLFPGEVVGPQFV<br>KAMKGPFFPNVKFVPTGGVNLDNVCE<br>WFKAGVLAVGVGDALVKGDPDEVRE<br>KAKKFVEKIRGCTELEHHHHHH
**CSP-C**	I53-50	N term + R1 + NPDP on I53-50A	Yes	Aggregates	No	N/A	MQEYQSYGSSSNTRVLNELNYDNAG<br>TNLYNELEMNYYGKQENWYSLKKNS<br>RSLGENDDGNNEDNEKLRKPKHKKL<br>KQPADGNPDPNANPNVDPNANEKAA<br>KAEEAARKMEELFKKHKIVAVLRANS<br>VEEAIEKAVAVFAGGVHLIEITFTVPDA<br>DTVIKALSVLKEKGAIIGAGTVTSVEQ<br>CRKAVESGAEFIVSPHLDEEISQFCKE<br>KGVFYMPGVMTPTELVKAMKLGHDIL<br>KLFPGEVVGPQFVKAMKGPFFPNVKF<br>VPTGGVNLDNVCEWFKAGVLAVGVG<br>DALVKGDPDEVREKAKKFVEKIRGCT<br>ELEHHHHHH
**CSP-D**	I53-50	N-term + R1 + Natural Repeats on I53-50A	Yes	Aggregates	No	N/A	MQEYQSYGSSSNTRVLNELNYDNAG<br>TNLYNELEMNYYGKQENWYSLKKNS<br>RSLGENDDGNNEDNEKLRKPKHKKL<br>KQPADGNPDPNANPNVDPNANPNVD<br>PNANPNVDPNANEKAAKAEEAARKM<br>EELFKKHKIVAVLRANSVEEAIEKAVAV<br>FAGGVHLIEITFTVPDADTVIKALSVLK<br>EKGAIIGAGTVTSVEQCRKAVESGAE<br>FIVSPHLDEEISQFCKEKGVFYMPGV<br>MTPTELVKAMKLGHDILKLFPGEVVG<br>PQFVKAMKGPFFPNVKFVPTGGVNLD<br>NVCEWFKAGVLAVGVGDALVKGDPD<br>EVREKAKKFVEKIRGCTELEHHHHHH
**CSP-E**	I53-50	N-term + R1 + Natural Repeats - NPDP + 3 NANPs on I53-50A	Yes	Aggregates	No	N/A	MQEYQSYGSSSNTRVLNELNYDNAG<br>TNLYNELEMNYYGKQENWYSLKKNS<br>RSLGENDDGNNEDNEKLRKPKHKKL<br>KQPADGNPDPNANPNVDPNANPNVD<br>PNANPNVDPNANPNANPNANPNANE<br>KAAKAEEAARKMEELFKKHKIVAVLR<br>ANSVEEAIEKAVAVFAGGVHLIEITFTV<br>PDADTVIKALSVLKEKGAIIGAGTVTS<br>VEQCRKAVESGAEFIVSPHLDEEISQF<br>CKEKGVFYMPGVMTPTELVKAMKLG<br>HDILKLFPGEVVGPQFVKAMKGPFPN<br>VKFVPTGGVNLDNVCEWFKAGVLAV<br>GVGDALVKGDPDEVREKAKKFVEKIR<br>GCTELEHHHHHH

**CSP-F**	I53-50	R1 + Natural Repeats + NANP, NVDP repeats on I53-50A	Yes	N/A	Yes	Figure 2	MSLGENDDGNNEDNEKLRKPKHKKL<br>KQPADGNPDPNANPNVDPNANPNVD<br>PNANPNVDPNANPNANPNANPNANP<br>NANPNANPNANPNANPNANPNANPN<br>ANPNANPNANPNANPNANPNANPNA<br>NPNVDPNANPNANPNANPEKAAKAE<br>EAARKMEELFKKHKIVAVLRANSVEE<br>AIEKAVAVFAGGVHLIEITFTVPDADTV<br>IKALSVLKEKGAIIGAGTVTSVEQCRK<br>AVESGAEFIVSPHLDEEISQFCKEKGV<br>FYMPGVMTPTELVKAMKLGHDILKLF<br>PGEVVGPQFVKAMKGPFPNVKFVPT<br>GGVNLDNVCEWFKAGVLAVGVGDAL<br>VKGDPDEVREKAKKFVEKIRGCTELE<br>HHHHHH
**CSP-G**	I53-50	CSP F + N-term on I53-50A	Yes	Aggregates	No	N/A	MQEYQSYGSSSNTRVLNELNYDNAG<br>TNLYNELEMNYYGKQENWYSLKKNS<br>RSLGENDDGNNEDNEKLRKPKHKKL<br>KQPADGNPDPNANPNVDPNANPNVD<br>PNANPNVDPNANPNANPNANPNANP<br>NANPNANPNANPNANPNANPNANPN<br>ANPNANPNANPNANPNANPNANPNA<br>NPNVDPNANPNANPNANPEKAAKAE<br>EAARKMEELFKKHKIVAVLRANSVEE<br>AIEKAVAVFAGGVHLIEITFTVPDADTV<br>IKALSVLKEKGAIIGAGTVTSVEQCRK<br>AVESGAEFIVSPHLDEEISQFCKEKGV<br>FYMPGVMTPTELVKAMKLGHDILKLF<br>PGEVVGPQFVKAMKGPFPNVKFVPT<br>GGVNLDNVCEWFKAGVLAVGVGDAL<br>VKGDPDEVREKAKKFVEKIRGCTELE<br>HHHHHH
**CSP-H**	I53-50	N-term + R1 + NPDP and NVDP repeat with 15 NANP on I53-50A	Yes	Aggregates	No	N/A	MQEYQSYGSSSNTRVLNELNYDNAG<br>TNLYNELEMNYYGKQENWYSLKKNS<br>RSLGENDDGNNEDNEKLRKPKHKKL<br>KQPADGNPDPNANPNVDPNANPNAN<br>PNANPNANPNANPNANPNANPNANP<br>NANPNANPNANPNANPNANPNANPN<br>ANPNANPNANPNANPNANPNANPNA<br>NPEKAAKAEEAARKMEELFKKHKIVA<br>VLRANSVEEAIEKAVAVFAGGVHLIEIT<br>FTVPDADTVIKALSVLKEKGAIIGAGT<br>VTSVEQCRKAVESGAEFIVSPHLDEEI<br>SQFCKEKGVFYMPGVMTPTELVKAM<br>KLGHDILKLFPGEVVGPQFVKAMKGP<br>FPNVKFVPTGGVNLDNVCEWFKAGV<br>LAVGVGDALVKGDPDEVREKAKKFVE<br>KIRGCTELEHHHHHH
**CSP-A2**	I53-50	CSP-A without N-term on I53-50A	Yes	Aggregates	No	N/A	MSLGENDDGNNEDNEKLRKPKHKKL<br>KQPADGNPDPNANPNVDPNANPNVD<br>PNANLNVDPNANPNANPNANPNANP<br>NANPNANPEKAAKAEEAARKMEELF<br>KKHKIVAVLRANSVEEAIEKAVAVFAG<br>GVHLIEITFTVPDADTVIKALSVLKEKG<br>AIIGAGTVTSVEQCRKAVESGAEFIVS<br>PHLDEEISQFCKEKGVFYMPGVMTPT<br>ELVKAMKLGHDILKLFPGEVVGPQFV<br>KAMKGPFPNVKFVPTGGVNLDNVCE<br>WFKAGVLAVGVGDALVKGDPDEVRE<br>KAKKFVEKIRGCTELEHHHHHH
**CSP-B2**	I53-50	CSP-B without N-term on I53-50A	Yes	Aggregates	No	N/A	MSLGENDDGNNEDNEKLRKPKHKKL<br>KQPADGNANPNANPNVDPNANPNVD<br>PNANPNVDPNANPNANPNANPNANP<br>NANPNANPEKAAKAEEAARKMEELF<br>KKHKIVAVLRANSVEEAIEKAVAVFAG<br>GVHLIEITFTVPDADTVIKALSVLKEKG<br>AIIGAGTVTSVEQCRKAVESGAEFIVS<br>PHLDEEISQFCKEKGVFYMPGVMTPT<br>ELVKAMKLGHDILKLFPGEVVGPQFV<br>KAMKGPFPNVKFVPTGGVNLDNVCE<br>WFKAGVLAVGVGDALVKGDPDEVRE<br>KAKKFVEKIRGCTELEHHHHHH
**CSP-C2**	I53-50	CSP-C without N-term on I53-50A	Yes	Aggregates	No	N/A	MSLGENDDGNNEDNEKLRKPKHKKL<br>KQPADGNPDPNANPNVDPNANEKAA<br>KAEEAARKMEELFKKHKIVAVLRANS<br>VEEAIEKAVAVFAGGVHLIEITFTVPDA<br>DTVIKALSVLKEKGAIIGAGTVTSVEQ<br>CRKAVESGAEFIVSPHLDEEISQFCKE<br>KGVFYMPGVMTPTELVKAMKLGHDIL<br>KLFPGEVVGPQFVKAMKGPFPNVKF<br>VPTGGVNLDNVCEWFKAGVLAVGVG<br>DALVKGDPDEVREKAKKFVEKIRGCT<br>ELEHHHHHH

**CSP-D2**	I53-50	CSP-D without N-term on It 53-50	Yes	Aggregates	No	N/A	MSLGENDDGNNEDNEKLRKPKHKKL<br>KQPADGNPDPNANPNVDPNANPNVD<br>PNANPNVDPNANEKAAKAEEAARKM<br>EELFKKHKIVAVLRANSVEEAIEKAVAV<br>FAGGVHLIEITFTVPDADTVIKALSVLK<br>EKGAIIGAGTVTSVEQCRKAVESGAE<br>FIVSPHLDEEISQFCKEKGVFYMPGV<br>MTPTELVKAMKLGHDILKLFPGEVVG<br>PQFVKAMKGPFPNVKFVPTGGVNLD<br>NVCEWFKAGVLAVGVGDALVKGDPD<br>EVREKAKKFVEKIRGCTELEHHHHHH
**CSP-E2**	I53-50	CSP-E without N-term on I53-50A	Yes	Aggregates	No	N/A	MSLGENDDGNNEDNEKLRKPKHKKL<br>KQPADGNPDPNANPNVDPNANPNVD<br>PNANPNVDPNANPNANPNANPNANE<br>KAAKAEEAARKMEELFKKHKIVAVLR<br>ANSVEEAIEKAVAVFAGGVHLIEITFTV<br>PDADTVIKALSVLKEKGAIIGAGTVTS<br>VEQCRKAVESGAEFIVSPHLDEEISQF<br>CKEKGVFYMPGVMTPTELVKAMKLG<br>HDILKLFPGEVVGPQFVKAMKGPFPN<br>VKFVPTGGVNLDNVCEWFKAGVLAV<br>GVGDALVKGDPDEVREKAKKFVEKIR<br>GCTELEHHHHHH
**CSP-G2**	I53-50	CSP-G without N-term on I53-50A	Yes	Aggregates	No	N/A	MSLGENDDGNNEDNEKLRKPKHKKL<br>KQPADGNPDPNANPNVDPNANPNVD<br>PNANPNVDPNANPNANPNANPNANP<br>NANPNANPNANPNANPNANPNANPN<br>ANPNANPNANPNANPNANPNANPNA<br>NPNVDPNANPNANPNANPEKAAKAE<br>EAARKMEELFKKHKIVAVLRANSVEE<br>AIEKAVAVFAGGVHLIEITFTVPDADTV<br>IKALSVLKEKGAIIGAGTVTSVEQCRK<br>AVESGAEFIVSPHLDEEISQFCKEKGV<br>FYMPGVMTPTELVKAMKLGHDILKLF<br>PGEVVGPQFVKAMKGPFPNVKFVPT<br>GGVNLDNVCEWFKAGVLAVGVGDAL<br>VKGDPDEVREKAKKFVEKIRGCTELE<br>HHHHHH
**CSP-H2**	I53-50	CSP-H without N-term on I53-50A	Yes	Aggregates	No	N/A	MSLGENDDGNNEDNEKLRKPKHKKL<br>KQPADGNPDPNANPNVDPNANPNAN<br>PNANPNANPNANPNANPNANPNANP<br>NANPNANPNANPNANPNANPNANPN<br>ANPNANPNANPNANPNANPNANPNA<br>NPEKAAKAEEAARKMEELFKKHKIVA<br>VLRANSVEEAIEKAVAVFAGGVHLIEIT<br>FTVPDADTVIKALSVLKEKGAIIGAGT<br>VTSVEQCRKAVESGAEFIVSPHLDEEI<br>SQFCKEKGVFYMPGVMTPTELVKAM<br>KLGHDILKLFPGEVVGPQFVKAMKGP<br>FPNVKFVPTGGVNLDNVCEWFKAGV<br>LAVGVGDALVKGDPDEVREKAKKFVE<br>KIRGCTELEHHHHHH
**CSP-J**	I53-50	R1 + Natural Repeats + 17 NANPs on I53-50A	Yes	N/A	Yes	Figure 2	MSLGENDDGNNEDNEKLRKPKHKKL<br>KQPADGNPDPNANPNVDPNANPNVD<br>PNANPNVDPNANPNANPNANPNANP<br>NANPNANPNANPNANPNANPNANPN<br>ANPNANPNANPNANPNANPNANPNA<br>NPNANPNANPNKNNQGNGQGHNMP<br>NDPNRNVDENANANSAVKNNNNEEP<br>SDKHIKEYLNKIQNSLSTEWSPCSVT<br>CGNGIQVRIKPGSANKPKDELDYAND<br>IEKKICKMEKCSSVRTMKMEELFKKH<br>KIVAVLRANSVEEAIEKAVAVFAGGVH<br>LIEITFTVPDADTVIKALSVLKEKGAIIG<br>AGTVTSVEQCRKAVESGAEFIVSPHL<br>DEEISQFCKEKGVFYMPGVMTPTELV<br>KAMKLGHTILKLFPGEVVGPQFVKAM<br>KGPFPNVKFVPTGGVNLDNVCEWFK<br>AGVLAVGVGSALVKGTPDEVREKAKA<br>FVEKIRGCTELEHHHHHH
**CSP-K**	I53-50	Natural Repeats + 17 NANPS on I53-50A	Yes	N/A	Yes	Figure 2	MSSNSKMDPNPDPNANPNVDPNAN<br>PNVDPNANPNVDPNANPNANPNANP<br>NANPNANPNANPNANPNANPNANPN<br>ANPNANPNANPNANPNANPNANPNA<br>NPNANPNANPNANPNKNNQGNGQG<br>HNMPNDPNRNVDENANANSAVKNN<br>NNEEPSDKHIKEYLNKIQNSLSTEWS<br>PCSVTCGNGIQVRIKPGSANKPKDEL<br>DYANDIEKKICKMEKCSSVRTMKMEE<br>LFKKHKIVAVLRANSVEEAIEKAVAVFA<br>GGVHLIEITFTVPDADTVIKALSVLKEK<br>GAIIGAGTVTSVEQCRKAVESGAEFIV<br>SPHLDEEISQFCKEKGVFYMPGVMT<br>PTELVKAMKLGHTILKLFPGEVVGPQ<br>FVKAMKGPFPNVKFVPTGGVNLDNV<br>CEWFKAGVLAVGVGSALVKGTPDEV<br>REKAKAFVEKIRGCTELEHHHHHH

**CSP-L**	I53-50	Full Set of repeats on I53-50A	No	Expression	No	N/A	MSSNSKMDPNPDPNANPNVDPNANPNVDPNANPNVDPNANPNANPNANPNANPNANPNANPNANPNANPNANPNANPNANPNANPNANPNANPNANPNANPNVDPNANPNANPNANPNANPNANPNANPNANPNANPNANPNANPNANPNANPNANPNANPNANPNANPNANPNANPNANPNKNNQGNGQGHNMPNDPNRNVDENANANSAVKNNNNEEPSDKHIKEYLNKIQNSLSTEWSPCSVTCGNGIQVRIKPGSANKPKDELDYANDIEKKICKMEKCSSVRTMKMEELFKKHKIVAVLRANSVEEAIEKAVAVFAGGVHLIEITFTVPDADTVIKALSVLKEKGAIIGAGTVTSVEQCRKAVESGAEFIVSPHLDEEISQFCKEKGVFYMPGVMTPTELVKAMKLGHDILKLFPGEVVGPQFVKAMKGPFPNVKFVPTGGVNLDNVCEWFKAGVLAVGVGDALVKGDPDEVREKAKKFVEKIRGCTELEHHHHHH
**CSP-M**	I53-50	R1 + Full set of repeats on I53-50A	No	Expression	No	N/A	MSLGENDDGNNEDNEKLRKPKHKKLKQPADGNPDPNANPNVDPNANPNVDPNANPNVDPNANPNANPNANPNANPNANPNANPNANPNANPNANPNANPNANPNANPNANPNANPNANPNANPNANPNVDPNANPNANPNANPNANPNANPNANPNANPNANPNANPNANPNANPNANPNANPNANPNANPNANPNKNNQGNGQGHNMPNDPNRNVDENANANSAVKNNNNEEPSDKHIKEYLNKIQNSLSTEWSPCSVTCGNGIQVRIKPGSANKPKDELDYANDIEKKICKMEKCSSVRTMKMEELFKKHKIVAVLRANSVEEAIEKAVAVFAGGVHLIEITFTVPDADTVIKALSVLKEKGAIIGAGTVTSVEQCRKAVESGAEFIVSPHLDEEISQFCKEKGVFYMPGVMTPTELVKAMKLGHDILKLFPGEVVGPQFVKAMKGPFPNVKFVPTGGVNLDNVCEWFKAGVLAVGVGDALVKGDPDEVREKAKKFVEKIRGCTELEHHHHHH
**CSP-P**	I53-50	R1 + natural Repeats + C term on I53-50A	Yes	N/A	Yes	Figure 2	MSLGENDDGNNEDNEKLRKPKHKKLKQPADGNPDPNANPNVDPNANPNVDPNANPNVDPNANPNKNNQGNGQGHNMPNDPNRNVDENANANSAVKNNNNEEPSDKHIKEYLNKIQNSLSTEWSPCSVTCGNGIQVRIKPGSANKPKDELDYANDIEKKICKMEKCSSVRTMKMEELFKKHKIVAVLRANSVEEAIEKAVAVFAGGVHLIEITFTVPDADTVIKALSVLKEKGAIIGAGTVTSVEQCRKAVESGAEFIVSPHLDEEISQFCKEKGVFYMPGVMTPTELVKAMKLGHTILKLFPGEVVGPQFVKAMKGPFPNVKFVPTGGVNLDNVCEWFKAGVLAVGVGSALVKGTPDEVREKAKAFVEKIRGCTELEHHHHHH
**CSP-Q**	I53-50	R1 + natural Repeats + 3 NANP + C term on I53-50A	Yes	N/A	Yes	Figure 2	MSLGENDDGNNEDNEKLRKPKHKKLKQPADGNPDPNANPNVDPNANPNVDPNANPNVDPNANPNANPNANPNKNNQGNGQGHNMPNDPNRNVDENANANSAVKNNNNEEPSDKHIKEYLNKIQNSLSTEWSPCSVTCGNGIQVRIKPGSANKPKDELDYANDIEKKICKMEKCSSVRTMKMEELFKKHKIVAVLRANSVEEAIEKAVAVFAGGVHLIEITFTVPDADTVIKALSVLKEKGAIIGAGTVTSVEQCRKAVESGAEFIVSPHLDEEISQFCKEKGVFYMPGVMTPTELVKAMKLGHTILKLFPGEVVGPQFVKAMKGPFPNVKFVPTGGVNLDNVCEWFKAGVLAVGVGSALVKGTPDEVREKAKAFVEKIRGCTELEHHHHHH
CSP-R	I53-50	R1 + natural Repeats + 6 NANP + C term on I53-50A	Yes	N/A	Yes	Figure 2	MSLGENDDGNNEDNEKLRKPKHKKLKQPADGNPDPNANPNVDPNANPNVDPNANPNVDPNANPNANPNANPNANPNANPNANPNKNNQGNGQGHNMPNDPNRNVDENANANSAVKNNNNEEPSDKHIKEYLNKIQNSLSTEWSPCSVTCGNGIQVRIKPGSANKPKDELDYANDIEKKICKMEKCSSVRTMKMEELFKKHKIVAVLRANSVEEAIEKAVAVFAGGVHLIEITFTVPDADTVIKALSVLKEKGAIIGAGTVTSVEQCRKAVESGAEFIVSPHLDEEISQFCKEKGVFYMPGVMTPTELVKAMKLGHTILKLFPGEVVGPQFVKAMKGPFPNVKFVPTGGVNLDNVCEWFKAGVLAVGVGSALVKGTPDEVREKAKAFVEKIRGCTELEHHHHHH
CSP-J2	I53-dn5	CSP-J C-terminal Fusion to I53-dn5B	Yes	Degrades	No	N/A	

CSP-K2	I53-dn5	CSP-K C-terminal Fusion to I53-dn5B	Yes	Degrades	No	N/A	
CSP-L2	I53-dn5	CSP-L C-terminal Fusion to I53-dn5B	Yes	Degrades	No	N/A	
CSP-M2	I53-dn5	CSP-M C-terminal Fusion to I53-dn5B	Yes	Degrades	No	N/A	
CSP-P2	I53-dn5	CSP-P C-terminal Fusion to I53-dn5B	Yes	Degrades	No	N/A	
CSP-Q2	I53-dn5	CSP-Q C-terminal Fusion to I53-dn5B	Yes	Degrades	No	N/A	
CSP-R2	I53-dn5	CSP-R C-terminal Fusion to I53-dn5B	Yes	Degrades	No	N/A	
CSP-J3	I53-dn5	CSP-J N-terminal Fusion to I53-dn5B	Yes	Degrades	No	N/A	MSSNSKMDPLGENDDGNNEDNEKLRKPKHKKLKQPADGNPDPNANPNVDPNANPNVDPNANPNVDPNANPNANPNANPNANPNANPNANPNANPNANPNANPNANPNANPNANPNANPNANPNANPNANPNANPNANPNANPNKNNQGNGQGHNMPNDPNRNVDENANANSAVKNNNNEEPSDKHIKEYLNKIQNSLSTEWSPCSVTCGNGIQVRIKPGSANKPKDELDYANDIEKKICKMEKCSSVRTMLEGGSGGDGGSGGDEEAELAYLLGELAYKLGEYRIAIRAYRIALKRDPNNAEAWYNLGNAYYKQGRYREAIEYYQKALELDPNNAEAWYNLGNAYYERGEYEEAIEYYRKALRLDPNNADAMQNLLNAKMREEHHHHHH
CSP-K3	I53-dn5	CSP-K N-terminal Fusion to I53-dn5B	Yes	Degrades	No	N/A	MSSNSKMDPNPDPNANPNVDPNANPNVDPNANPNVDPNANPNANPNANPNANPNANPNANPNANPNANPNANPNANPNANPNANPNANPNANPNANPNANPNANPNANPNKNNQGNGQGHNMPNDPNRNVDENANANSAVKNNNNEEPSDKHIKEYLNKIQNSLSTEWSPCSVTCGNGIQVRIKPGSANKPKDELDYANDIEKKICKMEKCSSVRTMLEGGSGGDGGSGGDEEAELAYLLGELAYKLGEYRIAIRAYRIALKRDPNNAEAWYNLGNAYYKQGRYREAIEYYQKALELDPNNAEAWYNLGNAYYERGEYEEAIEYYRKALRLDPNNADAMQNLLNAKMREEHHHHHH
CSP-L3	I53-dn5	CSP-L N-terminal Fusion to I53-dn5B	Yes	Degrades	No	N/A	MSSNSKMDPNPDPNANPNVDPNANPNVDPNANPNVDPNANPNANPNANPNANPNANPNANPNANPNANPNANPNANPNANPNANPNANPNANPNANPNANPNVDPNANPNANPNANPNANPNANPNANPNANPNANPNANPNANPNANPNANPNANPNANPNANPNANPNANPNKNNQGNGQGHNMPNDPNRNVDENANANSAVKNNNNEEPSDKHIKEYLNKIQNSLSTEWSPCSVTCGNGIQVRIKPGSANKPKDELDYANDIEKKICKMEKCSSVRTMLEGGSGGDGGSGGDEEAELAYLLGELAYKLGEYRIAIRAYRIALKRDPNNAEAWYNLGNAYYKQGRYREAIEYYQKALELDPNNAEAWYNLGNAYYERGEYEEAIEYYRKALRLDPNNADAMQNLLNAKMREEHHHHHH
CSP-M3	I53-dn5	CSP-M N-terminal Fusion to I53-dn5B	Yes	Degrades	No	N/A	MSSNSKMDPSLGENDDGNNEDNEKLRKPKHKKLKQPADGNPDPNANPNVDPNANPNVDPNANPNVDPNANPNANPNANPNANPNANPNANPNANPNANPNANPNANPNANPNANPNANPNANPNANPNANPNANPNVDPNANPNANPNANPNANPNANPNANPNANPNANPNANPNANPNANPNANPNANPNANPNANPNANPNANPNANPNKNNQGNGQGHNMPNDPNRNVDENANANSAVKNNNNEEPSDKHIKEYLNKIQNSLSTEWSPCSVTCGNGIQVRIKPGSANKPKDELDYANDIEKKICKMEKCSSVRTMLEGGSGGDGGSGGDEEAELAYLLGELAYKLGEYRIAIRAYRIALKRDPNNAEAWYNLGNAYYKQGRYREAIEYYQKALELDPNNAEAWYNLGNAYYERGEYEEAIEYYRKALRLDPNNADAMQNLLNAKMREEHHHHHH

CSP-P3	I53-dn5	CSP-P N-terminal Fusion to I53-dn5B	Yes	Degrades	No	N/A	MSSNSKMDPSLGENDDGNNEDNEKLRKPKHKKLKQPADGNPDPNANPNVDPNANPNVDPNANPNVDPNANPNKNNQGNGQGHNMPNDPNRNVDENANANSAVKNNNNEEPSDKHIKEYLNKIQNSLSTEWSPCSVTCGNGIQVRIKPGSANKPKDELDYANDIEKKICKMEKCSSVRTMLEGGSGGDGGSGGDEEAELAYLLGELAYKLGEYRIAIRAYRIALKRDPNNAEAWYNLGNAYYKQGRYREAIEYYQKALELDPNNAEAWYNLGNAYYERGEYEEAIEYYRKALRLDPNNADAMQNLLNAKMREEHHHHHH
CSP-Q3	I53-dn5	CSP-Q N-terminal Fusion to I53-dn5B	Yes	Degrades	No	N/A	MSSNSKMDPSLGENDDGNNEDNEKLRKPKHKKLKQPADGNPDPNANPNVDPNANPNVDPNANPNVDPNANPNANPNANPNANPNKNNQGNGQGHNMPNDPNRNVDENANANSAVKNNNNEEPSDKHIKEYLNKIQNSLSTEWSPCSVTCGNGIQVRIKPGSANKPKDELDYANDIEKKICKMEKCSSVRTMLEGGSGGDGGSGGDEEAELAYLLGELAYKLGEYRIAIRAYRIALKRDPNNAEAWYNLGNAYYKQGRYREAIEYYQKALELDPNNAEAWYNLGNAYYERGEYEEAIEYYRKALRLDPNNADAMQNLLNAKMREEHHHHHH
CSP-R3	I53-dn5	CSP-R N-terminal Fusion to I53-dn5B	Yes	Degrades	No	N/A	MSSNSKMDPSLGENDDGNNEDNEKLRKPKHKKLKQPADGNPDPNANPNVDPNANPNVDPNANPNVDPNANPNANPNANPNANPNANPNANPNKNNQGNGQGHNMPNDPNRNVDENANANSAVKNNNNEEPSDKHIKEYLNKIQNSLSTEWSPCSVTCGNGIQVRIKPGSANKPKDELDYANDIEKKICKMEKCSSVRTMLEGGSGGDGGSGGDEEAELAYLLGELAYKLGEYRIAIRAYRIALKRDPNNAEAWYNLGNAYYKQGRYREAIEYYQKALELDPNNAEAWYNLGNAYYERGEYEEAIEYYRKALRLDPNNADAMQNLLNAKMREEHHHHHH
CSP-S	I53-50	C-term on I53-50A	Yes	N/A	Yes	Not used	MNKNNQGNGQGHNMPNDPNRNVDENANANSAVKNNNNEEPSDKHIKEYLNKIQNSLSTEWSPCSVTCGNGIQVRIKPGSANKPKDELDYANDIEKKICKMEKCSSVRTMKMEELFKKHKIVAVLRANSVEEAIEKAVAVFAGGVHLIEITFTVPDADTVIKALSVLKEKGAIIGAGTVTSVEQCRKAVESGAEFIVSPHLDEEISQFCKEKGVFYMPGVMTPTELVKAMKLGHTILKLFPGEVVGPQFVKAMKGPFPNVKFVPTGGVNLDNVCEWFKAGVLAVGVGSALVKGTPDEVREKAKAFVEKIRGCTEGSHHHHHH
CSP-T	I3-01	18 NANP repeats and C-term on I301	No	Expression	No	N/A	MSSNSKMDPNANPNANPNANPNANPNANPNANPNANPNANPNANPNANPNANPNANPNANPNANPNANPNANPNANPNANPNKNNQGNGQGHNMPNDPNRNVDENANANSAVKNNNNEEPSDKHIKEYLNKIQNSLSTEWSPCSVTCGNGIQVRIKPGSANKPKDELDYANDIEKKICKMEKCSSVRTKMEELFKKHKIVAVLRANSVEEAKKKALAVFLGGVDLIEITFTVPDADTVIKELSFLKEMGAIIGAGTVTSVEQCRKAVESGAEFIVSPHLDEEISQFCKEKGVFYMPGVMTPTELVKAMKLGHTILKLFPGEVVGPQFVKAMKGPFPNVKFVPTGGVNLDNVCEWFKAGVQAVGVGSALVKGTPVEVAEKAKAFVEKIRGCTEGSHHHHHH
CSP-T2	Supercharged I3-01	18 NANP repeats and C-term on I301 supercharged	No	Expression	No	N/A	MSSNSKMDPNANPNANPNANPNANPNANPNANPNANPNANPNANPNANPNANPNANPNANPNANPNANPNANPNANPNANPNKNNQGNGQGHNMPNDPNRNVDENANANSAVKNNNNEEPSDKHIKEYLNKIQNSLSTEWSPCSVTCGNGIQVRIKPGSANKPKDELDYANDIEKKICKMEKCSSVRTMEELFKEHKIVAVLRANSVEEAKKKALAVFLGGVDLIEITFTVPDADTVIKELSFLKEMGAIIGAGTVTSVEQAREAVESGAEFIVSPHLDEEISQFAKEEGVFYMPGVMTPTELVKAMKLGHTILKLFPGEVVGPQFVEAMKGPFPNVKFVPTGGVNLDNVAEWFEAGVQAVGVGSALVEGTPVEVAEKAKAFVEKIEAATGSHHHHHH

**CSP-U**	I53-50	17 NPDP repeats + NANP + C-term on I53-50A	Yes	N/A	Yes	Figure 3	MSSNSKMDPNPDPNPDPNPDPNPD<br>PNPDPNPDPNPDPNPDPNPDPNPDP<br>NPDPNPDPNPDPNPDPNPDPNPDPN<br>PDPNANPNKNNQGNGQGHNMPNDP<br>NRNVDENANANSAVKNNNNEEPSDK<br>HIKEYLNKIQNSLSTEWSPCSVTCGN<br>GIQVRIKPGSANKPKDELDYANDIEKK<br>ICKMEKCSSVRTKMEELFKKHKIVAVL<br>RANSVEEAIEKAVAVFAGGVHLIEITFT<br>VPDADTVIKALSVLKEKGAIIGAGTVT<br>SVEQCRKAVESGAEFIVSPHLDEEIS<br>QFCKEKGVFYMPGVMTPTELVKAMK<br>LGHTILKLFPGEVVGPQFVKAMKGPF<br>PNVKFVPTGGVNLDNVCEWFKAGVL<br>AVGVGSALVKGTPDEVREKAKAFVEK<br>IRGCTEGSHHHHHH
**CSP-V**	I53-50	17 NVDP repeats + NANP + C-term on I53-50A	Yes	N/A	Yes	Figure 3	MSSNSKMDPNVDPNVDPNVDPNVD<br>PNVDPNVDPNVDPNVDPNVDPNVDP<br>NVDPNVDPNVDPNVDPNVDPNVDPN<br>VDPNANPNKNNQGNGQGHNMPNDP<br>NRNVDENANANSAVKNNNNEEPSDK<br>HIKEYLNKIQNSLSTEWSPCSVTCGN<br>GIQVRIKPGSANKPKDELDYANDIEKK<br>ICKMEKCSSVRTKMEELFKKHKIVAVL<br>RANSVEEAIEKAVAVFAGGVHLIEITFT<br>VPDADTVIKALSVLKEKGAIIGAGTVT<br>SVEQCRKAVESGAEFIVSPHLDEEIS<br>QFCKEKGVFYMPGVMTPTELVKAMK<br>LGHTILKLFPGEVVGPQFVKAMKGPF<br>PNVKFVPTGGVNLDNVCEWFKAGVL<br>AVGVGSALVKGTPDEVREKAKAFVEK<br>IRGCTEGSHHHHHH
**CSP-W**	I53-50	9 sets of alternating NPDP + NANP repeats + C-term on I53-50A	Yes	N/A	Yes	Figure 3	MSSNSKMDPNPDPNANPNPDPNAN<br>PNPDPNANPNPDPNANPNPDPNANP<br>NPDPNANPNPDPNANPNPDPNANPN<br>PDPNANPNKNNQGNGQGHNMPNDP<br>NRNVDENANANSAVKNNNNEEPSDK<br>HIKEYLNKIQNSLSTEWSPCSVTCGN<br>GIQVRIKPGSANKPKDELDYANDIEKK<br>ICKMEKCSSVRTKMEELFKKHKIVAVL<br>RANSVEEAIEKAVAVFAGGVHLIEITFT<br>VPDADTVIKALSVLKEKGAIIGAGTVT<br>SVEQCRKAVESGAEFIVSPHLDEEIS<br>QFCKEKGVFYMPGVMTPTELVKAMK<br>LGHTILKLFPGEVVGPQFVKAMKGPF<br>PNVKFVPTGGVNLDNVCEWFKAGVL<br>AVGVGSALVKGTPDEVREKAKAFVEK<br>IRGCTEGSHHHHHH
**CSP-Y**	I53-50	9 sets of alternating NVDP + NANP repeats + C-term on I53-50A	Yes	N/A	Yes	Figure 3	MSSNSKMDPNPDPNANPNVDPNAN<br>PNPDPNANPNVDPNANPNPDPNANP<br>NVDPNANPNPDPNANPNVDPNANPN<br>PDPNANPNKNNQGNGQGHNMPNDP<br>NRNVDENANANSAVKNNNNEEPSDK<br>HIKEYLNKIQNSLSTEWSPCSVTCGN<br>GIQVRIKPGSANKPKDELDYANDIEKK<br>ICKMEKCSSVRTKMEELFKKHKIVAVL<br>RANSVEEAIEKAVAVFAGGVHLIEITFT<br>VPDADTVIKALSVLKEKGAIIGAGTVT<br>SVEQCRKAVESGAEFIVSPHLDEEIS<br>QFCKEKGVFYMPGVMTPTELVKAMK<br>LGHTILKLFPGEVVGPQFVKAMKGPF<br>PNVKFVPTGGVNLDNVCEWFKAGVL<br>AVGVGSALVKGTPDEVREKAKAFVEK<br>IRGCTEGSHHHHHH
**CSP-X**	I53-50	18 repeats NPDPNANPNVDPNANP + C-term on I53-50A	Yes	N/A	Yes	Figure 3	MSSNSKMDPNVDPNANPNVDPNAN<br>PNVDPNANPNVDPNANPNVDPNANP<br>NVDPNANPNVDPNANPNVDPNANPN<br>VDPNANPNKNNQGNGQGHNMPNDP<br>NRNVDENANANSAVKNNNNEEPSDK<br>HIKEYLNKIQNSLSTEWSPCSVTCGN<br>GIQVRIKPGSANKPKDELDYANDIEKK<br>ICKMEKCSSVRTKMEELFKKHKIVAVL<br>RANSVEEAIEKAVAVFAGGVHLIEITFT<br>VPDADTVIKALSVLKEKGAIIGAGTVT<br>SVEQCRKAVESGAEFIVSPHLDEEIS<br>QFCKEKGVFYMPGVMTPTELVKAMK<br>LGHTILKLFPGEVVGPQFVKAMKGPF<br>PNVKFVPTGGVNLDNVCEWFKAGVL<br>AVGVGSALVKGTPDEVREKAKAFVEK<br>IRGCTEGSHHHHHH

**CSP-Z**	I53-50	Two junctional regions and C-term on I53-50A	Yes	N/A	Yes	Figure 3	MSSNSKMDPNPDPNANPNVDPNAN<br>PNVDPNANPNVDPNANPNPDPNANP<br>NVDPNANPNVDPNANPNVDPNANPN<br>PDPNANPNKNNQGNGQGHNMPNDP<br>NRNVDENANANSAVKNNNNEEPSDK<br>HIKEYLNKIQNSLSTEWSPCSVTCGN<br>GIQVRIKPGSANKPKDELDYANDIEKK<br>ICKMEKCSSVRTKMEELFKKHKIVAVL<br>RANSVEEAIEKAVAVFAGGVHLIEITFT<br>VPDADTVIKALSVLKEKGAIIGAGTVT<br>SVEQCRKAVESGAEFIVSPHLDEEIS<br>QFCKEKGVFYMPGVMTPTELVKAMK<br>LGHTILKLFPGEVVGPQFVKAMKGPF<br>PNVKFVPTGGVNLDNVCEWFKAGVL<br>AVGVGSALVKGTPDEVREKAKAFVEK<br>IRGCTEGSHHHHHH
CSP-α	I53-50	Alternating NPDP and NVDP repeats +2XNANP + C-term on I53-50A	Yes	N/A	No	N/A	MSSNSKMDPNPDPNVDPNPDPNVD<br>PNPDPNVDPNPDPNVDPNPDPNVDP<br>NPDPNVDPNPDPNVDPNPDPNVDPN<br>PDPNANPNKNNQGNGQGHNMPNDP<br>NRNVDENANANSAVKNNNNEEPSDK<br>HIKEYLNKIQNSLSTEWSPCSVTCGN<br>GIQVRIKPGSANKPKDELDYANDIEKK<br>ICKMEKCSSVRTKMEELFKKHKIVAVL<br>RANSVEEAIEKAVAVFAGGVHLIEITFT<br>VPDADTVIKALSVLKEKGAIIGAGTVT<br>SVEQCRKAVESGAEFIVSPHLDEEIS<br>QFCKEKGVFYMPGVMTPTELVKAMK<br>LGHTILKLFPGEVVGPQFVKAMKGPF<br>PNVKFVPTGGVNLDNVCEWFKAGVL<br>AVGVGSALVKGTPDEVREKAKAFVEK<br>IRGCTEGSHHHHHH
**CSP-β**	I53-50	6 sets of NPDPNVDPNANP + C-term on I53-50A	Yes	N/A	Yes	Figure 3	MSSNSKMDPNPDPNVDPNANPNPD<br>PNVDPNANPNPDPNVDPNANPNPDP<br>NVDPNANPNPDPNVDPNANPNPDPN<br>VDPNANPNKNNQGNGQGHNMPNDP<br>NRNVDENANANSAVKNNNNEEPSDK<br>HIKEYLNKIQNSLSTEWSPCSVTCGN<br>GIQVRIKPGSANKPKDELDYANDIEKK<br>ICKMEKCSSVRTKMEELFKKHKIVAVL<br>RANSVEEAIEKAVAVFAGGVHLIEITFT<br>VPDADTVIKALSVLKEKGAIIGAGTVT<br>SVEQCRKAVESGAEFIVSPHLDEEIS<br>QFCKEKGVFYMPGVMTPTELVKAMK<br>LGHTILKLFPGEVVGPQFVKAMKGPF<br>PNVKFVPTGGVNLDNVCEWFKAGVL<br>AVGVGSALVKGTPDEVREKAKAFVEK<br>IRGCTEGSHHHHHH
CSP-γ	I53-dn5	CSP-U on I53-dn5B	Yes	Degrades	No	N/A	MSSNSKMDPNPDPNPDPNPDPNPD<br>PNPDPNPDPNPDPNPDPNPDPNPDP<br>NPDPNPDPNPDPNPDPNPDPNPDPN<br>PDPNANPNKNNQGNGQGHNMPNDP<br>NRNVDENANANSAVKNNNNEEPSDK<br>HIKEYLNKIQNSLSTEWSPCSVTCGN<br>GIQVRIKPGSANKPKDELDYANDIEKK<br>ICKMEKCSSVRTGGSGGDGGSGGD<br>EEAELAYLLGELAYKLGEYRIAIRAYRI<br>ALKRDPNNAEAWYNLGNAYYKQGRY<br>REAIEYYQKALELDPNNAEAWYNLGN<br>AYYERGEYEEAIEYYRKALRLDPNNA<br>DAMQNLLNAKMREEHHHHHH
CSP-δ	I53-dn5	CSP-V on I53-dn5B	Yes	Degrades	No	N/A	MSSNSKMDPNVDPNVDPNVDPNVD<br>PNVDPNVDPNVDPNVDPNVDPNVDP<br>NVDPNVDPNVDPNVDPNVDPNVDPN<br>VDPNANPNKNNQGNGQGHNMPNDP<br>NRNVDENANANSAVKNNNNEEPSDK<br>HIKEYLNKIQNSLSTEWSPCSVTCGN<br>GIQVRIKPGSANKPKDELDYANDIEKK<br>ICKMEKCSSVRTGGSGGDGGSGGD<br>EEAELAYLLGELAYKLGEYRIAIRAYRI<br>ALKRDPNNAEAWYNLGNAYYKQGRY<br>REAIEYYQKALELDPNNAEAWYNLGN<br>AYYERGEYEEAIEYYRKALRLDPNNA<br>DAMQNLLNAKMREEHHHHHH
CSP-ε	I53-dn5	CSP-W on I53-dn5B	Yes	Degrades	No	N/A	MSSNSKMDPNPDPNANPNPDPNAN<br>PNPDPNANPNPDPNANPNPDPNANP<br>NPDPNANPNPDPNANPNPDPNANPN<br>PDPNANPNKNNQGNGQGHNMPNDP<br>NRNVDENANANSAVKNNNNEEPSDK<br>HIKEYLNKIQNSLSTEWSPCSVTCGN<br>GIQVRIKPGSANKPKDELDYANDIEKK<br>ICKMEKCSSVRTGGSGGDGGSGGD<br>EEAELAYLLGELAYKLGEYRIAIRAYRI<br>ALKRDPNNAEAWYNLGNAYYKQGRY<br>REAIEYYQKALELDPNNAEAWYNLGN<br>AYYERGEYEEAIEYYRKALRLDPNNA<br>DAMQNLLNAKMREEHHHHHH

CSP-ζ	I53-dn5	CSP-X on I53-dn5B	Yes	Degrades	No	N/A	MSSNSKMDPNVDPNANPNVDPNANPNVDPNANPNVDPNANPNVDPNANPNVDPNANPNVDPNANPNVDPNANPNVDPNANPNKNNQGNGQGHNMPNDPNRNVDENANANSAVKNNNNEEPSDKHIKEYLNKIQNSLSTEWSPCSVTCGNGIQVRIKPGSANKPKDELDYANDIEKKICKMEKCSSVRTGGSGGDGGSGGDEEAELAYLLGELAYKLGEYRIAIRAYRIALKRDPNNAEAWYNLGNAYYKQGRYREAIEYYQKALELDPNNAEAWYNLGNAYYERGEYEEAIEYYRKALRLDPNNADAMQNLLNAKMREEHHHHHH
CSP-η	I53-dn5	CSP-Y on I53-dn5B	Yes	Degrades	No	N/A	MSSNSKMDPNPDPNANPNVDPNANPNPDPNANPNVDPNANPNPDPNANPNVDPNANPNPDPNANPNVDPNANPNPDPNANPNKNNQGNGQGHNMPNDPNRNVDENANANSAVKNNNNEEPSDKHIKEYLNKIQNSLSTEWSPCSVTCGNGIQVRIKPGSANKPKDELDYANDIEKKICKMEKCSSVRTGGSGGDGGSGGDEEAELAYLLGELAYKLGEYRIAIRAYRIALKRDPNNAEAWYNLGNAYYKQGRYREAIEYYQKALELDPNNAEAWYNLGNAYYERGEYEEAIEYYRKALRLDPNNADAMQNLLNAKMREEHHHHHH
CSP-θ	I53-dn5	CSP-Z on I53-dn5B	Yes	Degrades	No	N/A	MSSNSKMDPNPDPNANPNVDPNANPNVDPNANPNVDPNANPNPDPNANPNVDPNANPNVDPNANPNVDPNANPNPDPNANPNKNNQGNGQGHNMPNDPNRNVDENANANSAVKNNNNEEPSDKHIKEYLNKIQNSLSTEWSPCSVTCGNGIQVRIKPGSANKPKDELDYANDIEKKICKMEKCSSVRTGGSGGDGGSGGDEEAELAYLLGELAYKLGEYRIAIRAYRIALKRDPNNAEAWYNLGNAYYKQGRYREAIEYYQKALELDPNNAEAWYNLGNAYYERGEYEEAIEYYRKALRLDPNNADAMQNLLNAKMREEHHHHHH
CSP-λ	I53-dn5	CSP-α on I53-dn5B	Yes	Degrades	No	N/A	MSSNSKMDPNPDPNVDPNPDPNVDPNPDPNVDPNPDPNVDPNPDPNVDPNPDPNVDPNPDPNVDPNPDPNVDPNPDPNANPNKNNQGNGQGHNMPNDPNRNVDENANANSAVKNNNNEEPSDKHIKEYLNKIQNSLSTEWSPCSVTCGNGIQVRIKPGSANKPKDELDYANDIEKKICKMEKCSSVRTGGSGGDGGSGGDEEAELAYLLGELAYKLGEYRIAIRAYRIALKRDPNNAEAWYNLGNAYYKQGRYREAIEYYQKALELDPNNAEAWYNLGNAYYERGEYEEAIEYYRKALRLDPNNADAMQNLLNAKMREEHHHHHH
CSP-μ	I53-dn5	CSP-β on I53-dn5B	Yes	Degrades	No	N/A	MSSNSKMDPNPDPNVDPNANPNPDPNVDPNANPNPDPNVDPNANPNPDPNVDPNANPNPDPNVDPNANPNPDPNVDPNANPNKNNQGNGQGHNMPNDPNRNVDENANANSAVKNNNNEEPSDKHIKEYLNKIQNSLSTEWSPCSVTCGNGIQVRIKPGSANKPKDELDYANDIEKKICKMEKCSSVRTGGSGGDGGSGGDEEAELAYLLGELAYKLGEYRIAIRAYRIALKRDPNNAEAWYNLGNAYYKQGRYREAIEYYQKALELDPNNAEAWYNLGNAYYERGEYEEAIEYYRKALRLDPNNADAMQNLLNAKMREEHHHHHH
CSP X/Z Mosaic	I53-50	CSP-X + CSP-Z 50% valency on I53-50A	Yes	N/A	Yes	Figure 4	Refer to CSP X, CSP Z
CSP X/RT Mosaic	I53-50	CSP-X + RT 50% valency on I53-50A	Yes	N/A	Yes	Figure 4	Refer to CSP X, RT-I53-50A
CSP Z/RT Mosaic	I53-50	CSP-Z + RT 50% valency on I53-50A	Yes	N/A	Yes	Figure 4	Refer to CSP Z, RT-I53-50A
CSP X/Z/RT Mosaic	I53-50	CSP-X + CSP-Z + RT 33% valency on I53-50A	Yes	N/A	Yes	Figure 4	Refer to CSP X, CSP Z, RT-I53-50A
CSP π	I53-dn5	Natural CSP repeats without C term on I53_dn5B	Yes	Degrades	No	N/A	MSSNSKMDPNPDPNANPNVDPNANPNVDPNANPNVDPNANPNPDPNANPNVDPNANPNVDPNANPNVDPNANPNPDPNANPCSSVRTGGSGGDGGSGGDEEAELAYLLGELAYKLGEYRIAIRAYRIALKRDPNNAEAWYNLGNAYYKQGRYREAIEYYQKALELDPNNAEAWYNLGNAYYERGEYEEAIEYYRKALRLDPNNADAMQNLLNAKMREEHHHHHH

CSP ρ	I53-dn5	CSP F on I53_dn5B	No	Expression	No	N/A	MSLGENDDGNNEDNEKLRKPKHKKLKQPADGNPDPNANPNVDPNANPNVDPNANPNVDPNANPNANPNANPNANPNANPNANPNANPNANPNANPNANPNANPNANPNANPNANPNANPNANPNVDPNANPNANPNANPEKAAKAEEAARGGSGGDGGSGGDEEAELAYLLGELAYKLGEYRIAIRAYRIALKRDPNNAEAWYNLGNAYYKQGRYREAIEYYQKALELDPNNAEAWYNLGNAYYERGEYEEAIEYYRKALRLDPNNADAMQNLLNAKMREEHHHHHH
CSP ξ	I53-50	Natural CSP repeats without C term on I53-50A	Yes	Degrades	No	N/A	MSSNSKMDPNPDPNANPNVDPNANPNVDPNANPNVDPNANPNPDPNANPNVDPNANPNVDPNANPNVDPNANPNPDPNANPCSSVRTGSGSKMEELFKKHKIVAVLRANSVEEAIEKAVAVFAGGVHLIEITFTVPDADTVIKALSVLKEKGAIIGAGTVTSVEQCRKAVESGAEFIVSPHLDEEISQFCKEKGVFYMPGVMTPTELVKAMKLGHTILKLFPGEVVGPQFVKAMKGPFPNVKFVPTGGVNLDNVCEWFKAGVLAVGVGSALVKGTPDEVREKAKAFVEKIRGCTEGSHHHHHH
CSP Σ	I53-dn5	C term only on I53_dn5B	Yes	Degrades	No	N/A	MNKNNQGNGQGHNMPNDPNRNVDENANANSAVKNNNNEEPSDKHIKEYLNKIQNSLSTEWSPCSVTCGNGIQVRIKPGSANKPKDELDYANDIEKKICKMEKCSSVRTGGSGGDGGSGGDEEAELAYLLGELAYKLGEYRIAIRAYRIALKRDPNNAEAWYNLGNAYYKQGRYREAIEYYQKALELDPNNAEAWYNLGNAYYERGEYEEAIEYYRKALRLDPNNADAMQNLLNAKMREEHHHHHH
RT-I53_dn5B	I53-dn5	RT on I53-dn5B	Yes	Degrades	No	N/A	MSSNSKMDPNANPNANPNANPNANPNANPNANPNANPNANPNANPNANPNANPNANPNANPNANPNANPNANPNANPNKNNQGNGQGHNMPNDPNRNVDENANANSAVKNNNNEEPSDKHIKEYLNKIQNSLSTEWSPCSVTCGNGIQVRIKPGSANKPKDELDYANDIEKKICKMEKCSSVRTGGSGGDGGSGGDEEAELAYLLGELAYKLGEYRIAIRAYRIALKRDPNNAEAWYNLGNAYYKQGRYREAIEYYQKALELDPNNAEAWYNLGNAYYERGEYEEAIEYYRKALRLDPNNADAMQNLLNAKMREEHHHHHH
CSP-Psi cp 7	I53-dn5 cp7	CSP-Z on dn5A cp7 N-term	No	Degrades	No	N/A	MSSNSKMDPNPDPNANPNVDPNANPNVDPNANPNVDPNANPNPDPNANPNVDPNANPNVDPNANPNVDPNANPNPDPNANPNKNNQGNGQGHNMPNDPNRNVDENANANSAVKNNNNEEPSDKHIKEYLNKIQNSLSTEWSPCSVTCGNGIQVRIKPGSANKPKDELDYANDIEKKICKMEKCSSVRTGSGSDEQAEERAGTKAGNHGEDWGAAAVEMATKFNGDGGSGKYDGSKLRIGILHARGNAEIILELVLGALKRLQEFGVKRENIIIETVPGSFELPYGSKLFVEKQKRLGKPLDAIIPIGVLIRGSTAHFDYIADSTTHQLMKLNFELGIPVIFGVLTTESGGLEHHHHHH
CSP-Psi cp 6	I53-dn5 cp6	CSP-Z on dn5A cp6 N-term	No	Degrades	No	N/A	MSSNSKMDPNPDPNANPNVDPNANPNVDPNANPNVDPNANPNPDPNANPNVDPNANPNVDPNANPNVDPNANPNPDPNANPNKNNQGNGQGHNMPNDPNRNVDENANANSAVKNNNNEEPSDKHIKEYLNKIQNSLSTEWSPCSVTCGNGIQVRIKPGSANKPKDELDYANDIEKKICKMEKCSSVRTGSGSDEQAEERAGTKAGNHGEDWGAAAVEMATKFNGDGGKYDGSKLRIGILHARGNAEIILELVLGALKRLQEFGVKRENIIIETVPGSFELPYGSKLFVEKQKRLGKPLDAIIPIGVLIRGSTAHFDYIADSTTHQLMKLNFELGIPVIFGVLTTESGGLEHHHHHH
CSP-Phi cp 7	I53-dn5 cp7	CSP-Z on dn5A cp7 N,C-term **(Just C_term?)**	Yes	Degrades	No	N/A	MDEQAEERAGTKAGNHGEDWGAAAVEMATKFNGDGGSGKYDGSKLRIGILHARGNAEIILELVLGALKRLQEFGVKRENIIIETVPGSFELPYGSKLFVEKQKRLGKPLDAIIPIGVLIRGSTAHFDYIADSTTHQLMKLNFELGIPVIFGVLTTESGGLEGGSGGDSSNSKMDPNPDPNANPNVDPNANPNVDPNANPNVDPNANPNPDPNANPNVDPNANPNVDPNANPNVDPNANPNPDPNANPGSGSHHHHHH

CSP-Phi cp 6	I53-dn5 cp6	CSP-Z on dn5A cp6 N,C-term **(Just C-term?)**	Yes	Degrades	No	N/A	MDEQAEERAGTKAGNHGEDWGAAAVEMATKFNGDGGKYDGSKLRIGILHARGNAEIILELVLGALKRLQEFGVKRENIIIETVPGSFELPYGSKLFVEKQKRLGKPLDAIIPIGVLIRGSTAHFDYIADSTTHQLMKLNFELGIPVIFGVLTTESGGLEGGSGGDSSNSKMDPNPDPNANPNVDPNANPNVDPNANPNVDPNANPNPDPNANPNVDPNANPNVDPNANPNVDPNANPNPDPNANPGSGSHHHHHH
RT_cp 6	I53-dn5 cp6	RT on N-term of dn5A cp6	Yes	Doesn't Assemble	No	N/A	MSSNSKMDPNANPNANPNANPNANPNANPNANPNANPNANPNANPNANPNANPNANPNANPNANPNANPNANPNANPNANPNKNNQGNGQGHNMPNDPNRNVDENANANSAVKNNNNEEPSDKHIKEYLNKIQNSLSTEWSPCSVTCGNGIQVRIKPGSANKPKDELDYANDIEKKICKMEKCSSVRTGGSGGDGGSGGDDEQAEERAGTKAGNHGEDWGAAAVEMATKFNGDGGKYDGSKLRIGILHARGNAEIILELVLGALKRLQEFGVKRENIIIETVPGSFELPYGSKLFVEKQKRLGKPLDAIIPIGVLIRGSTAHFDYIADSTTHQLMKLNFELGIPVIFGVLTTESGGLEHHHHHH
RT_ cp7	I53-dn5 cp7	RT on N-term of dn5B cp7	Yes	Doesn't Assemble	No	N/A	MSSNSKMDPNANPNANPNANPNANPNANPNANPNANPNANPNANPNANPNANPNANPNANPNANPNANPNANPNANPNANPNKNNQGNGQGHNMPNDPNRNVDENANANSAVKNNNNEEPSDKHIKEYLNKIQNSLSTEWSPCSVTCGNGIQVRIKPGSANKPKDELDYANDIEKKICKMEKCSSVRTGGSGGDGGSGGDDEQAEERAGTKAGNHGEDWGAAAVEMATKFNGDGGSGKYDGSKLRIGILHARGNAEIILELVLGALKRLQEFGVKRENIIIETVPGSFELPYGSKLFVEKQKRLGKPLDAIIPIGVLIRGSTAHFDYIADSTTHQLMKLNFELGIPVIFGVLTTESGGLEHHHHHH
cp 6_RT	I53-dn5 cp6	RT on C-term of dn5B cp6	No		No	N/A	MDEQAEERAGTKAGNHGEDWGAAAVEMATKFNGDGGKYDGSKLRIGILHARGNAEIILELVLGALKRLQEFGVKRENIIIETVPGSFELPYGSKLFVEKQKRLGKPLDAIIPIGVLIRGSTAHFDYIADSTTHQLMKLNFELGIPVIFGVLTTESGGLEGGSGGDGGSGGDSSNSKMDPNANPNANPNANPNANPNANPNANPNANPNANPNANPNANPNANPNANPNANPNANPNANPNANPNANPNANPNKNNQGNGQGHNMPNDPNRNVDENANANSAVKNNNNEEPSDKHIKEYLNKIQNSLSTEWSPCSVTCGNGIQVRIKPGSANKPKDELDYANDIEKKICKMEKCSSVRTHHHHHH
cp 7_RT	I53-dn5 cp7	RT on C-term of dn5B cp7	No		No	N/A	MDEQAEERAGTKAGNHGEDWGAAAVEMATKFNGDGGSGKYDGSKLRIGILHARGNAEIILELVLGALKRLQEFGVKRENIIIETVPGSFELPYGSKLFVEKQKRLGKPLDAIIPIGVLIRGSTAHFDYIADSTTHQLMKLNFELGIPVIFGVLTTESGGLEGGSGGDGGSGGDSSNSKMDPNANPNANPNANPNANPNANPNANPNANPNANPNANPNANPNANPNANPNANPNANPNANPNANPNANPNKNNQGNGQGHNMPNDPNRNVDENANANSAVKNNNNEEPSDKHIKEYLNKIQNSLSTEWSPCSVTCGNGIQVRIKPGSANKPKDELDYANDIEKKICKMEKCSSVRTHHHHHH
RT_cp6_RT	I53-dn5 cp6	RT on N, C-term of dn5B cp6	Yes		No	N/A	MSSNSKMDPNANPNANPNANPNANPNANPNANPNANPNANPNANPNANPNANPNANPNANPNANPNANPNANPNANPNANPNKNNQGNGQGHNMPNDPNRNVDENANANSAVKNNNNEEPSDKHIKEYLNKIQNSLSTEWSPCSVTCGNGIQVRIKPGSANKPKDELDYANDIEKKICKMEKCSSVRTGGSGGDGGSGGDDEQAEERAGTKAGNHGEDWGAAAVEMATKFNGDGGKYDGSKLRIGILHARGNAEIILELVLGALKRLQEFGVKRENIIIETVPGSFELPYGSKLFVEKQKRLGKPLDAIIPIGVLIRGSTAHFDYIADSTTHQLMKLNFELGIPVIFGVLTTESGGLEGGSGGDGGSGGDSSNSKMDPNANPNANPNANPNANPNANPNANPNANPNANPNANPNANPNANPNANPNANPNANPNANPNANPNANPNKNNQGNGQGHNMPNDPNRNVDENANANSAVKNNNNEEPSDKHIKEYLNKIQNSLSTEW

							SPCSVTCGNGIQVRIKPGSANKPKDELDYANDIEKKICKMEKCSSVRTHHHHHH
RT_cp7_RT	I53-dn5 cp7	RT on N, C-term of dn5B cp7	Yes		No	N/A	MSSNSKMDPNANPNANPNANPNANPNANPNANPNANPNANPNANPNANPNANPNANPNANPNANPNANPNANPNANPNKNNQGNGQGHNMPNDPNRNVDENANANSAVKNNNNEEPSDKHIKEYLNKIQNSLSTEWSPCSVTCGNGIQVRIKPGSANKPKDELDYANDIEKKICKMEKCSSVRTGGSGGDGGSGGDDEQAEERAGTKAGNHGEDWGAAAVEMATKFNGDGGSGKYDGSKLRIGILHARGNAEIILELVLGALKRLQEFGVKRENIIIETVPGSFELPYGSKLFVEKQKRLGKPLDAIIPIGVLIRGSTAHFDYIADSTTHQLMKLNFELGIPVIFGVLTTESGGLEGGSGGDGGSGGDSSNSKMDPNANPNANPNANPNANPNANPNANPNANPNANPNANPNANPNANPNANPNANPNANPNANPNKNNQGNGQGHNMPNDPNRNVDENANANSAVKNNNNEEPSDKHIKEYLNKIQNSLSTEWSPCSVTCGNGIQVRIKPGSANKPKDELDYANDIEKKICKMEKCSSVRTHHHHHH
CSP-Omega cp 7	I53-dn5 cp7	CSP-Z on dn5A C-term	Yes		No	N/A	MSSNSKMDPNPDPNANPNVDPNANPNVDPNANPNVDPNANPNPDPNANPNVDPNANPNVDPNANPNVDPNANPNPDPNANPNKNNQGNGQGHNMPNDPNRNVDENANANSAVKNNNNEEPSDKHIKEYLNKIQNSLSTEWSPCSVTCGNGIQVRIKPGSANKPKDELDYANDIEKKICKMEKCSSVRTGSGSDEQAEERAGTKAGNHGEDWGAAAVEMATKFNGDGGSGKYDGSKLRIGILHARGNAEIILELVLGALKRLQEFGVKRENIIIETVPGSFELPYGSKLFVEKQKRLGKPLDAIIPIGVLIRGSTAHFDYIADSTTHQLMKLNFELGIPVIFGVLTTESGGLEGGSGGDSSNSKMDPNPDPNANPNVDPNANPNVDPNANPNVDPNANPNPDPNANPNVDPNANPNVDPNANPNVDPNANPNPDPNANPNKNNQGNGQGHNMPNDPNRNVDENANANSAVKNNNNEEPSDKHIKEYLNKIQNSLSTEWSPCSVTCGNGIQVRIKPGSANKPKDELDYANDIEKKICKMEKCSSVRTHHHHHH
CSP-Omega cp 6	I53-dn5 cp6	CSP-Z on dn5A C-term	Yes		No	N/A	MSSNSKMDPNPDPNANPNVDPNANPNVDPNANPNVDPNANPNPDPNANPNVDPNANPNVDPNANPNVDPNANPNPDPNANPNKNNQGNGQGHNMPNDPNRNVDENANANSAVKNNNNEEPSDKHIKEYLNKIQNSLSTEWSPCSVTCGNGIQVRIKPGSANKPKDELDYANDIEKKICKMEKCSSVRTGSGSDEQAEERAGTKAGNHGEDWGAAAVEMATKFNGDGGKYDGSKLRIGILHARGNAEIILELVLGALKRLQEFGVKRENIIIETVPGSFELPYGSKLFVEKQKRLGKPLDAIIPIGVLIRGSTAHFDYIADSTTHQLMKLNFELGIPVIFGVLTTESGGLEGGSGGDSSNSKMDPNPDPNANPNVDPNANPNVDPNANPNVDPNANPNPDPNANPNVDPNANPNVDPNANPNVDPNANPNPDPNANPNKNNQGNGQGHNMPNDPNRNVDENANANSAVKNNNNEEPSDKHIKEYLNKIQNSLSTEWSPCSVTCGNGIQVRIKPGSANKPKDELDYANDIEKKICKMEKCSSVRTHHHHHH
CSP-Z-PADRE	I53-50	CSP-Z on I53-50A with PADRE peptide on C-term	Yes	Aggregates	No	N/A	MSSNSKMDPNPDPNANPNVDPNANPNVDPNANPNVDPNANPNPDPNANPNVDPNANPNVDPNANPNVDPNANPNPDPNANPNKNNQGNGQGHNMPNDPNRNVDENANANSAVKNNNNEEPSDKHIKEYLNKIQNSLSTEWSPCSVTCGNGIQVRIKPGSANKPKDELDYANDIEKKICKMEKCSSVRTKMEELFKKHKIVAVLRANSVEEAIEKAVAVFAGGVHLIEITFTVPDADTVIKALSVLKEKGAIIGAGTVTSVEQCRKAVESGAEFIVSPHLDEEISQFCKEKGVFYMPGVMTPTELVKAMKLGHDILKLFPGEVVGPQFVKAMKGPFPNVKFVPTGGVNLDNVCEWFKAGVLAVGVGDALVKGDPDEVREKAKKFVEKIRGCTEGSAKFVAAWTLKAAAGSGSHHHHHH

CSP-Xi-PADRE	I53-50	CSP-Z on I53-dn5B with PADRE peptide on C-term	Yes	Aggregates	No	N/A	MSSNSKMDPNPDPNANPNVDPNANPNVDPNANPNVDPNANPNPDPNANPNVDPNANPNVDPNANPNVDPNANPNPDPNANPCSSVRTGSGSKMEELFKKHKIVAVLRANSVEEAIEKAVAVFAGGVHLIEITFTVPDADTVIKALSVLKEKGAIIGAGTVTSVEQCRKAVESGAEFIVSPHLDEEISQFCKEKGVFYMPGVMTPTELVKAMKLGHTILKLFPGEVVGPQFVKAMKGPFPNVKFVPTGGVNLDNVCEWFKAGVLAVGVGSALVKGTPDEVREKAKAFVEKIRGCTEGSAKFVAAWTLKAAAGSGSHHHHHH
CSP-2Z-PADRE	I53-50	CSP-ZX2 on I53-50A with PADRE peptide on C-term	Yes	Aggregates	No	N/A	MSSNSKMDPNPDPNANPNVDPNANPNVDPNANPNVDPNANPNPDPNANPNVDPNANPNVDPNANPNVDPNANPNPDPNANPDPNPDPNANPNVDPNANPNVDPNANPNVDPNANPNPDPNANPNVDPNANPNVDPNANPNVDPNANPNPDPNANPCSSVRTGSGSKMEELFKKHKIVAVLRANSVEEAIEKAVAVFAGGVHLIEITFTVPDADTVIKALSVLKEKGAIIGAGTVTSVEQCRKAVESGAEFIVSPHLDEEISQFCKEKGVFYMPGVMTPTELVKAMKLGHTILKLFPGEVVGPQFVKAMKGPFPNVKFVPTGGVNLDNVCEWFKAGVLAVGVGSALVKGTPDEVREKAKAFVEKIRGCTEGSAKFVAAWTLKAAAGSGSHHHHHH
CSP-2theta-PADRE	I53-dn5	CSP-ZX2 on I53-dn5B with PADRE peptide on C-term	Yes	Aggregates	No	N/A	MSSNSKMDPNPDPNANPNVDPNANPNVDPNANPNVDPNANPNPDPNANPNVDPNANPNVDPNANPNVDPNANPNPDPNANPDPNPDPNANPNVDPNANPNVDPNANPNVDPNANPNPDPNANPNVDPNANPNVDPNANPNVDPNANPNPDPNANPCSSVRTGGSGGDGGSGGDEEAELAYLLGELAYKLGEYRIAIRAYRIALKRDPNNAEAWYNLGNAYYKQGRYREAIEYYQKALELDPNNAEAWYNLGNAYYERGEYEEAIEYYRKALRLDPNNADAMQNLLNAKMREELEHHHHHH
RT_I53-50A_PADRE	I53-50	PADRE on the C terminus of RT-I53-50A	Yes	Degrades	No	N/A	MSSNSKMDPNANPNANPNANPNANPNANPNANPNANPNANPNANPNANPNANPNANPNANPNANPNANPNANPNANPNKNNQGNGQGHNMPNDPNRNVDENANANSAVKNNNNEEPSDKHIKEYLNKIQNSLSTEWSPCSVTCGNGIQVRIKPGSANKPKDELDYANDIEKKICKMEKCSSVRTKMEELFKKHKIVAVLRANSVEEAIEKAVAVFAGGVHLIEITFTVPDADTVIKALSVLKEKGAIIGAGTVTSVEQCRKAVESGAEFIVSPHLDEEISQFCKEKGVFYMPGVMTPTELVKAMKLGHTILKLFPGEVVGPQFVKAMKGPFPNVKFVPTGGVNLDNVCEWFKAGVLAVGVGSALVKGTPDEVREKAKAFVEKIRGCTELEGSAKFVAAWTLKAAAGSHHHHHH
dn5bL1R3_01	I53-dn5	3 sets of NANP repeats in loop 1 and on N-term of I53-dn5B	Yes		No	N/A	MNANPNPDPNANPNVDPNANPGGSGGDGGSGGDEEAELAYLLGELAYKLGEYRIAIRAYRIALKRGNPNANPNANPNAEAWYNLGNAYYKQGRYREAIEYYQKALELDPNNAEAWYNLGNAYYERGEYEEAIEYYRKALRLDPNNADAMQNLLNAKMREELEHHHHHH
dn5bL1R4_02	I53-dn5	4 sets of NANP repeats in loop 1 and on N-term of I53-dn5B	Yes		No	N/A	MNANPNPDPNANPNVDPNANPGGSGGDGGSGGDEEAELAYLLGELAYKLGEYRIAIRAYRIALKRGNPNANPNANPNANPNAEAWYNLGNAYYKQGRYREAIEYYQKALELDPNNAEAWYNLGNAYYERGEYEEAIEYYRKALRLDPNNADAMQNLLNAKMREELEHHHHHH
dn5bL1R5_03	I53-dn5	5 sets of NANP repeats in loop 1 and on N-term of I53-dn5B	Yes		No	N/A	MNANPNPDPNANPNVDPNANPGGSGGDGGSGGDEEAELAYLLGELAYKLGEYRIAIRAYRIALKRGNPNANPNANPNANPNANPNAEAWYNLGNAYYKQGRYREAIEYYQKALELDPNNAEAWYNLGNAYYERGEYEEAIEYYRKALRLDPNNADAMQNLLNAKMREELEHHHHHH
dn5bL2R3_04	I53-dn5	3 sets of NANP repeats in loop 2 and on N-term of I53-dn5B	Yes		No	N/A	MNANPNPDPNANPNVDPNANPGGSGGDGGSGGDEEAELAYLLGELAYKLGEYRIAIRAYRIALKRGNPNANPNANPNANPNAEAWYNLGNAYYKQGRYREAIEYYQKALELDPNNAEAWYNLGNAYYERGEYEEAIEYYRKALRLDPNNADAMQNLLNAKMREELEHHHHHH

dn5bL2R4_05	I53-dn5	4 sets of NANP repeats in loop 2 and on N-term of I53-dn5B	Yes		No	N/A	MNANPNPDPNANPNVDPNANPGGSGGDGGSGGDEEAELAYLLGELAYKLGEYRIAIRAYRIALKRDPNNAEAWYNLGNAYYKQGRYREAIEYYQKALELGNPNANPNANPNANPNAEAWYNLGNAYYERGEYEEAIEYYRKALRLDPNNADAMQNLLNAKMREELEHHHHHH
dn5bL2R5_06	I53-dn5	5 sets of NANP repeats in loop 2 and on N-term of I53-dn5B	Yes		No	N/A	MNANPNPDPNANPNVDPNANPGGSGGDGGSGGDEEAELAYLLGELAYKLGEYRIAIRAYRIALKRDPNNAEAWYNLGNAYYKQGRYREAIEYYQKALELGNPNANPNANPNANPNAEAWYNLGNAYYERGEYEEAIEYYRKALRLDPNNADAMQNLLNAKMREELEHHHHHH
dn5bL12R3_07	I53-dn5	3 sets of NANP repeats in loops 1,2 and on N-term of I53-dn5B	Yes		No	N/A	MNANPNPDPNANPNVDPNANPGGSGGDGGSGGDEEAELAYLLGELAYKLGEYRIAIRAYRIALKRGNPNANPNANPNAEAWYNLGNAYYKQGRYREAIEYYQKALELGNPNANPNANPNAEAWYNLGNAYYERGEYEEAIEYYRKALRLDPNNADAMQNLLNAKMREELEHHHHHH
dn5bL12R4_08	I53-dn5	4 sets of NANP repeats in loops 1,2 and on N-term of I53-dn5B	Yes		No	N/A	MNANPNPDPNANPNVDPNANPGGSGGDGGSGGDEEAELAYLLGELAYKLGEYRIAIRAYRIALKRGNPNANPNANPNANPNAEAWYNLGNAYYKQGRYREAIEYYQKALELGNPNANPNANPNAEAWYNLGNAYYERGEYEEAIEYYRKALRLDPNNADAMQNLLNAKMREELEHHHHHH
dn5bL12R5_09	I53-dn5	5 sets of NANP repeats in loops 1,2 and on N-term of I53-dn5B	Yes		No	N/A	MNANPNPDPNANPNVDPNANPGGSGGDGGSGGDEEAELAYLLGELAYKLGEYRIAIRAYRIALKRGNPNANPNANPNANPNANPNAEAWYNLGNAYYKQGRYREAIEYYQKALELGNPNANPNANPNAEAWYNLGNAYYERGEYEEAIEYYRKALRLDPNNADAMQNLLNAKMREELEHHHHHH
dn5bL1_junctional_10	I53-dn5	Junctional region in loop 1 and on N-term of I53-dn5B	Yes		No	N/A	MNANPNPDPNANPNVDPNANPGGSGGDGGSGGDEEAELAYLLGELAYKLGEYRIAIRAYRIALKRGNANPNPDPNANPNVDPNANPNAEAWYNLGNAYYKQGRYREAIEYYQKALELDPNNAEAWYNLGNAYYERGEYEEAIEYYRKALRLDPNNADAMQNLLNAKMREELEHHHHHH
dn5bL2_junctional_11	I53-dn5	Junctional region in loop 2 and on N-term of I53-dn5B	Yes		No	N/A	MNANPNPDPNANPNVDPNANPGGSGGDGGSGGDEEAELAYLLGELAYKLGEYRIAIRAYRIALKRDPNNAEAWYNLGNAYYKQGRYREAIEYYQKALELGNANPNPDPNANPNVDPNANPNAEAWYNLGNAYYERGEYEEAIEYYRKALRLDPNNADAMQNLLNAKMREELEHHHHHH
dn5bL12_junctional_12	I53-dn5	Junctional region in loops 1,2 and on N-term of I53-dn5B	Yes		No	N/A	MNANPNPDPNANPNVDPNANPGGSGGDGGSGGDEEAELAYLLGELAYKLGEYRIAIRAYRIALKRGNANPNPDPNANPNVDPNANPNAEAWYNLGNAYYKQGRYREAIEYYQKALELGNANPNPDPNANPNVDPNANPNAEAWYNLGNAYYERGEYEEAIEYYRKALRLDPNNADAMQNLLNAKMREELEHHHHHH
dn5bL_013	I53-dn5	peptide 21 in loop 1,2 without an N terminal fusion I53-dn5B	Yes		No	N/A	MEEAELAYLLGELAYKLGEYRIAIRAYRIALKRGNANPNPDPNANPNVDPNANPNAEAWYNLGNAYYKQGRYREAIEYYQKALELGNANPNPDPNANPNVDPNANPNAEAWYNLGNAYYERGEYEEAIEYYRKALRLDPNNADAMQNLLNAKMREELEHHHHHH
dn5bL_014	I53-dn5	peptide 21 in loop 2 without an N terminal fusion I53-dn5B	Yes		No	N/A	MEEAELAYLLGELAYKLGEYRIAIRAYRIALKRDPNNAEAWYNLGNAYYKQGRYREAIEYYQKALELGNANPNPDPNANPNVDPNANPNAEAWYNLGNAYYERGEYEEAIEYYRKALRLDPNNADAMQNLLNAKMREELEHHHHHH
dn5bL_015	I53-dn5	peptide 21 in loop 1 without an N terminal fusion I53-dn5B	Yes		No	N/A	MEEAELAYLLGELAYKLGEYRIAIRAYRIALKRGNANPNPDPNANPNVDPNANPNAEAWYNLGNAYYKQGRYREAIEYYQKALELDPNNAEAWYNLGNAYYERGEYEEAIEYYRKALRLDPNNADAMQNLLNAKMREELEHHHHHH
dn5bL_016	I53-dn5	NPDP in each loop on I53-dn5B	Yes		No	N/A	MEEAELAYLLGELAYKLGEYRIAIRAYRIALKRGNPDPNAEAWYNLGNAYYKQGRYREAIEYYQKALELGNPDPNAEAWYNLGNAYYERGEYEEAIEYYRKALRLDPNNADAMQNLLNAKMREELEHHH

							HHH
dn5bL_017	I53-dn5	NPNV in each loop on I53-dn5B	Yes		No	N/A	MEEAELAYLLGELAYKLGEYRIAIRAYRIALKRGNPNVNAEAWYNLGNAYYKQGRYREAIEYYQKALELGNPNVNAEAWYNLGNAYYERGEYEEAIEYYRKALRLDPNNADAMQNLLNAKMREELEHHHHHH
dn5bL_018	I53-dn5	NPNA in each loop on I53-dn5B	Yes		No	N/A	MEEAELAYLLGELAYKLGEYRIAIRAYRIALKRGNPNANAEAWYNLGNAYYKQGRYREAIEYYQKALELGNPNANAEAWYNLGNAYYERGEYEEAIEYYRKALRLDPNNADAMQNLLNAKMREELEHHHHHH
T33_dn10B_Major_loop1234_01	T33-dn10	NPNA in all loops on T33-dn10B	Yes	N/A	No	N/A	MIEEVVAEMIDILAESSKKSIEELARAADNKTTEKAVAEAIEEIARLATAAIQLIEALAKNLASNGGNPNANPNANPNANPNANPNAGGEEFMARAISAIAELAKKAIEAIYRLADNHTTDTFMARAIAAIANLAVTAILAIAALASNGGNPNANPNANPNANPNANPNAGGEEFMARAISAIAELAKKAIEAIYRLADNHTTDKFMAAAIEAIALLATLAILAIALLASNGGNPNANPNANPNANPNANPNAGGEKFMARAIMAIAILAAKAIEAIYRLADNHTSPTYIEKAIEAIEKIARKAIKAIEMLAKNGGNPNANPNANPNANPNANPNAGGEEYKEKAKKIIDIIRKLAKMAIKKLEDNRTLEHHHHHH
T33_dn10B_Major_loop1234_PADRE_02	T33-dn10	NPNA in all loops + PADRE peptide on T33-dn10B	Yes	N/A	No	N/A	MIEEVVAEMIDILAESSKKSIEELARAADNKTTEKAVAEAIEEIARLATAAIQLIEALAKNLASNGGNPNANPNANPNANPNANPNAGGEEFMARAISAIAELAKKAIEAIYRLADNHTTDTFMARAIAAIANLAVTAILAIAALASNGGNPNANPNANPNANPNANPNAGGEEFMARAISAIAELAKKAIEAIYRLADNHTTDKFMAAAIEAIALLATLAILAIALLASNGGNPNANPNANPNANPNANPNAGGEKFMARAIMAIAILAAKAIEAIYRLADNHTSPTYIEKAIEAIEKIARKAIKAIEMLAKNGGNPNANPNANPNANPNANPNAGGEEYKEKAKKIIDIIRKLAKMAIKKLEDNRTLEDESDESAKFVAAWTLKAAADESDESHHHHHH
T33_dn10B_Junctional_loop1234_03	T33-dn10	NPDP in all loops on T33-dn10B	Yes	N/A	No	N/A	MIEEVVAEMIDILAESSKKSIEELARAADNKTTEKAVAEAIEEIARLATAAIQLIEALAKNLASNGGNPNANPNPDPNANPNVDPNAGGEEFMARAISAIAELAKKAIEAIYRLADNHTTDTFMARAIAAIANLAVTAILAIAALASNGGNPNANPNPDPNANPNVDPNAGGEEFMARAISAIAELAKKAIEAIYRLADNHTTDKFMAAAIEAIALLATLAILAIALLASNGGNPNANPNPDPNANPNVDPNAGGEKFMARAIMAIAILAAKAIEAIYRLADNHTSPTYIEKAIEAIEKIARKAIKAIEMLAKNGGNPNANPNPDPNANPNVDPNAGGEEYKEKAKKIIDIIRKLAKMAIKKLEDNRTLEHHHHHH
T33_dn10B_Junctional_loop1234_PADRE_04	T33-dn10	NPDP in all loops + PADRE peptide on T33-dn10B	Yes	N/A	No	N/A	MIEEVVAEMIDILAESSKKSIEELARAADNKTTEKAVAEAIEEIARLATAAIQLIEALAKNLASNGGNPNANPNPDPNANPNVDPNAGGEEFMARAISAIAELAKKAIEAIYRLADNHTTDTFMARAIAAIANLAVTAILAIAALASNGGNPNANPNPDPNANPNVDPNAGGEEFMARAISAIAELAKKAIEAIYRLADNHTTDKFMAAAIEAIALLATLAILAIALLASNGGNPNANPNPDPNANPNVDPNAGGEKFMARAIMAIAILAAKAIEAIYRLADNHTSPTYIEKAIEAIEKIARKAIKAIEMLAKNGGNPNANPNPDPNANPNVDPNAGGEEYKEKAKKIIDIIRKLAKMAIKKLEDNRTLEDESDESAKFVAAWTLKAAADESDESHHHHHH
T33_dn10B_Minor_loop1234_05	T33-dn10	NPNV in all loops on T33-dn10B	Yes	N/A	No	N/A	MIEEVVAEMIDILAESSKKSIEELARAADNKTTEKAVAEAIEEIARLATAAIQLIEALAKNLASNGGNPNANPNVDPNANPNVDPNAGGEEFMARAISAIAELAKKAIEAIYRLADNHTTDTFMARAIAAIANLAVTAILAIAALASNGGNPNANPNVDPNANPNVDPNAGGEEFMARAISAIAELAKKAIEAIYRLADNHTTDKFMAAAIEAIALLATLAILAIALLASNGGNPNANPNVDPNANPNVDPNAGGEKFMARAIMAIAILAAKAIEAIYRLADNHTSPTYIEKAIEAIEKIARKAIKAIEMLAKNGGNPNANPNVDPNANPNVDPNAGGEEYKEKAKKIIDIIRKLAKMAIKKLEDNRTLEHHHHHH

T33_dn10B_Minor_loop1234_PADRE_06	T33-dn10	NPNV in all loops + PADRE peptide on T33-dn10B	Yes	N/A	No	N/A	MIEEVVAEMIDILAESSKKSIEELARAADNKTTEKAVAEAIEEIARLATAAIQLIEALAKNLASNGGNPNANPNVDPNANPNVDPNAGGEEFMARAISAIAELAKKAIEAIYRLADNHTTDTFMARAIAAIANLAVTAILAIAALASNGGNPNANPNVDPNANPNVDPNAGGEEFMARAISAIAELAKKAIEAIYRLADNHTTDKFMAAAIEAIALLATLAILAIALLASNGGNPNANPNVDPNANPNVDPNAGGEKFMARAIMAIAILAAKAIEAIYRLADNHTSPTYIEKAIEAIEKIARKAIKAIEMLAKNGGNPNANPNVDPNANPNVDPNAGGEEYKEKAKKIIDIIRKLAKMAIKKLEDNRTLEDESDESAKFVAAWTLKAAADESDESHHHHHH
T33_dn10B_Junctional_loop234_07	T33-dn10	NPDP in loops 2,3,4 on T33-dn10B	Yes	N/A	No	N/A	MIEEVVAEMIDILAESSKKSIEELARAADNKTTEKAVAEAIEEIARLATAAIQLIEALAKNLASEEFMARAISAIAELAKKAIEAIYRLADNHTTDTFMARAIAAIANLAVTAILAIAALASNGGNPNANPNPDPNANPNVDPNAGGEEFMARAISAIAELAKKAIEAIYRLADNHTTDKFMAAAIEAIALLATLAILAIALLASNGGNPNANPNPDPNANPNVDPNAGGEKFMARAIMAIAILAAKAIEAIYRLADNHTSPTYIEKAIEAIEKIARKAIKAIEMLAKNGGNPNANPNPDPNANPNVDPNAGGEEYKEKAKKIIDIIRKLAKMAIKKLEDNRTLEHHHHHH
T33_dn10B_Junctional_loop234_PADRE_08	T33-dn10	NPDP in loops 2,3,4 + PADRE peptide on T33-dn10B	Yes	N/A	No	N/A	MIEEVVAEMIDILAESSKKSIEELARAADNKTTEKAVAEAIEEIARLATAAIQLIEALAKNLASEEFMARAISAIAELAKKAIEAIYRLADNHTTDTFMARAIAAIANLAVTAILAIAALASNGGNPNANPNPDPNANPNVDPNAGGEEFMARAISAIAELAKKAIEAIYRLADNHTTDKFMAAAIEAIALLATLAILAIALLASNGGNPNANPNPDPNANPNVDPNAGGEKFMARAIMAIAILAAKAIEAIYRLADNHTSPTYIEKAIEAIEKIARKAIKAIEMLAKNGGNPNANPNPDPNANPNVDPNAGGEEYKEKAKKIIDIIRKLAKMAIKKLEDNRTLEDESDESAKFVAAWTLKAAADESDESHHHHHH
T33_dn10B_Minor_loop234_09	T33-dn10	NPNV in loops 2,3,4 on T33-dn10B	Yes	N/A	No	N/A	MIEEVVAEMIDILAESSKKSIEELARAADNKTTEKAVAEAIEEIARLATAAIQLIEALAKNLASEEFMARAISAIAELAKKAIEAIYRLADNHTTDTFMARAIAAIANLAVTAILAIAALASNGGNPNANPNVDPNANPNVDPNAGGEEFMARAISAIAELAKKAIEAIYRLADNHTTDKFMAAAIEAIALLATLAILAIALLASNGGNPNANPNVDPNANPNVDPNAGGEKFMARAIMAIAILAAKAIEAIYRLADNHTSPTYIEKAIEAIEKIARKAIKAIEMLAKNGGNPNANPNVDPNANPNVDPNAGGEEYKEKAKKIIDIIRKLAKMAIKKLEDNRTLEHHHHHH
T33_dn10B_Minor_loop234_PADRE_10	T33-dn10	NPNV in loops 2,3,4 + PADRE peptide on T33-dn10B	Yes	N/A	No	N/A	MIEEVVAEMIDILAESSKKSIEELARAADNKTTEKAVAEAIEEIARLATAAIQLIEALAKNLASNEEFMARAISAIAELAKKAIEAIYRLADNHTTDTFMARAIAAIANLAVTAILAIAALASNGGNPNANPNVDPNANPNVDPNAGGEEFMARAISAIAELAKKAIEAIYRLADNHTTDKFMAAAIEAIALLATLAILAIALLASNGGNPNANPNVDPNANPNVDPNAGGEKFMARAIMAIAILAAKAIEAIYRLADNHTSPTYIEKAIEAIEKIARKAIKAIEMLAKNGGNPNANPNVDPNANPNVDPNAGGEEYKEKAKKIIDIIRKLAKMAIKKLEDNRTLEDESDESAKFVAAWTLKAAADESDESHHHHHH
T33_dn10B_Major_loop234_11	T33-dn10	NPNA in loops 2,3,4 on T33-dn10B	Yes	N/A	No	N/A	MIEEVVAEMIDILAESSKKSIEELARAADNKTTEKAVAEAIEEIARLATAAIQLIEALAKNLASNEEFMARAISAIAELAKKAIEAIYRLADNHTTDTFMARAIAAIANLAVTAILAIAALASNGGNPNANPNANPNANPNANPNAGGEEFMARAISAIAELAKKAIEAIYRLADNHTTDKFMAAAIEAIALLATLAILAIALLASNGGNPNANPNANPNANPNANPNAGGEKFMARAIMAIAILAAKAIEAIYRLADNHTSPTYIEKAIEAIEKIARKAIKAIEMLAKNGGNPNANPNANPNANPNANPNAGGEEYKEKAKKIIDIIRKLAKMAIKKLEDNRTLEHHHHHH

T33_dn10B_Major_loop234_PADRE_12	T33-dn10	NPNA in loops 2,3,4 + PADRE peptide on T33-dn10B	Yes	N/A	No	N/A	MIEEVVAEMIDILAESSKKSIEELARAADNKTTEKAVAEAIEEIARLATAAIQLIEALAKNLASNEEFMARAISAIAELAKKAIEAIYRLADNHTTDTFMARAIAAIANLAVTAILAIAALASNGGNPNANPNANPNANPNANPNAGGEEFMARAISAIAELAKKAIEAIYRLADNHTTDKFMAAAIEAIALLATLAILAIALLASNGGNPNANPNANPNANPNANPNAGGEKFMARAIMAIAILAAKAIEAIYRLADNHTSPTYIEKAIEAIEKIARKAIKAIEMLAKNGGNPNANPNANPNANPNANPNAGGEEYKEKAKKIIDIIRKLAKMAIKKLEDNRTLEDESDESAKFVAAWTLKAAADESDESHHHHHH
T33_dn10B_Major_loop24_13	T33-dn10	NPNA in loops 2,4 on T33-dn10B	Yes	N/A	No	N/A	MIEEVVAEMIDILAESSKKSIEELARAADNKTTEKAVAEAIEEIARLATAAIQLIEALAKNLASNEEFMARAISAIAELAKKAIEAIYRLADNHTTDTFMARAIAAIANLAVTAILAIAALASNGGNPNANPNANPNANPNANPNAGGEEFMARAISAIAELAKKAIEAIYRLADNHTTDKFMAAAIEAIALLATLAILAIALLASNHTTEKFMARAIMAIAILAAKAIEAIYRLADNHTSPTYIEKAIEAIEKIARKAIKAIEMLAKNGGNPNANPNANPNANPNANPNAGGEEYKEKAKKIIDIIRKLAKMAIKKLEDNRTLEHHHHHH
T33_dn10B_Major_loop24_PADRE_14	T33-dn10	NPNA in loops 2,4 + PADRE peptide on T33-dn10B	Yes	N/A	No	N/A	MIEEVVAEMIDILAESSKKSIEELARAADNKTTEKAVAEAIEEIARLATAAIQLIEALAKNLASNEEFMARAISAIAELAKKAIEAIYRLADNHTTDTFMARAIAAIANLAVTAILAIAALASNGGNPNANPNANPNANPNANPNAGGEEFMARAISAIAELAKKAIEAIYRLADNHTTDKFMAAAIEAIALLATLAILAIALLASNHTTEKFMARAIMAIAILAAKAIEAIYRLADNHTSPTYIEKAIEAIEKIARKAIKAIEMLAKNGGNPNANPNANPNANPNANPNAGGEEYKEKAKKIIDIIRKLAKMAIKKLEDNRTLEDESDESAKFVAAWTLKAAADESDESHHHHHH
T33_dn10B_Minor_loop24_15	T33-dn10	NPNV in loops 2,4 on T33-dn10B	Yes	N/A	No	N/A	MIEEVVAEMIDILAESSKKSIEELARAADNKTTEKAVAEAIEEIARLATAAIQLIEALAKNLASEEFMARAISAIAELAKKAIEAIYRLADNHTTDTFMARAIAAIANLAVTAILAIAALASNGGNPNANPNVDPNANPNVDPNAGGEEFMARAISAIAELAKKAIEAIYRLADNHTTDKFMAAAIEAIALLATLAILAIALLASNHTTEKFMARAIMAIAILAAKAIEAIYRLADNHTSPTYIEKAIEAIEKIARKAIKAIEMLAKNGGNPNANPNVDPNANPNVDPNAGGEEYKEKAKKIIDIIRKLAKMAIKKLEDNRTLEHHHHHH
T33_dn10B_Minor_loop24_PADRE_16	T33-dn10	NPNV in loops 2,4 + PADRE peptide on T33-dn10B	Yes	N/A	No	N/A	MIEEVVAEMIDILAESSKKSIEELARAADNKTTEKAVAEAIEEIARLATAAIQLIEALAKNLASNEEFMARAISAIAELAKKAIEAIYRLADNHTTDTFMARAIAAIANLAVTAILAIAALASNGGNPNANPNVDPNANPNVDPNAGGEEFMARAISAIAELAKKAIEAIYRLADNHTTDKFMAAAIEAIALLATLAILAIALLASNHTTEKFMARAIMAIAILAAKAIEAIYRLADNHTSPTYIEKAIEAIEKIARKAIKAIEMLAKNGGNPNANPNVDPNANPNVDPNAGGEEYKEKAKKIIDIIRKLAKMAIKKLEDNRTLEDESDESAKFVAAWTLKAAADESDESHHHHHH
T33_dn10B_Junctional_loop24_17	T33-dn10	NPDP in loops 2,4 on T33-dn10B	Yes	N/A	No	N/A	MIEEVVAEMIDILAESSKKSIEELARAADNKTTEKAVAEAIEEIARLATAAIQLIEALAKNLASEEFMARAISAIAELAKKAIEAIYRLADNHTTDTFMARAIAAIANLAVTAILAIAALASNGGNPNANPNPDPNANPNVDPNAGGEEFMARAISAIAELAKKAIEAIYRLADNHTTDKFMAAAIEAIALLATLAILAIALLASNHTTEKFMARAIMAIAILAAKAIEAIYRLADNHTSPTYIEKAIEAIEKIARKAIKAIEMLAKNGGNPNANPNPDPNANPNVDPNAGGEEYKEKAKKIIDIIRKLAKMAIKKLEDNRTLEHHHHHH

T33_dn10B_Junctional_loop24_PADRE_18	T33-dn10	NPDP in loops 2,4 + PADRE peptide on T33-dn10B	Yes	N/A	No	N/A	MIEEVVAEMIDILAESSKKSIEELARAADNKTTEKAVAEAIEEIARLATAAIQLIEALAKNLASEEFMARAISAIAELAKKAIEAIYRLADNHTTDTFMARAIAAIANLAVTAILAIAALASNGGNPNANPNPDPNANPNVDPNAGGEEFMARAISAIAELAKKAIEAIYRLADNHTTDKFMAAAIEAIALLATLAILAIALLASNHTTEKFMARAIMAIAILAAKAIEAIYRLADNHTSPTYIEKAIEAIEKIARKAIKAIEMLAKNGGNPNANPNPDPNANPNVDPNAGGEEYKEKAKKIIDIIRKLAKMAIKKLEDNRTLEDESDESAKFVAAWTLKAAADESDESHHHHHH
I52.6_Junctional_Loop1234_01	I52.6	NPDP in all loops on i52.6	Yes	N/A	No	N/A	MSDEEERNELIKRIREAAQRAREAAERTGDPRVRELARELARLAQRAFYLVLHDPSSSDVNEALKLIVEAIEAAVRALEAAERTGDPKVREEARELVRRAVEAAEEVQRNPSSSEVNEKLKAIVVEIEVKVASLEAKEVGGNPNANPNPDPNANPNVDPNAGGPDKALKIAKKVIELALEAVKENPSTEALRAVLEAVRLASEVAKRVGGNPNANPNPDPNANPNVDPNAGGPDKALKIAKLVIELALEAVKEDPSTDALRAVLEAVRLASEVAKRVGGNPNANPNPDPNANPNVDPNAGGPDKALKIAKLVLELAAEAVKEDPSTDALRAAKEAERLATEVAKRVGGNPNANPNPDPNANPNVDPNAGGPKKAREIEMLVLKLQMEAILAETEEVKKEIEESKKRPQSESAKNLILIMQLLINQIRLLALQIRMLALQLQEGSLEWGGHHHHHH
I52.6_Major_Loop1234_02	I52.6	NPNA in all loops on i52.6	Yes	N/A	No	N/A	MSDEEERNELIKRIREAAQRAREAAERTGDPRVRELARELARLAQRAFYLVLHDPSSSDVNEALKLIVEAIEAAVRALEAAERTGDPKVREEARELVRRAVEAAEEVQRNPSSSEVNEKLKAIVVEIEVKVASLEAKEVGGNPNANPNANPNANPNANPNAGGPDKALKIAKKVIELALEAVKENPSTEALRAVLEAVRLASEVAKRVGGNPNANPNANPNANPNANPNAGGPDKALKIAKLVIELALEAVKEDPSTDALRAVLEAVRLASEVAKRVGGNPNANPNANPNANPNANPNAGGPDKALKIAKLVLELAAEAVKEDPSTDALRAAK
I52.6_Minor_Loop1234_03	I52.6	NPNV in all loops on i52.6	Yes	N/A	No	N/A	MSDEEERNELIKRIREAAQRAREAAERTGDPRVRELARELARLAQRAFYLVLHDPSSSDVNEALKLIVEAIEAAVRALEAAERTGDPKVREEARELVRRAVEAAEEVQRNPSSSEVNEKLKAIVVEIEVKVASLEAKEVGGNPNANPNVDPNANPNVDPNAGGPDKALKIAKKVIELALEAVKENPSTEALRAVLEAVRLASEVAKRVGGNPNANPNVDPNANPNVDPNAGGPDKALKIAKLVIELALEAVKEDPSTDALRAVLEAVRLASEVAKRVGGNPNANPNVDPNANPNVDPNAGGPDKALKIAKLVLELAAEAVKEDPSTDALRAAKEAERLATEVAKRVGGNPNANPNVDPNANPNVDPNAGGPKKAREIEMLVLKLQMEAILAETEEVKKEIEESKKRPQSESAKNLILIMQLLINQIRLLALQIRMLALQLQEGSLEWGGHHHHHH
SAmut_CSP_5/3	N/A	PfCSP (3D7) with mutations C25S, K66S, K67S, R70A. Repeat region truncated to include only 5 major repeats and 3 minor repeats.	Yes	N/A	N/A	Not used	QEYQSYGSSSNTRVLNELNYDNAGTNLYNELEMNYYGKQENWYSLSSNSASLGENDDGNNEDNEKLRKPKHKKLKQPADGNPDPNANPNVDPNANPNVDPNANPNVDPNANPNANPNKNNQGNGQGHNMPNDPNRNVDENANANSAVKNNNNEEPSDKHIKEYLNKIQNSLSTEWSPCSVTCGNGIQVRIKPGSANKPKDELDYANDIEKKICKMEKCSGSGLNDIFEAQKIEWHELEVLFQGPGHHHHHH
SAmut_CSP	N/A	PfCSP (3D7) with mutations C25S, K66S, K67S, R70A. 37694.68 kDa	Yes	N/A	N/A	Figure 5	QEYQSYGSSSNTRVLNELNYDNAGTNLYNELEMNYYGKQENWYSLSSNSASLGENDDGNNEDNEKLRKPKHKKLKQPADGNPDPNANPNVDPNANPNVDPNANPNVDPNANPNANPNANPNANPNANPNANPNANPNANPNANPNANPNANPNANPNANPNANPNANPNANPNVDPNANPNANPNANPNANPNANPNANPNANPNANPNANPNANPNANPNANPNANPNANPNANPNANPNANPNANPNKNNQGNGQGHNMPNDPNRNVDENANANSAVKNNNNEEPSDKHIKEYLNKIQNSLSTEWSPCSVTCGNGIQVRIKPGSANKPKDELDYANDIEKKICKMEKCSGSGLNDIFEAQKIEWHELEVLFQGPGHHHHHH

SAmut-CSP-I53-50A	N/A	SAmut construct fused to I53-50A	Yes	N/A	Yes	Figure 5	MQEYQSYGSSSNTRVLNELNYDNAG<br>TNLYNELEMNYYGKQENWYSLSSNS<br>ASLGENDDGNNEDNEKLRKPKHKKL<br>KQPADGNPDPNANPNVDPNANPNVD<br>PNANPNVDPNANPNANPNANPNANP<br>NANPNANPNANPNANPNANPNANPN<br>ANPNANPNANPNANPNANPNANPNA<br>NPNVDPNANPNANPNANPNANPNAN<br>PNANPNANPNANPNANPNANPNANP<br>NANPNANPNANPNANPNANPNANPN<br>ANPNKNNQGNGQGHNMPNDPNRNV<br>DENANANSAVKNNNNEEPSDKHIKEY<br>LNKIQNSLSTEWSPCSVTCGNGIQVR<br>IKPGSANKPKDELDYANDIEKKICKME<br>KCSKMEELFKKHKIVAVLRANSVEEAI<br>EKAVAVFAGGVHLIEITFTVPDADTVIK<br>ALSVLKEKGAIIGAGTVTSVEQCRKAV<br>ESGAEFIVSPHLDEEISQFCKEKGVFY<br>MPGVMTPTELVKAMKLGHDILKLFPG<br>EVVGPQFVKAMKGPFPNVKFVPTGG<br>VNLDNVCEWFKAGVLAVGVGDALVK<br>GDPDEVREKAKKFVEKIRGCTELEHH<br>HHHH
SAmut_CSP_5/3_SpyTag			Yes	N/A	Yes	Figure 1	QEYQSYGSSSNTRVLNELNYDNAGT<br>NLYNELEMNYYGKQENWYSLSSNSA<br>SLGENDDGNNEDNEKLRKPKHKKLK<br>QPADGNPDPNANPNVDPNANPNVDP<br>NANPNVDPNANPNANPNKNNQGNG<br>QGHNMPNDPNRNVDENANANSAVK<br>NNNNEEPSDKHIKEYLNKIQNSLSTE<br>WSPCSVTCGNGIQVRIKPGSANKPK<br>DELDYANDIEKKICKMEKCSGSGLNDI<br>FEAQKIEWHEFRGLISVNHHNVGLEV<br>LFQGPGHHHHHH
SpyCatcher-C4b			Yes	N/A	Yes	Figure 1	MDAMKRGLCCVLLLCGAVFVSPSAS<br>MHHHHHHGAMVDTLSGLSSEQGQS<br>GDMTIEEDSATHIKFSKRDEDGKELA<br>GATMELRDSSGKTISTWISDGQVKDF<br>YLYPGKYTFVETAAPDGYEVATAITFT<br>VNEQGQVTVDGKATKGDAHIGGSGG<br>SSKKQGDADVCGEVAYIQSVVSDCH<br>VPTAELRTLLEIRKLFLEIQKLKVELQG<br>LSKE
SpyCatcher-Ferritin			Yes	N/A	Yes	Figure 1	MDAMKRGLCCVLLLCGAVFVSPSAS<br>MHHHHHHGAMVDTLSGLSSEQGQS<br>GDMTIEEDSATHIKFSKRDEDGKELA<br>GATMELRDSSGKTISTWISDGQVKDF<br>YLYPGKYTFVETAAPDGYEVATAITFT<br>VNEQGQVTVNGKATKGDAHIGGSGG<br>SGESQVRQQFSKDIEKLLNEQVNKE<br>MQSSNLYMSMSSWCYTHSLDGAGLF<br>LFDHAAEEYEHAKKLIIFLNENNVPVQ<br>LTSISAPEHKFEGLTQIFQKAYEHEQHI<br>SESINNIVDHAIKSKDHATFNFLQWYV<br>AEQHEEEVLFKDILDKIELIGNENHGL<br>YLADQYVKGIAKSRKSGSEIPRPRPL<br>ANKGNL
SpyCatcher-I53/50			Yes	N/A	Yes	Figure 1	MHHHHHHSGAMVDTLSGLSSEQGQ<br>SGDMTIEEDSATHIKFSKRDEDGKEL<br>AGATMELRDSSGKTISTWISDGQVKD<br>FYLYPGKYTFVETAAPDGYEVATAITF<br>TVNEQGQVTVNGKATKGDAHIGGSG<br>GSGGKMEELFKKHKIVAVLRANSVEE<br>AIEKAVAVFAGGVHLIEITFTVPDADTV<br>IKALSVLKEKGAIIGAGTVTSVEQCRK<br>AVESGAEFIVSPHLDEEISQFCKEKGV<br>FYMPGVMTPTELVKAMKLGHDILKLF<br>PGEVVGPQFVKAMKGPFPNVKFVPT<br>GGVNLDNVCEWFKAGVLAVGVGDAL<br>VKGDPDEVREKAKKFVEKIRGCTEGS<br>LEHHHHHH
Peptide 20	Peptide	ELISA reagent	N/A	N/A	N/A	N/A	PADGNPDPNANPNVD
Peptide 21	Peptide	ELISA reagent	N/A	N/A	N/A	N/A	NPDPNANPNVDPNAN
Peptide 22	Peptide	ELISA reagent	N/A	N/A	N/A	N/A	NANPNVDPNANPNVD
Peptide 23	Peptide	ELISA reagent	N/A	N/A	N/A	N/A	NVDPNANPNVDPNAN
Peptide 27	Peptide	ELISA reagent	N/A	N/A	N/A	N/A	NVDPNANPNANPNAN
Peptide 29	Peptide	ELISA reagent	N/A	N/A	N/A	N/A	NANPNANPNANPNAN
Peptide 43	Peptide	ELISA reagent	N/A	N/A	N/A	N/A	NANPNANPNVDPNAN
Peptide 44	Peptide	ELISA reagent	N/A	N/A	N/A	N/A	NANPNVDPNANPNAN

Peptide 61	Peptide	ELISA reagent	N/A	N/A	N/A	N/A	NANPNANPNANPNKN
Peptide 20-23	Peptide	ELISA reagent	N/A	N/A	N/A	N/A	PADGNPDPNANPNVDPNANPNVDPN AN
Repeat Peptide	Peptide	ELISA reagent	N/A	N/A	N/A	N/A	NANPNANPNANPNANPNANPNANPN ANPNANPNANP

Supplementary Table 1. Table of all protein constructs and their sequences used in this study. Description of the scaffold, expression status, point of failure, nanoparticle production status, corresponding mouse study figure, and sequence are provided. The peptides listed were used for ELISA experiments.
