# Supplementary material for: Elicitation of liver-stage immunity by nanoparticle immunogens displaying P. falciparum CSP-derived antigens
Source: NPJ Vaccines. 2025 May 5;10:87. doi: 10.1038/s41541-025-01140-x (PMC12053698; doi:10.1038/s41541-025-01140-x)
Supplement: Supplementary file 2 — Supplementary Table 1 [file 41541_2025_1140_MOESM2_ESM.pdf]

Plasmid Name	Parent Scaffold	Description	Expressed?	Point of Failure	Nanoparticle Made?	Mouse Study	Sequence
**RT-I53-50**	I53-50	18 NANP repeats and C-term of CSP on I53-50A	Yes	N/A	Yes	Figure 2, 3, 4, 5	MSSNSKMDPNANPNANPNANPNANPNANPNANPNANPNANPNANPNANPNANPNANPNANPNANPNANPNANPNANPNANPNANPNKNNQGNGQGHNMPNDPNRNVDENANANSAVKNNNNEEPSDKHIKEYLNKIQNSLSTEWSPCSVTCGNGIQVRIKPGSANKPKDELDYANDIEKKICKMEKCSSVRTMKMEELFKKHKIVAVLRANSVEEAIEKAVAVFAGGVHLIEITFTVPDADTVIKALSVLKEKGAIIGAGTVTSVEQCRKAVESGAEFIVSPHLDEEISQFCKEKGVFYMPGVMTPTELVKAMKLGHTILKLFPGEVVGPQFVKAMKGPFPNVKFVPTGGVNLDNVCEWFKAGVLAVGVGSALVKGTPDEVREKAKAFVEKIRGCTELEHHHHHH
**CSP-A**	I53-50	N-term + R1 + Natural Repeats + 7 NANPs on I53-50A	Yes	Aggregates	No	N/A	MQEYQSYGSSSNTRVLNELNYDNAGTNLYNELEMNYYGKQENWYSLKKNSRSLGENDDGNNEDNEKLRKPKHKKLKQPADGNPDPNANPNVDPNANPNVDPNANPNVDPNANPNANPNANPNANPNANPEKAAKAEEAARKMEELFKKHKIVAVLRANSVEEAIEKAVAVFAGGVHLIEITFTVPDADTVIKALSVLKEKGAIIGAGTVTSVEQCRKAVESGAEFIVSPHLDEEISQFCKEKGVFYMPGVMTPTELVKAMKLGHDILKLFPGEVVGPQFVKAMKGPFPNVKFVPTGGVNLDNVCEWFKAGVLAVGVGDALVKGDPDEVREKAKKFVEKIRGCTELEHHHHHH
**CSP-B**	I53-50	N-term + R1 + Natural Repeats - NPDP + 7 NANPs on I53-50A	Yes	Aggregates	No	N/A	MQEYQSYGSSSNTRVLNELNYDNAGTNLYNELEMNYYGKQENWYSLKKNSRSLGENDDGNNEDNEKLRKPKHKKLKQPADGNANPNANPNVDPNANPNVDPNANPNVDPNANPNANPNANPNANPNANPEKAAKAEEAARKMEELFKKHKIVAVLRANSVEEAIEKAVAVFAGGVHLIEITFTVPDADTVIKALSVLKEKGAIIGAGTVTSVEQCRKAVESGAEFIVSPHLDEEISQFCKEKGVFYMPGVMTPTELVKAMKLGHDILKLFPGEVVGPQFVKAMKGPFPNVKFVPTGGVNLDNVCEWFKAGVLAVGVGDALVKGDPDEVREKAKKFVEKIRGCTELEHHHHHH
**CSP-C**	I53-50	N term + R1 + NPDP on I53-50A	Yes	Aggregates	No	N/A	MQEYQSYGSSSNTRVLNELNYDNAGTNLYNELEMNYYGKQENWYSLKKNSRSLGENDDGNNEDNEKLRKPKHKKLKQPADGNPDPNANPNVDPNANEKAAKAEEAARKMEELFKKHKIVAVLRANSVEEAIEKAVAVFAGGVHLIEITFTVPDADTVIKALSVLKEKGAIIGAGTVTSVEQCRKAVESGAEFIVSPHLDEEISQFCKEKGVFYMPGVMTPTELVKAMKLGHDILKLFPGEVVGPQFVKAMKGPFPNVKFVPTGGVNLDNVCEWFKAGVLAVGVGDALVKGDPDEVREKAKKFVEKIRGCTELEHHHHHH

**CSP-D**	I53-50	N-term + R1 + Natural Repeats on I53-50A	Yes	Aggregates	No	N/A	MQEYQSYGSSSNTRVLNELNY<br>DNAGTNLYNELEMNYYGKQEN<br>WYSLKKNSRSLGENDDGNNED<br>NEKLRKPKHKKLKQPADGNPDP<br>NANPNVDPNANPNVDPNANPN<br>VDPNANEKAAKAEEAARKMEEL<br>FKKHKIVAVLRANSVEEAIEKAVA<br>VFAGGVHLIEITFTVPDADTVIKA<br>LSVLKEKGAIIGAGTVTSVEQCR<br>KAVESGAEFIVSPHLDEEISQFC<br>KEKGVFYMPGVMTPTELVKAM<br>KLGHDILKLFPGEVVGPQFVKA<br>MKGPFPNVKFVPTGGVNLDNV<br>CEWFKAGVLAVGVGDALVKGD<br>PDEVREKAKKFVEKIRGCTELE<br>HHHHHH
**CSP-E**	I53-50	N-term + R1 + Natural Repeats - NPDP + 3 NANPs on I53-50A	Yes	Aggregates	No	N/A	MQEYQSYGSSSNTRVLNELNY<br>DNAGTNLYNELEMNYYGKQEN<br>WYSLKKNSRSLGENDDGNNED<br>NEKLRKPKHKKLKQPADGNPDP<br>NANPNVDPNANPNVDPNANPN<br>VDPNANPNANPNANPNANEKA<br>AKAEEAARKMEELFKKHKIVAVL<br>RANSVEEAIEKAVAVFAGGVHLI<br>EITFTVPDADTVIKALSVLKEKG<br>AIIGAGTVTSVEQCRKAVESGAE<br>FIVSPHLDEEISQFCKEKGVFYM<br>PGVMTPTELVKAMKLGHDILKLF<br>PGEVVGPQFVKAMKGPFPNVK<br>FVPTGGVNLDNVCEWFKAGVL<br>AVGVGDALVKGDPDEVREKAKK<br>FVEKIRGCTELEHHHHHH
**CSP-F**	I53-50	R1 + Natural Repeats + NANP, NVDP repeats on I53-50A	Yes	N/A	Yes	Figure 2	MSLGENDDGNNEDNEKLRKPK<br>HKKLKQPADGNPDPNANPNVD<br>PNANPNVDPNANPNVDPNANP<br>NANPNANPNANPNANPNANPN<br>ANPNANPNANPNANPNANPNA<br>NPNANPNANPNANPNANPNAN<br>PNVDPNANPNANPNANPEKAA<br>KAEEAARKMEELFKKHKIVAVLR<br>ANSVEEAIEKAVAVFAGGVHLIEI<br>TFTVPDADTVIKALSVLKEKGAII<br>GAGTVTSVEQCRKAVESGAEFI<br>VSPHLDEEISQFCKEKGVFYMP<br>GVMTPTELVKAMKLGHDILKLFP<br>GEVVGPQFVKAMKGPFPNVKF<br>VPTGGVNLDNVCEWFKAGVLA<br>VGVGDALVKGDPDEVREKAKKF<br>VEKIRGCTELEHHHHHH
**CSP-G**	I53-50	CSP F + N-term on I53-50A	Yes	Aggregates	No	N/A	MQEYQSYGSSSNTRVLNELNY<br>DNAGTNLYNELEMNYYGKQEN<br>WYSLKKNSRSLGENDDGNNED<br>NEKLRKPKHKKLKQPADGNPDP<br>NANPNVDPNANPNVDPNANPN<br>VDPNANPNANPNANPNANPNA<br>NPNANPNANPNANPNANPNAN<br>PNANPNANPNANPNANPNANP<br>NANPNANPNVDPNANPNANPN<br>ANPEKAAKAEEAARKMEELFKK<br>HKIVAVLRANSVEEAIEKAVAVFA<br>GGVHLIEITFTVPDADTVIKALSV<br>LKEKGAIIGAGTVTSVEQCRKAV<br>ESGAEFIVSPHLDEEISQFCKEK<br>GVFYMPGVMTPTELVKAMKLG<br>HDILKLFPGEVVGPQFVKAMKG<br>PFPNVKFVPTGGVNLDNVCEW<br>FKAGVLAVGVGDALVKGDPDEV<br>REKAKKFVEKIRGCTELEHHHH<br>HH

**CSP-H**	I53-50	N-term + R1 + NPDP and NVDP repeat with 15 NANP on I53-50A	Yes	Aggregates	No	N/A	MQEYQSYGSSSNTRVLNELNY DNAGTNLYNELEMNYYGKQEN WYSLKKNSRSLGENDDGNNED NEKLRKPKHKKLKQPADGNPDP NANPNVDPNANPNANPNANPN ANPNANPNANPNANPNANPNA NPNANPNANPNANPNANPNAN PNANPNANPNANPNANPNANP NANPNANPEKAAKAEEAARKM EELFKKHKIVAVLRANSVEEAIEK AVAVFAGGVHLIEITFTVPDADTV IKALSVLKEKGAIIGAGTVTSVEQ CRKAVESGAEFIVSPHLDEEISQ FCKEKGVFYMPGVMTPTELVKA MKLGHDILKLFPGEVVGPQFVK AMKGPFPNVKFVPTGGVNLDN VCEWFKAGVLAVGVGDALVKG DPDEVREKAKKFVEKIRGCTEL EHHHHHH
**CSP-A2**	I53-50	CSP-A without N-term on I53-50A	Yes	Aggregates	No	N/A	MSLGENDDGNNEDNEKLRKPK HKKLKQPADGNPDPNANPNVD PNANPNVDPNANLNVDPNANP NANPNANPNANPNANPNANPE KAAKAEEAARKMEELFKKHKIVA VLRANSVEEAIEKAVAVFAGGVH LIEITFTVPDADTVIKALSVLKEK GAIIGAGTVTSVEQCRKAVESG AEFIVSPHLDEEISQFCKEKGVF YMPGVMTPTELVKAMKLGHDIL KLFPGEVVGPQFVKAMKGPFP NVKFVPTGGVNLDNVCEWFKA GVLAVGVGDALVKGDPDEVREK AKKFVEKIRGCTELEHHHHHH
**CSP-B2**	I53-50	CSP-B without N-term on I53-50A	Yes	Aggregates	No	N/A	MSLGENDDGNNEDNEKLRKPK HKKLKQPADGNANPNANPNVD PNANPNVDPNANPNVDPNANP NANPNANPNANPNANPNANPE KAAKAEEAARKMEELFKKHKIVA VLRANSVEEAIEKAVAVFAGGVH LIEITFTVPDADTVIKALSVLKEK GAIIGAGTVTSVEQCRKAVESG AEFIVSPHLDEEISQFCKEKGVF YMPGVMTPTELVKAMKLGHDIL KLFPGEVVGPQFVKAMKGPFP NVKFVPTGGVNLDNVCEWFKA GVLAVGVGDALVKGDPDEVREK AKKFVEKIRGCTELEHHHHHH
**CSP-C2**	I53-50	CSP-C without N-term on I53-50A	Yes	Aggregates	No	N/A	MSLGENDDGNNEDNEKLRKPK HKKLKQPADGNPDPNANPNVD PNANEKAAKAEEAARKMEELFK KHKIVAVLRANSVEEAIEKAVAVF AGGVHLIEITFTVPDADTVIKALS VLKEKGAIIGAGTVTSVEQCRKA VESGAEFIVSPHLDEEISQFCKE KGVFYMPGVMTPTELVKAMKL GHDILKLFPGEVVGPQFVKAMK GPFPNVKFVPTGGVNLDNVCE WFKAGVLAVGVGDALVKGDPD EVREKAKKFVEKIRGCTELEHH HHHH
**CSP-D2**	I53-50	CSP-D without N-term on It 53-50	Yes	Aggregates	No	N/A	MSLGENDDGNNEDNEKLRKPK HKKLKQPADGNPDPNANPNVD PNANPNVDPNANPNVDPNANE KAAKAEEAARKMEELFKKHKIVA VLRANSVEEAIEKAVAVFAGGVH LIEITFTVPDADTVIKALSVLKEK GAIIGAGTVTSVEQCRKAVESG AEFIVSPHLDEEISQFCKEKGVF YMPGVMTPTELVKAMKLGHDIL KLFPGEVVGPQFVKAMKGPFP NVKFVPTGGVNLDNVCEWFKA GVLAVGVGDALVKGDPDEVREK AKKFVEKIRGCTELEHHHHHH

**CSP-E2**	I53-50	CSP-E without N-term on I53-50A	Yes	Aggregates	No	N/A	MSLGENDDGNNEDNEKLRKPK<br>HKKLKQPADGNPDPNANPNVD<br>PNANPNVDPNANPNVDPNANP<br>NANPNANPNANEKAAKAEEAA<br>RKMEELFKKHKIVAVLRANSVEE<br>AIEKAVAVFAGGVHLIEITFTVPD<br>ADTVIKALSVLKEKGAIIGAGTVT<br>SVEQCRKAVESGAEFIVSPHLD<br>EEISQFCKEKGVFYMPGVMTPT<br>ELVKAMKLGHDILKLFPGEVVGP<br>QFVKAMKGPFPNVKFVPTGGV<br>NLDNVCEWFKAGVLAVGVGDA<br>LVKGDPDEVREKAKKFVEKIRG<br>CTELEHHHHH
**CSP-G2**	I53-50	CSP-G without N-term on I53-50A	Yes	Aggregates	No	N/A	MSLGENDDGNNEDNEKLRKPK<br>HKKLKQPADGNPDPNANPNVD<br>PNANPNVDPNANPNVDPNANP<br>NANPNANPNANPNANPNANPN<br>ANPNANPNANPNANPNANPNA<br>NPNANPNANPNANPNANPNAN<br>PNVDPNANPNANPNANPEKAA<br>KAEEAARKMEELFKKHKIVAVLR<br>ANSVEEAIEKAVAVFAGGVHLIEI<br>TFTVPDADTVIKALSVLKEKGAII<br>GAGTVTSVEQCRKAVESGAEFI<br>VSPHLDEEISQFCKEKGVFYMP<br>GVMTPTELVKAMKLGHDILKLFP<br>GEVVGPQFVKAMKGPFPNVKF<br>VPTGGVNLDNVCEWFKAGVLA<br>VGVGDALVKGDPDEVREKAKKF<br>VEKIRGCTELEHHHHHH
**CSP-H2**	I53-50	CSP-H without N-term on I53-50A	Yes	Aggregates	No	N/A	MSLGENDDGNNEDNEKLRKPK<br>HKKLKQPADGNPDPNANPNVD<br>PNANPNANPNANPNANPNANP<br>NANPNANPNANPNANPNANPN<br>ANPNANPNANPNANPNANPNA<br>NPNANPNANPNANPNANPNAN<br>PEKAAKAEEAARKMEELFKKHK<br>IVAVLRANSVEEAIEKAVAVFAGG<br>VHLIEITFTVPDADTVIKALSVLK<br>EKGAIIGAGTVTSVEQCRKAVES<br>GAEFIVSPHLDEEISQFCKEKGV<br>FYMPGVMTPTELVKAMKLGHDI<br>LKLFPGEVVGPQFVKAMKGPFP<br>NVKFVPTGGVNLDNVCEWFKA<br>GVLAVGVGDALVKGDPDEVREK<br>AKKFVEKIRGCTELEHHHHHH
**CSP-J**	I53-50	R1 + Natural Repeats + 17 NANPs on I53-50A	Yes	N/A	Yes	Figure 2	MSLGENDDGNNEDNEKLRKPK<br>HKKLKQPADGNPDPNANPNVD<br>PNANPNVDPNANPNVDPNANP<br>NANPNANPNANPNANPNANPN<br>ANPNANPNANPNANPNANPNA<br>NPNANPNANPNANPNANPNAN<br>PNANPNANPNKNNQGNGQGH<br>NMPNDPNRNVDENANANSAVK<br>NNNNEEPSDKHIKEYLNKIQNSL<br>STEWSPCSVTCGNGIQVRIKPG<br>SANKPKDELDYANDIEKKICKME<br>KCSSVRTMKMEELFKKHKIVAVL<br>RANSVEEAIEKAVAVFAGGVHLI<br>EITFTVPDADTVIKALSVLKEKG<br>AIIGAGTVTSVEQCRKAVESGAE<br>FIVSPHLDEEISQFCKEKGVFYM<br>PGVMTPTELVKAMKLGHTILKLF<br>PGEVVGPQFVKAMKGPFPNVK<br>FVPTGGVNLDNVCEWFKAGVL<br>AVGVGSALVKGTPDEVREKAKA<br>FVEKIRGCTELEHHHHHH

**CSP-K**	I53-50	Natural Repeats + 17 NANPS on I53-50A	Yes	N/A	Yes	Figure 2	MSSNSKMDPNPDPNANPNVDPNANPNVDPNANPNVDPNANPNANPNANPNANPNANPNANPNANPNANPNANPNANPNANPNANPNANPNANPNANPNANPNANPNANPNKNNQGNGQGHNMPNDPNRNVDENANANSAVKNNNNEEPSDKHIKEYLNKIQNSLSTEWSPCSVTCGNGIQVRIKPGSANKPKDELDYANDIEKKICKMEKCSSVRTMKMEELFKKHKIVAVLRANSVEEAIEKAVAVFAGGVHLIEITFTVPDADTVIKALSVLKEKGAIIGAGTVTSVEQCRKAVESGAEFIVSPHLDEEISQFCKEKGVFYMPGVMTPTELVKAMKLGHTILKLFPGEVVGPQFVKAMKGPFPNVKFVPTGGVNLDNVCEWFKAGVLAVGVGSALVKGTPDEVREKAKAFVEKIRGCTELEHHHHHH
**CSP-L**	I53-50	Full Set of repeats on I53-50A	No	Expression	No	N/A	MSSNSKMDPNPDPNANPNVDPNANPNVDPNANPNVDPNANPNANPNANPNANPNANPNANPNANPNANPNANPNANPNANPNANPNANPNANPNANPNVDPNANPNANPNANPNANPNANPNANPNANPNANPNANPNANPNANPNANPNANPNANPNANPNANPNANPNKNNQGNGQGHNMPNDPNRNVDENANANSAVKNNNNEEPSDKHIKEYLNKIQNSLSTEWSPCSVTCGNGIQVRIKPGSANKPKDELDYANDIEKKICKMEKCSSVRTMKMEELFKKHKIVAVLRANSVEEAIEKAVAVFAGGVHLIEITFTVPDADTVIKALSVLKEKGAIIGAGTVTSVEQCRKAVESGAEFIVSPHLDEEISQFCKEKGVFYMPGVMTPTELVKAMKLGHDILKLFPGEVVGPQFVKAMKGPFPNVKFVPTGGVNLDNVCEWFKAGVLAVGVGDALVKGDPDEVREKAKKFVEKIRGCTELEHHHHHH
**CSP-M**	I53-50	R1 + Full set of repeats on I53-50A	No	Expression	No	N/A	MSLGENDDGNNEDNEKLRKPKHKKLKQPADGNPDPNANPNVDPNANPNVDPNANPNVDPNANPNANPNANPNANPNANPNANPNANPNANPNANPNANPNANPNANPNANPNANPNANPNVDPNANPNANPNANPNANPNANPNANPNANPNANPNANPNANPNANPNANPNANPNANPNANPNANPNKNNQGNGQGHNMPNDPNRNVDENANANSAVKNNNNEEPSDKHIKEYLNKIQNSLSTEWSPCSVTCGNGIQVRIKPGSANKPKDELDYANDIEKKICKMEKCSSVRTMKMEELFKKHKIVAVLRANSVEEAIEKAVAVFAGGVHLIEITFTVPDADTVIKALSVLKEKGAIIGAGTVTSVEQCRKAVESGAEFIVSPHLDEEISQFCKEKGVFYMPGVMTPTELVKAMKLGHDILKLFPGEVVGPQFVKAMKGPFPNVKFVPTGGVNLDNVCEWFKAGVLAVGVGDALVKGDPDEVREKAKKFVEKIRGCTELEHHHHHH

**CSP-P**	I53-50	R1 + natural Repeats + C term on I53-50A	Yes	N/A	Yes	Figure 2	MSLGENDDGNNEDNEKLRKPKHKKLKQPADGNPDPNANPNVDPNANPNVDPNANPNVDPNANPNKNNQGNGQGHNMPNDPNRNVDENANANSAVKNNNNEEPSDKHIKEYLNKIQNSLSTEWSPCSVTCGNGIQVRIKPGSANKPKDELDYANDIEKKICKMEKCSSVRTMKMEELFKKHKIVAVLRANSVEEAIEKAVAVFAGGVHLIEITFTVPDADTVIKALSVLKEKGAIIGAGTVTSVEQCRKAVESGAEFIVSPHLDEEISQFCKEKGVFYMPGVMTPTELVKAMKLGHTILKLFPGEVVGPQFVKAMKGPFPNVKFVPTGGVNLDNVCEWFKAGVLAVGVGSALVKGTPDEVREKAKAFVEKIRGCTELEHHHHHH
**CSP-Q**	I53-50	R1 + natural Repeats + 3 NANP + C term on I53-50A	Yes	N/A	Yes	Figure 2	MSLGENDDGNNEDNEKLRKPKHKKLKQPADGNPDPNANPNVDPNANPNVDPNANPNVDPNANPNANPNANPNANPNKNNQGNGQGHNMPNDPNRNVDENANANSAVKNNNNEEPSDKHIKEYLNKIQNSLSTEWSPCSVTCGNGIQVRIKPGSANKPKDELDYANDIEKKICKMEKCSSVRTMKMEELFKKHKIVAVLRANSVEEAIEKAVAVFAGGVHLIEITFTVPDADTVIKALSVLKEKGAIIGAGTVTSVEQCRKAVESGAEFIVSPHLDEEISQFCKEKGVFYMPGVMTPTELVKAMKLGHTILKLFPGEVVGPQFVKAMKGPFPNVKFVPTGGVNLDNVCEWFKAGVLAVGVGSALVKGTPDEVREKAKAFVEKIRGCTELEHHHHHH
CSP-R	I53-50	R1 + natural Repeats + 6 NANP + C term on I53-50A	Yes	N/A	Yes	Figure 2	MSLGENDDGNNEDNEKLRKPKHKKLKQPADGNPDPNANPNVDPNANPNVDPNANPNVDPNANPNANPNANPNANPNANPNANPNANPNKNNQGNGQGHNMPNDPNRNVDENANANSAVKNNNNEEPSDKHIKEYLNKIQNSLSTEWSPCSVTCGNGIQVRIKPGSANKPKDELDYANDIEKKICKMEKCSSVRTMKMEELFKKHKIVAVLRANSVEEAIEKAVAVFAGGVHLIEITFTVPDADTVIKALSVLKEKGAIIGAGTVTSVEQCRKAVESGAEFIVSPHLDEEISQFCKEKGVFYMPGVMTPTELVKAMKLGHTILKLFPGEVVGPQFVKAMKGPFPNVKFVPTGGVNLDNVCEWFKAGVLAVGVGSALVKGTPDEVREKAKAFVEKIRGCTELEHHHHHH
CSP-J2	I53-dn5	CSP-J C-terminal Fusion to I53-dn5B	Yes	Degrades	No	N/A	
CSP-K2	I53-dn5	CSP-K C-terminal Fusion to I53-dn5B	Yes	Degrades	No	N/A	
CSP-L2	I53-dn5	CSP-L C-terminal Fusion to I53-dn5B	Yes	Degrades	No	N/A	
CSP-M2	I53-dn5	CSP-M C-terminal Fusion to I53-dn5B	Yes	Degrades	No	N/A	
CSP-P2	I53-dn5	CSP-P C-terminal Fusion to I53-dn5B	Yes	Degrades	No	N/A	
CSP-Q2	I53-dn5	CSP-Q C-terminal Fusion to I53-dn5B	Yes	Degrades	No	N/A	
CSP-R2	I53-dn5	CSP-R C-terminal Fusion to I53-dn5B	Yes	Degrades	No	N/A	

CSP-J3	I53-dn5	CSP-J N-terminal Fusion to I53-dn5B	Yes	Degrades	No	N/A	MSSNSKMDPLGENDDGNNEDNEKLRKPKHKKLKQPADGNPDPNANPNVDPNANPNVDPNANPNVDPNANPNANPNANPNANPNANPNANPNANPNANPNANPNANPNANPNANPNANPNANPNANPNANPNANPNANPNKNNQGNGQGHNMPNDPNRNVDENANANSAVKNNNNEEPSDKHIKEYLNKIQNSLSTEWSPCSVTCGNGIQVRIKPGSANKPKDELDYANDIEKKICKMEKCSSVRTMLEGGSGGDGGSGGDEEAELAYLLGELAYKLGEYRIAIRAYRIALKRDPNNAEAWYNLGNAYYKQGRYREAIEYYQKALELDPNNAEAWYNLGNAYYERGEYEEAIEYYRKALRLDPNNADAMQNLLNAKMREEHHHHHH
CSP-K3	I53-dn5	CSP-K N-terminal Fusion to I53-dn5B	Yes	Degrades	No	N/A	MSSNSKMDPNPDPNANPNVDPNANPNVDPNANPNVDPNANPNANPNANPNANPNANPNANPNANPNANPNANPNANPNANPNANPNANPNANPNANPNANPNANPNANPNANPNANPNKNNQGNGQGHNMPNDPNRNVDENANANSAVKNNNNEEPSDKHIKEYLNKIQNSLSTEWSPCSVTCGNGIQVRIKPGSANKPKDELDYANDIEKKICKMEKCSSVRTMLEGGSGGDGGSGGDEEAELAYLLGELAYKLGEYRIAIRAYRIALKRDPNNAEAWYNLGNAYYKQGRYREAIEYYQKALELDPNNAEAWYNLGNAYYERGEYEEAIEYYRKALRLDPNNADAMQNLLNAKMREEHHHHHH
CSP-L3	I53-dn5	CSP-L N-terminal Fusion to I53-dn5B	Yes	Degrades	No	N/A	MSSNSKMDPNPDPNANPNVDPNANPNVDPNANPNVDPNANPNANPNANPNANPNANPNANPNANPNANPNANPNANPNANPNANPNANPNANPNANPNVDPNANPNANPNANPNANPNANPNANPNANPNANPNANPNANPNANPNANPNANPNANPNANPNANPNANPNANPNANPNKNNQGNGQGHNMPNDPNRNVDENANANSAVKNNNNEEPSDKHIKEYLNKIQNSLSTEWSPCSVTCGNGIQVRIKPGSANKPKDELDYANDIEKKICKMEKCSSVRTMLEGGSGGDGGSGGDEEAELAYLLGELAYKLGEYRIAIRAYRIALKRDPNNAEAWYNLGNAYYKQGRYREAIEYYQKALELDPNNAEAWYNLGNAYYERGEYEEAIEYYRKALRLDPNNADAMQNLLNAKMREEHHHHHH
CSP-M3	I53-dn5	CSP-M N-terminal Fusion to I53-dn5B	Yes	Degrades	No	N/A	MSSNSKMDPSLGENDDGNNEDNEKLRKPKHKKLKQPADGNPDPNANPNVDPNANPNVDPNANPNVDPNANPNANPNANPNANPNANPNANPNANPNANPNANPNANPNANPNANPNANPNANPNVDPNANPNANPNANPNANPNANPNANPNANPNANPNANPNANPNANPNANPNANPNANPNANPNANPNANPNKNNQGNGQGHNMPNDPNRNVDENANANSAVKNNNNEEPSDKHIKEYLNKIQNSLSTEWSPCSVTCGNGIQVRIKPGSANKPKDELDYANDIEKKICKMEKCSSVRTMLEGGSGGDGGSGGDEEAELAYLLGELAYKLGEYRIAIRAYRIALKRDPNNAEAWYNLGNAYYKQGRYREAIEYYQKALELDPNNAEAWYNLGNAYYERGEYEEAIEYYRKA

							LRLDPNNADAMQNLLNAKMREEHHHHHH
CSP-P3	I53-dn5	CSP-P N-terminal Fusion to I53-dn5B	Yes	Degrades	No	N/A	MSSNSKMDPSLGENDDGNNEDNEKLRKPKHKKLKQPADGNPDPNANPNVDPNANPNVDPNANPNVDPNANPNKNNQGNGQGHNMPNDPNRNVDENANANSAVKNNNNEEPSDKHIKEYLNKIQNSLSTEWSPCSVTCGNGIQVRIKPGSANKPKDELDYANDIEKKICKMEKCSSVRTMLEGGSGGDGGSGGDEEAELAYLLGELAYKLGEYRIAIRAYRIALKRDPNNAEAWYNLGNAYYKQGRYREAIEYYQKALELDPNNAEAWYNLGNAYYERGEYEEAIEYYRKALRLDPNNADAMQNLLNAKMREEHHHHHH
CSP-Q3	I53-dn5	CSP-Q N-terminal Fusion to I53-dn5B	Yes	Degrades	No	N/A	MSSNSKMDPSLGENDDGNNEDNEKLRKPKHKKLKQPADGNPDPNANPNVDPNANPNVDPNANPNVDPNANPNANPNANPNANPNKNNQGNGQGHNMPNDPNRNVDENANANSAVKNNNNEEPSDKHIKEYLNKIQNSLSTEWSPCSVTCGNGIQVRIKPGSANKPKDELDYANDIEKKICKMEKCSSVRTMLEGGSGGDGGSGGDEEAELAYLLGELAYKLGEYRIAIRAYRIALKRDPNNAEAWYNLGNAYYKQGRYREAIEYYQKALELDPNNAEAWYNLGNAYYERGEYEEAIEYYRKALRLDPNNADAMQNLLNAKMREEHHHHHH
CSP-R3	I53-dn5	CSP-R N-terminal Fusion to I53-dn5B	Yes	Degrades	No	N/A	MSSNSKMDPSLGENDDGNNEDNEKLRKPKHKKLKQPADGNPDPNANPNVDPNANPNVDPNANPNVDPNANPNANPNANPNANPNANPNANPNANPNKNNQGNGQGHNMPNDPNRNVDENANANSAVKNNNNEEPSDKHIKEYLNKIQNSLSTEWSPCSVTCGNGIQVRIKPGSANKPKDELDYANDIEKKICKMEKCSSVRTMLEGGSGGDGGSGGDEEAELAYLLGELAYKLGEYRIAIRAYRIALKRDPNNAEAWYNLGNAYYKQGRYREAIEYYQKALELDPNNAEAWYNLGNAYYERGEYEEAIEYYRKALRLDPNNADAMQNLLNAKMREEHHHHHH
CSP-S	I53-50	C-term on I53-50A	Yes	N/A	Yes	Not used	MNKNNQGNGQGHNMPNDPNRNVDENANANSAVKNNNNEEPSDKHIKEYLNKIQNSLSTEWSPCSVTCGNGIQVRIKPGSANKPKDELDYANDIEKKICKMEKCSSVRTMKMEELFKKHKIVAVLRANSVEEAIEKAVAVFAGGVHLIEITFTVPDADTVIKALSVLKEKGAIIGAGTVTSVEQCRKAVESGAEFIVSPHLDEEISQFCKEKGVFYMPGVMTPTELVKAMKLGHTILKLFPGEVVGPQFVKAMKGPFPNVKFVPTGGVNLDNVCEWFKAGVLAVGVGSALVKGTPDEVREKAKAFVEKIRGCTEGSHHHHHH
CSP-T	I3-01	18 NANP repeats and C-term on I301	No	Expression	No	N/A	MSSNSKMDPNANPNANPNANPNANPNANPNANPNANPNANPNANPNANPNANPNANPNANPNANPNANPNANPNANPNKNNQGNGQGHNMPNDPNRNVDENANANSAVKNNNNEEPSDKHIKEYLNKIQNSLSTEWSPCSVTCGNGIQVRIKPGSANKPKDELDYANDIEKKICKMEKCSSVRTKMEELFKKHKIVAVLRANSVEEAKKKALAVFLGGVDLIEITFTVPDADTVIKELSFLKEMGAIIGAGTVTSVEQCRKAVESGAEFIVSPHLDEEISQF

							CKEKGVFYMPGVMTPTELVKAMKLGHTILKLFPGEVVGPQFVKAMKGPFPNVKFVPTGGVNLDNVCEWFKAGVQAVGVGSALVKGTPVEVAEKAKAFVEKIRGCTEGSHHHHHH
CSP-T2	Supercharged I3-01	18 NANP repeats and C-term on I301 supercharged	No	Expression	No	N/A	MSSNSKMDPNANPNANPNANPNANPNANPNANPNANPNANPNANPNANPNANPNANPNANPNANPNANPNANPNANPNKNNQGNGQGHNMPNDPNRNVDENANANSAVKNNNNEEPSDKHIKEYLNKIQNSLSTEWSPCSVTCGNGIQVRIKPGSANKPKDELDYANDIEKKICKMEKCSSVRTMEELFKEHKIVAVLRANSVEEAKKKALAVFLGGVDLIEITFTVPDADTVIKELSFLKEMGAIIGAGTVTSVEQAREAVESGAEFIVSPHLDEEISQFAKEEGVFYMPGVMTPTELVKAMKLGHTILKLFPGEVVGPQFVEAMKGPFPNVKFVPTGGVNLDNVAEWFEAGVQAVGVGSALVEGTPVEVAEKAKAFVEKIEAATGSHHHHHH
**CSP-U**	I53-50	17 NPDP repeats + NANP + C-term on I53-50A	Yes	N/A	Yes	Figure 3	MSSNSKMDPNPDPNPDPNPDPNPDPNPDPNPDPNPDPNPDPNPDPNPDPNPDPNPDPNPDPNPDPNPDPNPDPNANPNKNNQGNGQGHNMPNDPNRNVDENANANSAVKNNNNEEPSDKHIKEYLNKIQNSLSTEWSPCSVTCGNGIQVRIKPGSANKPKDELDYANDIEKKICKMEKCSSVRTKMEELFKKHKIVAVLRANSVEEAIEKAVAVFAGGVHLIEITFTVPDADTVIKALSVLKEKGAIIGAGTVTSVEQCRKAVESGAEFIVSPHLDEEISQFCKEKGVFYMPGVMTPTELVKAMKLGHTILKLFPGEVVGPQFVKAMKGPFPNVKFVPTGGVNLDNVCEWFKAGVLAVGVGSALVKGTPDEVREKAKAFVEKIRGCTEGSHHHHHH
**CSP-V**	I53-50	17 NVDP repeats + NANP + C-term on I53-50A	Yes	N/A	Yes	Figure 3	MSSNSKMDPNVDPNVDPNVDPNVDPNVDPNVDPNVDPNVDPNVDPNVDPNVDPNVDPNVDPNVDPNVDPNVDPNVDPNANPNKNNQGNGQGHNMPNDPNRNVDENANANSAVKNNNNEEPSDKHIKEYLNKIQNSLSTEWSPCSVTCGNGIQVRIKPGSANKPKDELDYANDIEKKICKMEKCSSVRTKMEELFKKHKIVAVLRANSVEEAIEKAVAVFAGGVHLIEITFTVPDADTVIKALSVLKEKGAIIGAGTVTSVEQCRKAVESGAEFIVSPHLDEEISQFCKEKGVFYMPGVMTPTELVKAMKLGHTILKLFPGEVVGPQFVKAMKGPFPNVKFVPTGGVNLDNVCEWFKAGVLAVGVGSALVKGTPDEVREKAKAFVEKIRGCTEGSHHHHHH
**CSP-W**	I53-50	9 sets of alternating NPDP + NANP repeats + C-term on I53-50A	Yes	N/A	Yes	Figure 3	MSSNSKMDPNPDPNANPNPDPNANPNPDPNANPNPDPNANPNPDPNANPNPDPNANPNPDPNANPNPDPNANPNPDPNANPNKNNQGNGQGHNMPNDPNRNVDENANANSAVKNNNNEEPSDKHIKEYLNKIQNSLSTEWSPCSVTCGNGIQVRIKPGSANKPKDELDYANDIEKKICKMEKCSSVRTKMEELFKKHKIVAVLRANSVEEAIEKAVAVFAGGVHLIEITFTVPDADTVIKALSVLKEKGAIIGAGTVTSVEQCRKAVESGAEFIVSPHLDEEISQFCKEKGVFYMPGVMTPTELVKAMKLGHTILKLFPGEVVGPQFVKA

							MKGPFPNVKFVPTGGVNLDNV<br>CEWFKAGVLAVGVGSALVKGTP<br>DEVREKAKAFVEKIRGCTEGSH<br>HHHHH
**CSP-Y**	I53-50	9 sets of alternating NVDP + NANP repeats + C-term on I53-50A	Yes	N/A	Yes	Figure 3	MSSNSKMDPNPDPNANPNVDP<br>NANPNPDPNANPNVDPNANPN<br>PDPNANPNVDPNANPNPDPNA<br>NPNVDPNANPNPDPNANPNKN<br>NQGNGQGHNMPNDPNRNVDE<br>NANANSAVKNNNNEEPSDKHIK<br>EYLNKIQNSLSTEWSPCSVTCG<br>NGIQVRIKPGSANKPKDELDYA<br>NDIEKKICKMEKCSSVRTKMEEL<br>FKKHKIVAVLRANSVEEAIEKAVA<br>VFAGGVHLIEITFTVPDADTVIKA<br>LSVLKEKGAIIGAGTVTSVEQCR<br>KAVESGAEFIVSPHLDEEISQFC<br>KEKGVFYMPGVMTPTELVKAM<br>KLGHTILKLFPGEVVGPQFVKA<br>MKGPFPNVKFVPTGGVNLDNV<br>CEWFKAGVLAVGVGSALVKGTP<br>DEVREKAKAFVEKIRGCTEGSH<br>HHHHH
**CSP-X**	I53-50	18 repeats NPDPNANPNVDPNANP + C-term on I53-50A	Yes	N/A	Yes	Figure 3	MSSNSKMDPNVDPNANPNVDP<br>NANPNVDPNANPNVDPNANPN<br>VDPNANPNVDPNANPNVDPNA<br>NPNVDPNANPNVDPNANPNKN<br>NQGNGQGHNMPNDPNRNVDE<br>NANANSAVKNNNNEEPSDKHIK<br>EYLNKIQNSLSTEWSPCSVTCG<br>NGIQVRIKPGSANKPKDELDYA<br>NDIEKKICKMEKCSSVRTKMEEL<br>FKKHKIVAVLRANSVEEAIEKAVA<br>VFAGGVHLIEITFTVPDADTVIKA<br>LSVLKEKGAIIGAGTVTSVEQCR<br>KAVESGAEFIVSPHLDEEISQFC<br>KEKGVFYMPGVMTPTELVKAM<br>KLGHTILKLFPGEVVGPQFVKA<br>MKGPFPNVKFVPTGGVNLDNV<br>CEWFKAGVLAVGVGSALVKGTP<br>DEVREKAKAFVEKIRGCTEGSH<br>HHHHH
**CSP-Z**	I53-50	Two junctional regions and C-term on I53-50A	Yes	N/A	Yes	Figure 3	MSSNSKMDPNPDPNANPNVDP<br>NANPNVDPNANPNVDPNANPN<br>PDPNANPNVDPNANPNVDPNA<br>NPNVDPNANPNPDPNANPNKN<br>NQGNGQGHNMPNDPNRNVDE<br>NANANSAVKNNNNEEPSDKHIK<br>EYLNKIQNSLSTEWSPCSVTCG<br>NGIQVRIKPGSANKPKDELDYA<br>NDIEKKICKMEKCSSVRTKMEEL<br>FKKHKIVAVLRANSVEEAIEKAVA<br>VFAGGVHLIEITFTVPDADTVIKA<br>LSVLKEKGAIIGAGTVTSVEQCR<br>KAVESGAEFIVSPHLDEEISQFC<br>KEKGVFYMPGVMTPTELVKAM<br>KLGHTILKLFPGEVVGPQFVKA<br>MKGPFPNVKFVPTGGVNLDNV<br>CEWFKAGVLAVGVGSALVKGTP<br>DEVREKAKAFVEKIRGCTEGSH<br>HHHHH
CSP-α	I53-50	Alternating NPDP and NVDP repeats +2XNANP + C-term on I53-50A	Yes	N/A	No	N/A	MSSNSKMDPNPDPNVDPNPDP<br>NVDPNPDPNVDPNPDPNVDPN<br>PDPNVDPNPDPNVDPNPDPNV<br>DPNPDPNVDPNPDPNANPNKN<br>NQGNGQGHNMPNDPNRNVDE<br>NANANSAVKNNNNEEPSDKHIK<br>EYLNKIQNSLSTEWSPCSVTCG<br>NGIQVRIKPGSANKPKDELDYA<br>NDIEKKICKMEKCSSVRTKMEEL<br>FKKHKIVAVLRANSVEEAIEKAVA<br>VFAGGVHLIEITFTVPDADTVIKA<br>LSVLKEKGAIIGAGTVTSVEQCR<br>KAVESGAEFIVSPHLDEEISQFC<br>KEKGVFYMPGVMTPTELVKAM<br>KLGHTILKLFPGEVVGPQFVKA<br>MKGPFPNVKFVPTGGVNLDNV<br>CEWFKAGVLAVGVGSALVKGTP

							DEVREKAKAFVEKIRGCTEGSH<br>HHHHH
**CSP-β**	I53-50	6 sets of NPDPNVDPNANP + C-term on I53-50A	Yes	N/A	Yes	Figure 3	MSSNSKMDPNPDPNVDPNANP<br>NPDPNVDPNANPNPDPNVDPN<br>ANPNPDPNVDPNANPNPDPNV<br>DPNANPNPDPNVDPNANPNKN<br>NQGNGQGHNMPNDPNRNVDE<br>NANANSAVKNNNNEEPSDKHIK<br>EYLNKIQNSLSTEWSPCSVTCG<br>NGIQVRIKPGSANKPKDELDYA<br>NDIEKKICKMEKCSSVRTKMEEL<br>FKKHKIVAVLRANSVEEAIEKAVA<br>VFAGGVHLIEITFTVPDADTVIKA<br>LSVLKEKGAIIGAGTVTSVEQCR<br>KAVESGAEFIVSPHLDEEISQFC<br>KEKGVFYMPGVMTPTELVKAM<br>KLGHTILKLFPGEVVGPQFVKA<br>MKGPFPNVKFVPTGGVNLDNV<br>CEWFKAGVLAVGVGSALVKGTP<br>DEVREKAKAFVEKIRGCTEGSH<br>HHHHH
CSP-γ	I53-dn5	CSP-U on I53-dn5B	Yes	Degrades	No	N/A	MSSNSKMDPNPDPNPDPNPDP<br>NPDPNPDPNPDPNPDPNPDPN<br>PDPNPDPNPDPNPDPNPDPNP<br>DPNPDPNPDPNPDPNANPNKN<br>NQGNGQGHNMPNDPNRNVDE<br>NANANSAVKNNNNEEPSDKHIK<br>EYLNKIQNSLSTEWSPCSVTCG<br>NGIQVRIKPGSANKPKDELDYA<br>NDIEKKICKMEKCSSVRTGGSG<br>GDGGSGGDEEAELAYLLGELAY<br>KLGEYRIAIRAYRIALKRDPNNA<br>EAWYNLGNAYYKQGRYREAIEY<br>YQKALELDPNNAEAWYNLGNA<br>YYERGEYEEAIEYYRKALRLDP<br>NNADAMQNLLNAKMREEHHHH<br>HH
CSP-δ	I53-dn5	CSP-V on I53-dn5B	Yes	Degrades	No	N/A	MSSNSKMDPNVDPNVDPNVDP<br>NVDPNVDPNVDPNVDPNVDPN<br>VDPNVDPNVDPNVDPNVDPNV<br>DPNVDPNVDPNVDPNANPNKN<br>NQGNGQGHNMPNDPNRNVDE<br>NANANSAVKNNNNEEPSDKHIK<br>EYLNKIQNSLSTEWSPCSVTCG<br>NGIQVRIKPGSANKPKDELDYA<br>NDIEKKICKMEKCSSVRTGGSG<br>GDGGSGGDEEAELAYLLGELAY<br>KLGEYRIAIRAYRIALKRDPNNA<br>EAWYNLGNAYYKQGRYREAIEY<br>YQKALELDPNNAEAWYNLGNA<br>YYERGEYEEAIEYYRKALRLDP<br>NNADAMQNLLNAKMREEHHHH<br>HH
CSP-ε	I53-dn5	CSP-W on I53-dn5B	Yes	Degrades	No	N/A	MSSNSKMDPNPDPNANPNPDP<br>NANPNPDPNANPNPDPNANPN<br>PDPNANPNPDPNANPNPDPNA<br>NPNPDPNANPNPDPNANPNKN<br>NQGNGQGHNMPNDPNRNVDE<br>NANANSAVKNNNNEEPSDKHIK<br>EYLNKIQNSLSTEWSPCSVTCG<br>NGIQVRIKPGSANKPKDELDYA<br>NDIEKKICKMEKCSSVRTGGSG<br>GDGGSGGDEEAELAYLLGELAY<br>KLGEYRIAIRAYRIALKRDPNNA<br>EAWYNLGNAYYKQGRYREAIEY<br>YQKALELDPNNAEAWYNLGNA<br>YYERGEYEEAIEYYRKALRLDP<br>NNADAMQNLLNAKMREEHHHH<br>HH
CSP-ζ	I53-dn5	CSP-X on I53-dn5B	Yes	Degrades	No	N/A	MSSNSKMDPNVDPNANPNVDP<br>NANPNVDPNANPNVDPNANPN<br>VDPNANPNVDPNANPNVDPNA<br>NPNVDPNANPNVDPNANPNKN<br>NQGNGQGHNMPNDPNRNVDE<br>NANANSAVKNNNNEEPSDKHIK<br>EYLNKIQNSLSTEWSPCSVTCG<br>NGIQVRIKPGSANKPKDELDYA

							NDIEKKICKMEKCSSVRTGGSG<br>GDGGSGGDEEAELAYLLGELAY<br>KLGEYRIAIRAYRIALKRDPNNA<br>EAWYNLGNAYYKQGRYREAIEY<br>YQKALELDPNNAEAWYNLGNA<br>YYERGEYEEAIEYYRKALRLDP<br>NNADAMQNLLNAKMREEHHHH<br>HH
CSP-η	I53-dn5	CSP-Y on I53-dn5B	Yes	Degrades	No	N/A	MSSNSKMDPNPDPNANPNVDP<br>NANPNPDPNANPNVDPNANPN<br>PDPNANPNVDPNANPNPDPNA<br>NPNVDPNANPNPDPNANPNKN<br>NQGNGQGHNMPNDPNRNVDE<br>NANANSAVKNNNNEEPSDKHIK<br>EYLNKIQNSLSTEWSPCSVTCG<br>NGIQVRIKPGSANKPKDELDYA<br>NDIEKKICKMEKCSSVRTGGSG<br>GDGGSGGDEEAELAYLLGELAY<br>KLGEYRIAIRAYRIALKRDPNNA<br>EAWYNLGNAYYKQGRYREAIEY<br>YQKALELDPNNAEAWYNLGNA<br>YYERGEYEEAIEYYRKALRLDP<br>NNADAMQNLLNAKMREEHHHH<br>HH
CSP-θ	I53-dn5	CSP-Z on I53-dn5B	Yes	Degrades	No	N/A	MSSNSKMDPNPDPNANPNVDP<br>NANPNVDPNANPNVDPNANPN<br>PDPNANPNVDPNANPNVDPNA<br>NPNVDPNANPNPDPNANPNKN<br>NQGNGQGHNMPNDPNRNVDE<br>NANANSAVKNNNNEEPSDKHIK<br>EYLNKIQNSLSTEWSPCSVTCG<br>NGIQVRIKPGSANKPKDELDYA<br>NDIEKKICKMEKCSSVRTGGSG<br>GDGGSGGDEEAELAYLLGELAY<br>KLGEYRIAIRAYRIALKRDPNNA<br>EAWYNLGNAYYKQGRYREAIEY<br>YQKALELDPNNAEAWYNLGNA<br>YYERGEYEEAIEYYRKALRLDP<br>NNADAMQNLLNAKMREEHHHH<br>HH
CSP-λ	I53-dn5	CSP-α on I53-dn5B	Yes	Degrades	No	N/A	MSSNSKMDPNPDPNVDPNPDP<br>NVDPNPDPNVDPNPDPNVDPN<br>PDPNVDPNPDPNVDPNPDPNV<br>DPNPDPNVDPNPDPNANPNKN<br>NQGNGQGHNMPNDPNRNVDE<br>NANANSAVKNNNNEEPSDKHIK<br>EYLNKIQNSLSTEWSPCSVTCG<br>NGIQVRIKPGSANKPKDELDYA<br>NDIEKKICKMEKCSSVRTGGSG<br>GDGGSGGDEEAELAYLLGELAY<br>KLGEYRIAIRAYRIALKRDPNNA<br>EAWYNLGNAYYKQGRYREAIEY<br>YQKALELDPNNAEAWYNLGNA<br>YYERGEYEEAIEYYRKALRLDP<br>NNADAMQNLLNAKMREEHHHH<br>HH
CSP-μ	I53-dn5	CSP-β on I53-dn5B	Yes	Degrades	No	N/A	MSSNSKMDPNPDPNVDPNANP<br>NPDPNVDPNANPNPDPNVDPN<br>ANPNPDPNVDPNANPNPDPNV<br>DPNANPNPDPNVDPNANPNKN<br>NQGNGQGHNMPNDPNRNVDE<br>NANANSAVKNNNNEEPSDKHIK<br>EYLNKIQNSLSTEWSPCSVTCG<br>NGIQVRIKPGSANKPKDELDYA<br>NDIEKKICKMEKCSSVRTGGSG<br>GDGGSGGDEEAELAYLLGELAY<br>KLGEYRIAIRAYRIALKRDPNNA<br>EAWYNLGNAYYKQGRYREAIEY<br>YQKALELDPNNAEAWYNLGNA<br>YYERGEYEEAIEYYRKALRLDP<br>NNADAMQNLLNAKMREEHHHH<br>HH
CSP X/Z Mosaic	I53-50	CSP-X + CSP-Z 50% valency on I53-50A	Yes	N/A	Yes	Figure 4	Refer to CSP X, CSP Z
CSP X/RT Mosaic	I53-50	CSP-X + RT 50% valency on I53-50A	Yes	N/A	Yes	Figure 4	Refer to CSP X, RT-I53-50A

CSP Z/RT Mosaic	I53-50	CSP-Z + RT 50% valency on I53-50A	Yes	N/A	Yes	Figure 4	Refer to CSP Z, RT-I53-50A
CSP X/Z/RT Mosaic	I53-50	CSP-X + CSP-Z + RT 33% valency on I53-50A	Yes	N/A	Yes	Figure 4	Refer to CSP X, CSP Z, RT-I53-50A
CSP π	I53-dn5	Natural CSP repeats without C term on I53_dn5B	Yes	Degrades	No	N/A	MSSNSKMDPNPDPNANPNVDPNANPNVDPNANPNVDPNANPNPDPNANPNVDPNANPNVDPNANPNVDPNANPNPDPNANPCSSVRTGGSGGDGGSGGDEEAELAYLLGELAYKLGEYRIAIRAYRIALKRDPNNAEAWYNLGNAYYKQGRYREAIEYYQKALELDPNNAEAWYNLGNAYYERGEYEEAIEYYRKALRLDPNNADAMQNLLNAKMREEHHHHHH
CSP ρ	I53-dn5	CSP F on I53_dn5B	No	Expression	No	N/A	MSLGENDDGNNEDNEKLRKPKHKKLKQPADGNPDPNANPNVDPNANPNVDPNANPNVDPNANPNANPNANPNANPNANPNANPNANPNANPNANPNANPNANPNANPNANPNANPNANPNANPNVDPNANPNANPNANPEKAAKAEEAARGGSGGDGGSGGDEEAELAYLLGELAYKLGEYRIAIRAYRIALKRDPNNAEAWYNLGNAYYKQGRYREAIEYYQKALELDPNNAEAWYNLGNAYYERGEYEEAIEYYRKALRLDPNNADAMQNLLNAKMREEHHHHHH
CSP ξ	I53-50	Natural CSP repeats without C term on I53-50A	Yes	Degrades	No	N/A	MSSNSKMDPNPDPNANPNVDPNANPNVDPNANPNVDPNANPNPDPNANPNVDPNANPNVDPNANPNVDPNANPNPDPNANPCSSVRTGSGSKMEELFKKHKIVAVLRANSVEEAIEKAVAVFAGGVHLIEITFTVPDADTVIKALSVLKEKGAIIGAGTVTSVEQCRKAVESGAEFIVSPHLDEEISQFCKEKGVFYMPGVMTPTELVKAMKLGHTILKLFPGEVVGPQFVKAMKGPFPNVKFVPTGGVNLDNVCEWFKAGVLAVGVGSALVKGTPDEVREKAKAFVEKIRGCTEGSHHHHHH
CSP Σ	I53-dn5	C term only on I53_dn5B	Yes	Degrades	No	N/A	MNKNNQGNGQGHNMPNDPNRNVDENANANSAVKNNNNEEPSDKHIKEYLNKIQNSLSTEWSPCSVTCGNGIQVRIKPGSANKPKDELDYANDIEKKICKMEKCSSVRTGGSGGDGGSGGDEEAELAYLLGELAYKLGEYRIAIRAYRIALKRDPNNAEAWYNLGNAYYKQGRYREAIEYYQKALELDPNNAEAWYNLGNAYYERGEYEEAIEYYRKALRLDPNNADAMQNLLNAKMREEHHHHHH
RT-I53_dn5B	I53-dn5	RT on I53-dn5B	Yes	Degrades	No	N/A	MSSNSKMDPNANPNANPNANPNANPNANPNANPNANPNANPNANPNANPNANPNANPNANPNANPNANPNANPNANPNKNNQGNGQGHNMPNDPNRNVDENANANSAVKNNNNEEPSDKHIKEYLNKIQNSLSTEWSPCSVTCGNGIQVRIKPGSANKPKDELDYANDIEKKICKMEKCSSVRTGGSGGDGGSGGDEEAELAYLLGELAYKLGEYRIAIRAYRIALKRDPNNAEAWYNLGNAYYKQGRYREAIEYYQKALELDPNNAEAWYNLGNAYYERGEYEEAIEYYRKALRLDPNNADAMQNLLNAKMREEHHHHHH

CSP-Psi cp 7	I53-dn5 cp7	CSP-Z on dn5A cp7 N-term	No	Degrades	No	N/A	MSSNSKMDPNPDPNANPNVDPNANPNVDPNANPNVDPNANPNPDPNANPNVDPNANPNVDPNANPNVDPNANPNPDPNANPNKNNQGNGQGHNMPNDPNRNVDENANANSAVKNNNNEEPSDKHIKEYLNKIQNSLSTEWSPCSVTCGNGIQVRIKPGSANKPKDELDYANDIEKKICKMEKCSSVRTGSGSDEQAEERAGTKAGNHGEDWGAAAVEMATKFNGDGGSGKYDGSKLRIGILHARGNAEIILELVLGALKRLQEFGVKRENIIIETVPGSFELPYGSKLFVEKQKRLGKPLDAIIPIGVLIRGSTAHFDYIADSTTHQLMKLNFELGIPVIFGVLTTESGGLEHHHHHH
CSP-Psi cp 6	I53-dn5 cp6	CSP-Z on dn5A cp6 N-term	No	Degrades	No	N/A	MSSNSKMDPNPDPNANPNVDPNANPNVDPNANPNPDPNANPNVDPNANPNVDPNANPNVDPNANPNPDPNANPNKNNQGNGQGHNMPNDPNRNVDENANANSAVKNNNNEEPSDKHIKEYLNKIQNSLSTEWSPCSVTCGNGIQVRIKPGSANKPKDELDYANDIEKKICKMEKCSSVRTGSGSDEQAEERAGTKAGNHGEDWGAAAVEMATKFNGDGGKYDGSKLRIGILHARGNAEIILELVLGALKRLQEFGVKRENIIIETVPGSFELPYGSKLFVEKQKRLGKPLDAIIPIGVLIRGSTAHFDYIADSTTHQLMKLNFELGIPVIFGVLTTESGGLEHHHHH
CSP-Phi cp 7	I53-dn5 cp7	CSP-Z on dn5A cp7 N,C-term **(Just C_term?)**	Yes	Degrades	No	N/A	MDEQAEERAGTKAGNHGEDWGAAAVEMATKFNGDGGSGKYDGSKLRIGILHARGNAEIILELVLGALKRLQEFGVKRENIIIETVPGSFELPYGSKLFVEKQKRLGKPLDAIIPIGVLIRGSTAHFDYIADSTTHQLMKLNFELGIPVIFGVLTTESGGLEGGSGGDSSNSKMDPNPDPNANPNVDPNANPNVDPNANPNVDPNANPNPDPNANPNVDPNANPNVDPNANPNVDPNANPNPDPNANPGSGSHHHHHH
CSP-Phi cp 6	I53-dn5 cp6	CSP-Z on dn5A cp6 N,C-term **(Just C-term?)**	Yes	Degrades	No	N/A	MDEQAEERAGTKAGNHGEDWGAAAVEMATKFNGDGGKYDGSKLRIGILHARGNAEIILELVLGALKRLQEFGVKRENIIIETVPGSFELPYGSKLFVEKQKRLGKPLDAIIPIGVLIRGSTAHFDYIADSTTHQLMKLNFELGIPVIFGVLTTESGGLEGGSGGDSSNSKMDPNPDPNANPNVDPNANPNVDPNANPNVDPNANPNPDPNANPNVDPNANPNVDPNANPNPDPNANPGSGSHHHHHH
RT_cp 6	I53-dn5 cp6	RT on N-term of dn5A cp6	Yes	Doesn't Assemble	No	N/A	MSSNSKMDPNANPNANPNANPNANPNANPNANPNANPNANPNANPNANPNANPNANPNANPNANPNANPNANPNANPNKNNQGNGQGHNMPNDPNRNVDENANANSAVKNNNNEEPSDKHIKEYLNKIQNSLSTEWSPCSVTCGNGIQVRIKPGSANKPKDELDYANDIEKKICKMEKCSSVRTGGSGGDGGSGGDDEQAEERAGTKAGNHGEDWGAAAVEMATKFNGDGGKYDGSKLRIGILHARGNAEIILELVLGALKRLQEFGVKRENIIIETVPGSFELPYGSKLFVEKQKRLGKPLDAIIPIGVLIRGSTAHFDYIADSTTHQLMKLNFELGIPVIFGVLTTESGGLEHHHHHH

RT_ cp7	I53-dn5 cp7	RT on N-term of dn5B cp7	Yes	Doesn't Assemble	No	N/A	MSSNSKMDPNANPNANPNANPNANPNANPNANPNANPNANPNANPNANPNANPNANPNANPNANPNANPNANPNANPNKNNQGNGQGHNMPNDPNRNVDENANANSAVKNNNNEEPSDKHIKEYLNKIQNSLSTEWSPCSVTCGNGIQVRIKPGSANKPKDELDYANDIEKKICKMEKCSSVRTGGSGGDGGSGGDDEQAEERAGTKAGNHGEDWGAAAVEMATKFNGDGGSGKYDGSKLRIGILHARGNAEIILELVLGALKRLQEFGVKRENIIIETVPGSFELPYGSKLFVEKQKRLGKPLDAIIPIGVLIRGSTAHFDYIADSTTHQLMKLNFELGIPVIFGVLTTESGGLEHHHHHH
cp 6_RT	I53-dn5 cp6	RT on C-term of dn5B cp6	No		No	N/A	MDEQAEERAGTKAGNHGEDWGAAAVEMATKFNGDGGKYDGSKLRIGILHARGNAEIILELVLGALKRLQEFGVKRENIIIETVPGSFELPYGSKLFVEKQKRLGKPLDAIIPIGVLIRGSTAHFDYIADSTTHQLMKLNFELGIPVIFGVLTTESGGLEGGSGGDGGSGGDSSNSKMDPNANPNANPNANPNANPNANPNANPNANPNANPNANPNANPNANPNANPNANPNANPNANPNANPNKNNQGNGQGHNMPNDPNRNVDENANANSAVKNNNNEEPSDKHIKEYLNKIQNSLSTEWSPCSVTCGNGIQVRIKPGSANKPKDELDYANDIEKKICKMEKCSSVRTHHHHHH
cp 7_RT	I53-dn5 cp7	RT on C-term of dn5B cp7	No		No	N/A	MDEQAEERAGTKAGNHGEDWGAAAVEMATKFNGDGGSGKYDGSKLRIGILHARGNAEIILELVLGALKRLQEFGVKRENIIIETVPGSFELPYGSKLFVEKQKRLGKPLDAIIPIGVLIRGSTAHFDYIADSTTHQLMKLNFELGIPVIFGVLTTESGGLEGGSGGDGGSGGDSSNSKMDPNANPNANPNANPNANPNANPNANPNANPNANPNANPNANPNANPNANPNANPNANPNANPNANPNKNNQGNGQGHNMPNDPNRNVDENANANSAVKNNNNEEPSDKHIKEYLNKIQNSLSTEWSPCSVTCGNGIQVRIKPGSANKPKDELDYANDIEKKICKMEKCSSVRTHHHHHH
RT_cp6_RT	I53-dn5 cp6	RT on N, C-term of dn5B cp6	Yes		No	N/A	MSSNSKMDPNANPNANPNANPNANPNANPNANPNANPNANPNANPNANPNANPNANPNANPNANPNANPNANPNANPNKNNQGNGQGHNMPNDPNRNVDENANANSAVKNNNNEEPSDKHIKEYLNKIQNSLSTEWSPCSVTCGNGIQVRIKPGSANKPKDELDYANDIEKKICKMEKCSSVRTGGSGGDGGSGGDDEQAEERAGTKAGNHGEDWGAAAVEMATKFNGDGGKYDGSKLRIGILHARGNAEIILELVLGALKRLQEFGVKRENIIIETVPGSFELPYGSKLFVEKQKRLGKPLDAIIPIGVLIRGSTAHFDYIADSTTHQLMKLNFELGIPVIFGVLTTESGGLEGGSGGDGGSGGDSSNSKMDPNANPNANPNANPNANPNANPNANPNANPNANPNANPNANPNANPNANPNANPNANPNANPNANPNKNNQGNGQGHNMPNDPNRNVDENANANSAVKNNNNEEPSDKHIKEYLNKIQNSLSTEWSPCSVTCGNGIQVRIKPGSANKPKDELDYANDIEKKICKMEKCSSVRTHHHHHH

RT_cp7_RT	I53-dn5 cp7	RT on N, C-term of dn5B cp7	Yes		No	N/A	MSSNSKMDPNANPNANPNANPNANPNANPNANPNANPNANPNANPNANPNANPNANPNANPNANPNANPNANPNANPNANPNKNNQGNGQGHNMPNDPNRNVDENANANSAVKNNNNEEPSDKHIKEYLNKIQNSLSTEWSPCSVTCGNGIQVRIKPGSANKPKDELDYANDIEKKICKMEKCSSVRTGGSGGDGGSGGDDEQAEERAGTKAGNHGEDWGAAAVEMATKFNGDGGSGKYDGSKLRIGILHARGNAEIILELVLGALKRLQEFGVKRENIIIETVPGSFELPYGSKLFVEKQKRLGKPLDAIIPIGVLIRGSTAHFDYIADSTTHQLMKLNFELGIPVIFGVLTTESGGLEGGSGGDGGSGGDSSNSKMDPNANPNANPNANPNANPNANPNANPNANPNANPNANPNANPNANPNANPNANPNANPNANPNANPNANPNKNNQGNGQGHNMPNDPNRNVDENANANSAVKNNNNEEPSDKHIKEYLNKIQNSLSTEWSPCSVTCGNGIQVRIKPGSANKPKDELDYANDIEKKICKMEKCSSVRTHHHHHH
CSP-Omega cp 7	I53-dn5 cp7	CSP-Z on dn5A C-term	Yes		No	N/A	MSSNSKMDPNPDPNANPNVDPNANPNVDPNANPNVDPNANPNPDPNANPNVDPNANPNVDPNANPNVDPNANPNPDPNANPNKNNQGNGQGHNMPNDPNRNVDENANANSAVKNNNNEEPSDKHIKEYLNKIQNSLSTEWSPCSVTCGNGIQVRIKPGSANKPKDELDYANDIEKKICKMEKCSSVRTGSGSDEQAEERAGTKAGNHGEDWGAAAVEMATKFNGDGGSGKYDGSKLRIGILHARGNAEIILELVLGALKRLQEFGVKRENIIIETVPGSFELPYGSKLFVEKQKRLGKPLDAIIPIGVLIRGSTAHFDYIADSTTHQLMKLNFELGIPVIFGVLTTESGGLEGGSGGDSSNSKMDPNPDPNANPNVDPNANPNVDPNANPNVDPNANPNPDPNANPNVDPNANPNVDPNANPNVDPNANPNPDPNANPNKNNQGNGQGHNMPNDPNRNVDENANANSAVKNNNNEEPSDKHIKEYLNKIQNSLSTEWSPCSVTCGNGIQVRIKPGSANKPKDELDYANDIEKKICKMEKCSSVRTHHHHHH
CSP-Omega cp 6	I53-dn5 cp6	CSP-Z on dn5A C-term	Yes		No	N/A	MSSNSKMDPNPDPNANPNVDPNANPNVDPNANPNVDPNANPNPDPNANPNVDPNANPNVDPNANPNVDPNANPNPDPNANPNKNNQGNGQGHNMPNDPNRNVDENANANSAVKNNNNEEPSDKHIKEYLNKIQNSLSTEWSPCSVTCGNGIQVRIKPGSANKPKDELDYANDIEKKICKMEKCSSVRTGSGSDEQAEERAGTKAGNHGEDWGAAAVEMATKFNGDGGKYDGSKLRIGILHARGNAEIILELVLGALKRLQEFGVKRENIIIETVPGSFELPYGSKLFVEKQKRLGKPLDAIIPIGVLIRGSTAHFDYIADSTTHQLMKLNFELGIPVIFGVLTTESGGLEGGSGGDSSNSKMDPNPDPNANPNVDPNANPNVDPNANPNVDPNANPNPDPNANPNVDPNANPNVDPNANPNVDPNANPNPDPNANPNKNNQGNGQGHNMPNDPNRNVDENANANSAVKNNNNEEPSDKHIKEYLNKIQNSLSTEWSPCSVTCGNGIQVRIKPGSANKPKDELDYANDIEKKICKMEKCSSVRTHHHHHH

CSP-Z-PADRE	I53-50	CSP-Z on I53-50A with PADRE peptide on C-term	Yes	Aggregates	No	N/A	MSSNSKMDPNPDPNANPNVDP<br>NANPNVDPNANPNVDPNANPN<br>PDPNANPNVDPNANPNVDPNA<br>NPNVDPNANPNPDPNANPNKN<br>NQGNGQGHNMPNDPNRNVDE<br>NANANSAVKNNNNEEPSDKHIK<br>EYLNKIQNSLSTEWSPCSVTCG<br>NGIQVRIKPGSANKPKDELDYA<br>NDIEKKICKMEKCSSVRTKMEEL<br>FKKHKIVAVLRANSVEEAIEKAVA<br>VFAGGVHLIEITFTVPDADTVIKA<br>LSVLKEKGAIIGAGTVTSVEQCR<br>KAVESGAEFIVSPHLDEEISQFC<br>KEKGVFYMPGVMTPTELVKAM<br>KLGHDILKLFPGEVVGPQFVKA<br>MKGPFPNVKFVPTGGVNLDNV<br>CEWFKAGVLAVGVGDALVKGD<br>PDEVREKAKKFVEKIRGCTEGS<br>AKFVAAWTLKAAAGSGSHHHH<br>HH
CSP-Xi-PADRE	I53-50	CSP-Z on I53-dn5B with PADRE peptide on C-term	Yes	Aggregates	No	N/A	MSSNSKMDPNPDPNANPNVDP<br>NANPNVDPNANPNVDPNANPN<br>PDPNANPNVDPNANPNVDPNA<br>NPNVDPNANPNPDPNANPCSS<br>VRTGSGSKMEELFKKHKIVAVL<br>RANSVEEAIEKAVAVFAGGVHLI<br>EITFTVPDADTVIKALSVLKEKG<br>AIIGAGTVTSVEQCRKAVESGAE<br>FIVSPHLDEEISQFCKEKGVFYM<br>PGVMTPTELVKAMKLGHTILKLF<br>PGEVVGPQFVKAMKGPFPNVK<br>FVPTGGVNLDNVCEWFKAGVL<br>AVGVGSALVKGTPDEVREKAKA<br>FVEKIRGCTEGSAKFVAAWTLK<br>AAAGSGSHHHHHH
CSP-2Z-PADRE	I53-50	CSP-ZX2 on I53-50A with PADRE peptide on C-term	Yes	Aggregates	No	N/A	MSSNSKMDPNPDPNANPNVDP<br>NANPNVDPNANPNVDPNANPN<br>PDPNANPNVDPNANPNVDPNA<br>NPNVDPNANPNPDPNANPDPN<br>PDPNANPNVDPNANPNVDPNA<br>NPNVDPNANPNPDPNANPNVD<br>PNANPNVDPNANPNVDPNANP<br>NPDPNANPCSSVRTGSGSKME<br>ELFKKHKIVAVLRANSVEEAIEKA<br>VAVFAGGVHLIEITFTVPDADTVI<br>KALSVLKEKGAIIGAGTVTSVEQ<br>CRKAVESGAEFIVSPHLDEEISQ<br>FCKEKGVFYMPGVMTPTELVKA<br>MKLGHTILKLFPGEVVGPQFVK<br>AMKGPFPNVKFVPTGGVNLDN<br>VCEWFKAGVLAVGVGSALVKGT<br>PDEVREKAKAFVEKIRGCTEGS<br>AKFVAAWTLKAAAGSGSHHHH<br>HH
CSP-2theta-PADRE	I53-dn5	CSP-ZX2 on I53-dn5B with PADRE peptide on C-term	Yes	Aggregates	No	N/A	MSSNSKMDPNPDPNANPNVDP<br>NANPNVDPNANPNVDPNANPN<br>PDPNANPNVDPNANPNVDPNA<br>NPNVDPNANPNPDPNANPDPN<br>PDPNANPNVDPNANPNVDPNA<br>NPNVDPNANPNPDPNANPNVD<br>PNANPNVDPNANPNVDPNANP<br>NPDPNANPCSSVRTGGSGGDG<br>GSGGDEEAELAYLLGELAYKLG<br>EYRIAIRAYRIALKRDPNNAEAW<br>YNLGNAYYKQGRYREAIEYYQK<br>ALELDPNNAEAWYNLGNAYYER<br>GEYEEAIEYYRKALRLDPNNAD<br>AMQNLLNAKMREELEHHHHHH

RT_I53-50A_PADRE	I53-50	PADRE on the C terminus of RT-I53-50A	Yes	Degrades	No	N/A	MSSNSKMDPNANPNANPNANPNANPNANPNANPNANPNANPNANPNANPNANPNANPNANPNANPNANPNANPNANPNKNNQGNGQGHNMPNDPNRNVDENANANSAVKNNNNEEPSDKHIKEYLNKIQNSLSTEWSPCSVTCGNGIQVRIKPGSANKPKDELDYANDIEKKICKMEKCSSVRTKMEELFKKHKIVAVLRANSVEEAIEKAVAVFAGGVHLIEITFTVPDADTVIKALSVLKEKGAIIGAGTVTSVEQCRKAVESGAEFIVSPHLDEEISQFCKEKGVFYMPGVMTPTELVKAMKLGHTILKLFPGEVVGPQFVKAMKGPFPNVKFVPTGGVNLDNVCEWFKAGVLAVGVGSALVKGTPDEVREKAKAFVEKIRGCTELEGSAKFVAAWTLKAAAGSHHHHHH
dn5bL1R3_01	I53-dn5	3 sets of NANP repeats in loop 1 and on N-term of I53-dn5B	Yes		No	N/A	MNANPNPDPNANPNVDPNANPGGSGGDGGSGGDEEAELAYLLGELAYKLGEYRIAIRAYRIALKRGNPNANPNANPNAEAWYNLGNAYYKQGRYREAIEYYQKALELDPNNAEAWYNLGNAYYERGEYEEAIEYYRKALRLDPNNADAMQNLLNAKMREELEHHHHHH
dn5bL1R4_02	I53-dn5	4 sets of NANP repeats in loop 1 and on N-term of I53-dn5B	Yes		No	N/A	MNANPNPDPNANPNVDPNANPGGSGGDGGSGGDEEAELAYLLGELAYKLGEYRIAIRAYRIALKRGNPNANPNANPNANPNAEAWYNLGNAYYKQGRYREAIEYYQKALELDPNNAEAWYNLGNAYYERGEYEEAIEYYRKALRLDPNNADAMQNLLNAKMREELEHHHHHH
dn5bL1R5_03	I53-dn5	5 sets of NANP repeats in loop 1 and on N-term of I53-dn5B	Yes		No	N/A	MNANPNPDPNANPNVDPNANPGGSGGDGGSGGDEEAELAYLLGELAYKLGEYRIAIRAYRIALKRGNPNANPNANPNANPNANPNAEAWYNLGNAYYKQGRYREAIEYYQKALELDPNNAEAWYNLGNAYYERGEYEEAIEYYRKALRLDPNNADAMQNLLNAKMREELEHHHHHH
dn5bL2R3_04	I53-dn5	3 sets of NANP repeats in loop 2 and on N-term of I53-dn5B	Yes		No	N/A	MNANPNPDPNANPNVDPNANPGGSGGDGGSGGDEEAELAYLLGELAYKLGEYRIAIRAYRIALKRGNPNANPNANPNANPNAEAWYNLGNAYYKQGRYREAIEYYQKALELDPNNAEAWYNLGNAYYERGEYEEAIEYYRKALRLDPNNADAMQNLLNAKMREELEHHHHHH
dn5bL2R4_05	I53-dn5	4 sets of NANP repeats in loop 2 and on N-term of I53-dn5B	Yes		No	N/A	MNANPNPDPNANPNVDPNANPGGSGGDGGSGGDEEAELAYLLGELAYKLGEYRIAIRAYRIALKRDPNNAEAWYNLGNAYYKQGRYREAIEYYQKALELGNPNANPNANPNANPNAEAWYNLGNAYYERGEYEEAIEYYRKALRLDPNNADAMQNLLNAKMREELEHHHHHH
dn5bL2R5_06	I53-dn5	5 sets of NANP repeats in loop 2 and on N-term of I53-dn5B	Yes		No	N/A	MNANPNPDPNANPNVDPNANPGGSGGDGGSGGDEEAELAYLLGELAYKLGEYRIAIRAYRIALKRDPNNAEAWYNLGNAYYKQGRYREAIEYYQKALELGNPNANPNANPNANPNAEAWYNLGNAYYERGEYEEAIEYYRKALRLDPNNADAMQNLLNAKMREELEHHHHHH
dn5bL12R3_07	I53-dn5	3 sets of NANP repeats in loops 1,2 and on N-term of I53-dn5B	Yes		No	N/A	MNANPNPDPNANPNVDPNANPGGSGGDGGSGGDEEAELAYLLGELAYKLGEYRIAIRAYRIALKRGNPNANPNANPNAEAWYNLGNAYYKQGRYREAIEYYQKALELGNPNANPNANPNAEAWYNLGNAYYERGEYEEAIEYYRKALRLDPN

							NADAMQNLLNAKMREELEHHHHHH
dn5bL12R4_08	I53-dn5	4 sets of NANP repeats in loops 1,2 and on N-term of I53-dn5B	Yes		No	N/A	MNANPNPDPNANPNVDPNANPGGSGGDGGSGGDEEAELAYLLGELAYKLGEYRIAIRAYRIALKRGNPNANPNANPNANPNAEAWYNLGNAYYKQGRYREAIEYYQKALELGNPNANPNANPNAEAWYNLGNAYYERGEYEEAIEYYRKALRLDPNNADAMQNLLNAKMREELEHHHHHH
dn5bL12R5_09	I53-dn5	5 sets of NANP repeats in loops 1,2 and on N-term of I53-dn5B	Yes		No	N/A	MNANPNPDPNANPNVDPNANPGGSGGDGGSGGDEEAELAYLLGELAYKLGEYRIAIRAYRIALKRGNPNANPNANPNANPNANPNAEAWYNLGNAYYKQGRYREAIEYYQKALELGNPNANPNANPNAEAWYNLGNAYYERGEYEEAIEYYRKALRLDPNNADAMQNLLNAKMREELEHHHHHH
dn5bL1_junctional_10	I53-dn5	Junctional region in loop 1 and on N-term of I53-dn5B	Yes		No	N/A	MNANPNPDPNANPNVDPNANPGGSGGDGGSGGDEEAELAYLLGELAYKLGEYRIAIRAYRIALKRGNANPNPDPNANPNVDPNANPNAEAWYNLGNAYYKQGRYREAIEYYQKALELDPNNAEAWYNLGNAYYERGEYEEAIEYYRKALRLDPNNADAMQNLLNAKMREELEHHHHHH
dn5bL2_junctional_11	I53-dn5	Junctional region in loop 2 and on N-term of I53-dn5B	Yes		No	N/A	MNANPNPDPNANPNVDPNANPGGSGGDGGSGGDEEAELAYLLGELAYKLGEYRIAIRAYRIALKRDPNNAEAWYNLGNAYYKQGRYREAIEYYQKALELGNANPNPDPNANPNVDPNANPNAEAWYNLGNAYYERGEYEEAIEYYRKALRLDPNNADAMQNLLNAKMREELEHHHHHH
dn5bL12_junctional_12	I53-dn5	Junctional region in loops 1,2 and on N-term of I53-dn5B	Yes		No	N/A	MNANPNPDPNANPNVDPNANPGGSGGDGGSGGDEEAELAYLLGELAYKLGEYRIAIRAYRIALKRGNANPNPDPNANPNVDPNANPNAEAWYNLGNAYYKQGRYREAIEYYQKALELGNANPNPDPNANPNVDPNANPNAEAWYNLGNAYYERGEYEEAIEYYRKALRLDPNNADAMQNLLNAKMREELEHHHHHH
dn5bL_013	I53-dn5	peptide 21 in loop 1,2 without an N terminal fusion I53-dn5B	Yes		No	N/A	MEEAELAYLLGELAYKLGEYRIAIRAYRIALKRGNANPNPDPNANPNVDPNANPNAEAWYNLGNAYYKQGRYREAIEYYQKALELGNANPNPDPNANPNVDPNANPNAEAWYNLGNAYYERGEYEEAIEYYRKALRLDPNNADAMQNLLNAKMREELEHHHHHH
dn5bL_014	I53-dn5	peptide 21 in loop 2 without an N terminal fusion I53-dn5B	Yes		No	N/A	MEEAELAYLLGELAYKLGEYRIAIRAYRIALKRDPNNAEAWYNLGNAYYKQGRYREAIEYYQKALELGNANPNPDPNANPNVDPNANPNAEAWYNLGNAYYERGEYEEAIEYYRKALRLDPNNADAMQNLLNAKMREELEHHHHHH
dn5bL_015	I53-dn5	peptide 21 in loop 1 without an N terminal fusion I53-dn5B	Yes		No	N/A	MEEAELAYLLGELAYKLGEYRIAIRAYRIALKRGNANPNPDPNANPNVDPNANPNAEAWYNLGNAYYKQGRYREAIEYYQKALELDPNNAEAWYNLGNAYYERGEYEEAIEYYRKALRLDPNNADAMQNLLNAKMREELEHHHHHH
dn5bL_016	I53-dn5	NPDP in each loop on I53-dn5B	Yes		No	N/A	MEEAELAYLLGELAYKLGEYRIAIRAYRIALKRGNPDPNAEAWYNLGNAYYKQGRYREAIEYYQKALELGNPDPNAEAWYNLGNAYYER

							GEYEEAIEYYRKALRLDPNNAD<br>AMQNLLNAKMREELEHHHHHH
dn5bL_017	I53-dn5	NPNV in each loop on I53-dn5B	Yes		No	N/A	MEEAELAYLLGELAYKLGEYRIAI<br>RAYRIALKRGNPNVNAEAWYNL<br>GNAYYKQGRYREAIEYYQKALE<br>LGNPNVNAEAWYNLGNAYYER<br>GEYEEAIEYYRKALRLDPNNAD<br>AMQNLLNAKMREELEHHHHHH
dn5bL_018	I53-dn5	NPNA in each loop on I53-dn5B	Yes		No	N/A	MEEAELAYLLGELAYKLGEYRIAI<br>RAYRIALKRGNPNANAEAWYNL<br>GNAYYKQGRYREAIEYYQKALE<br>LGNPNANAEAWYNLGNAYYER<br>GEYEEAIEYYRKALRLDPNNAD<br>AMQNLLNAKMREELEHHHHHH
T33_dn10B_Major_loop1234_<br>01	T33-dn10	NPNA in all loops on T33-dn10B	Yes	N/A	No	N/A	MIEEVVAEMIDILAESSKKSIEEL<br>ARAADNKTTEKAVAEAIEEIARL<br>ATAAIQLIEALAKNLASNGGNPN<br>ANPNANPNANPNANPNAGGEE<br>FMARAISAIAELAKKAIEAIYRLA<br>DNHTTDTFMARAIAAIANLAVTAI<br>LAIAALASNGGNPNANPNANPN<br>ANPNANPNAGGEEFMARAISAI<br>AELAKKAIEAIYRLADNHTTDKF<br>MAAAIEAIALLATLAILAIALLASN<br>GGNPNANPNANPNANPNANPN<br>AGGEKFMARAIMAIAILAAKAIEA<br>IYRLADNHTSPTYIEKAIEAIEKIA<br>RKAIKAIEMLAKNGGNPNANPN<br>ANPNANPNANPNAGGEEYKEK<br>AKKIIDIIRKLAKMAIKKLEDNRTL<br>EHHHHHH
T33_dn10B_Major_loop1234_<br>PADRE_02	T33-dn10	NPNA in all loops + PADRE peptide<br>on T33-dn10B	Yes	N/A	No	N/A	MIEEVVAEMIDILAESSKKSIEEL<br>ARAADNKTTEKAVAEAIEEIARL<br>ATAAIQLIEALAKNLASNGGNPN<br>ANPNANPNANPNANPNAGGEE<br>FMARAISAIAELAKKAIEAIYRLA<br>DNHTTDTFMARAIAAIANLAVTAI<br>LAIAALASNGGNPNANPNANPN<br>ANPNANPNAGGEEFMARAISAI<br>AELAKKAIEAIYRLADNHTTDKF<br>MAAAIEAIALLATLAILAIALLASN<br>GGNPNANPNANPNANPNANPN<br>AGGEKFMARAIMAIAILAAKAIEA<br>IYRLADNHTSPTYIEKAIEAIEKIA<br>RKAIKAIEMLAKNGGNPNANPN<br>ANPNANPNANPNAGGEEYKEK<br>AKKIIDIIRKLAKMAIKKLEDNRTL<br>EDESDESAKFVAAWTLKAAADE<br>SDESHHHHHH
T33_dn10B_Junctional_loop1<br>234_03	T33-dn10	NPDP in all loops on T33-dn10B	Yes	N/A	No	N/A	MIEEVVAEMIDILAESSKKSIEEL<br>ARAADNKTTEKAVAEAIEEIARL<br>ATAAIQLIEALAKNLASNGGNPN<br>ANPNPDPNANPNVDPNAGGEE<br>FMARAISAIAELAKKAIEAIYRLA<br>DNHTTDTFMARAIAAIANLAVTAI<br>LAIAALASNGGNPNANPNPDPN<br>ANPNVDPNAGGEEFMARAISAI<br>AELAKKAIEAIYRLADNHTTDKF<br>MAAAIEAIALLATLAILAIALLASN<br>GGNPNANPNPDPNANPNVDPN<br>AGGEKFMARAIMAIAILAAKAIEA<br>IYRLADNHTSPTYIEKAIEAIEKIA<br>RKAIKAIEMLAKNGGNPNANPN<br>PDPNANPNVDPNAGGEEYKEK<br>AKKIIDIIRKLAKMAIKKLEDNRTL<br>EHHHHHH
T33_dn10B_Junctional_loop1<br>234_PADRE_04	T33-dn10	NPDP in all loops + PADRE peptide<br>on T33-dn10B	Yes	N/A	No	N/A	MIEEVVAEMIDILAESSKKSIEEL<br>ARAADNKTTEKAVAEAIEEIARL<br>ATAAIQLIEALAKNLASNGGNPN<br>ANPNPDPNANPNVDPNAGGEE<br>FMARAISAIAELAKKAIEAIYRLA<br>DNHTTDTFMARAIAAIANLAVTAI<br>LAIAALASNGGNPNANPNPDPN<br>ANPNVDPNAGGEEFMARAISAI<br>AELAKKAIEAIYRLADNHTTDKF<br>MAAAIEAIALLATLAILAIALLASN

							GGNPNANPNPDPNANPNVDPN<br>AGGEKFMARAIMAIAILAAKAIEA<br>IYRLADNHTSPTYIEKAIEAIEKIA<br>RKAIKAIEMLAKNGGNPNANPN<br>PDPNANPNVDPNAGGEEYKEK<br>AKKIIDIIRKLAKMAIKKLEDNRTL<br>EDESDESAKFVAAWTLKAAADE<br>SDESHHHHHH
T33_dn10B_Minor_loop1234_<br>05	T33-dn10	NPNV in all loops on T33-dn10B	Yes	N/A	No	N/A	MIEEVVAEMIDILAESSKKSIEEL<br>ARAADNKTTEKAVAEAIEEIARL<br>ATAAIQLIEALAKNLASNGGNPN<br>ANPNVDPNANPNVDPNAGGEE<br>FMARAISAIAELAKKAIEAIYRLA<br>DNHTTDTFMARAIAAIANLAVTAI<br>LAIAALASNGGNPNANPNVDPN<br>ANPNVDPNAGGEEFMARAISAI<br>AELAKKAIEAIYRLADNHTTDKF<br>MAAAIEAIALLATLAILAIALLASN<br>GGNPNANPNVDPNANPNVDPN<br>AGGEKFMARAIMAIAILAAKAIEA<br>IYRLADNHTSPTYIEKAIEAIEKIA<br>RKAIKAIEMLAKNGGNPNANPN<br>VDPNANPNVDPNAGGEEYKEK<br>AKKIIDIIRKLAKMAIKKLEDNRTL<br>EHHHHHH
T33_dn10B_Minor_loop1234_<br>PADRE_06	T33-dn10	NPNV in all loops + PADRE peptide on T33-dn10B	Yes	N/A	No	N/A	MIEEVVAEMIDILAESSKKSIEEL<br>ARAADNKTTEKAVAEAIEEIARL<br>ATAAIQLIEALAKNLASNGGNPN<br>ANPNVDPNANPNVDPNAGGEE<br>FMARAISAIAELAKKAIEAIYRLA<br>DNHTTDTFMARAIAAIANLAVTAI<br>LAIAALASNGGNPNANPNVDPN<br>ANPNVDPNAGGEEFMARAISAI<br>AELAKKAIEAIYRLADNHTTDKF<br>MAAAIEAIALLATLAILAIALLASN<br>GGNPNANPNVDPNANPNVDPN<br>AGGEKFMARAIMAIAILAAKAIEA<br>IYRLADNHTSPTYIEKAIEAIEKIA<br>RKAIKAIEMLAKNGGNPNANPN<br>VDPNANPNVDPNAGGEEYKEK<br>AKKIIDIIRKLAKMAIKKLEDNRTL<br>EDESDESAKFVAAWTLKAAADE<br>SDESHHHHHH
T33_dn10B_Junctional_loop2<br>34_07	T33-dn10	NPDP in loops 2,3,4 on T33-dn10B	Yes	N/A	No	N/A	MIEEVVAEMIDILAESSKKSIEEL<br>ARAADNKTTEKAVAEAIEEIARL<br>ATAAIQLIEALAKNLASEEFMAR<br>AISAIAELAKKAIEAIYRLADNHT<br>TDTFMARAIAAIANLAVTAILAIAA<br>LASNGGNPNANPNPDPNANPN<br>VDPNAGGEEFMARAISAIAELAK<br>KAIEAIYRLADNHTTDKFMAAAI<br>EAIALLATLAILAIALLASNGGNP<br>NANPNPDPNANPNVDPNAGGE<br>KFMARAIMAIAILAAKAIEAIYRLA<br>DNHTSPTYIEKAIEAIEKIARKAIK<br>AIEMLAKNGGNPNANPNPDPNA<br>NPNVDPNAGGEEYKEKAKKIIDII<br>RKLAKMAIKKLEDNRTLEHHHH<br>HH
T33_dn10B_Junctional_loop2<br>34_PADRE_08	T33-dn10	NPDP in loops 2,3,4 + PADRE peptide on T33-dn10B	Yes	N/A	No	N/A	MIEEVVAEMIDILAESSKKSIEEL<br>ARAADNKTTEKAVAEAIEEIARL<br>ATAAIQLIEALAKNLASEEFMAR<br>AISAIAELAKKAIEAIYRLADNHT<br>TDTFMARAIAAIANLAVTAILAIAA<br>LASNGGNPNANPNPDPNANPN<br>VDPNAGGEEFMARAISAIAELAK<br>KAIEAIYRLADNHTTDKFMAAAI<br>EAIALLATLAILAIALLASNGGNP<br>NANPNPDPNANPNVDPNAGGE<br>KFMARAIMAIAILAAKAIEAIYRLA<br>DNHTSPTYIEKAIEAIEKIARKAIK<br>AIEMLAKNGGNPNANPNPDPNA<br>NPNVDPNAGGEEYKEKAKKIIDII<br>RKLAKMAIKKLEDNRTLEDESD<br>ESAKFVAAWTLKAAADESDESH<br>HHHHH

T33_dn10B_Minor_loop234_09	T33-dn10	NPNV in loops 2,3,4 on T33-dn10B	Yes	N/A	No	N/A	MIEEVVAEMIDILAESSKKSIEELARAADNKTTEKAVAEAIEEIARLATAAIQLIEALAKNLASEEFMARAISAIAELAKKAIEAIYRLADNHTTDTFMARAIAAIANLAVTAILAIAALASNGGNPNANPNVDPNANPNVDPNAGGEEFMARAISAIAELAKKAIEAIYRLADNHTTDKFMAAAIEAIALLATLAILAIALLASNGGNPNANPNVDPNANPNVDPNAGGEKFMARAIMAIAILAAKAIEAIYRLADNHTSPTYIEKAIEAIEKIARKAIKAIEMLAKNGGNPNANPNVDPNANPNVDPNAGGEEYKEKAKKIIDIIRKLAKMAIKKLEDNRTLEHHHHHH
T33_dn10B_Minor_loop234_PADRE_10	T33-dn10	NPNV in loops 2,3,4 + PADRE peptide on T33-dn10B	Yes	N/A	No	N/A	MIEEVVAEMIDILAESSKKSIEELARAADNKTTEKAVAEAIEEIARLATAAIQLIEALAKNLASNEEFMARAISAIAELAKKAIEAIYRLADNHTTDTFMARAIAAIANLAVTAILAIAALASNGGNPNANPNVDPNANPNVDPNAGGEEFMARAISAIAELAKKAIEAIYRLADNHTTDKFMAAAIEAIALLATLAILAIALLASNGGNPNANPNVDPNANPNVDPNAGGEKFMARAIMAIAILAAKAIEAIYRLADNHTSPTYIEKAIEAIEKIARKAIKAIEMLAKNGGNPNANPNVDPNANPNVDPNAGGEEYKEKAKKIIDIIRKLAKMAIKKLEDNRTLEDESDESAKFVAAWTLKAAADESDESHHHHHH
T33_dn10B_Major_loop234_11	T33-dn10	NPNA in loops 2,3,4 on T33-dn10B	Yes	N/A	No	N/A	MIEEVVAEMIDILAESSKKSIEELARAADNKTTEKAVAEAIEEIARLATAAIQLIEALAKNLASNEEFMARAISAIAELAKKAIEAIYRLADNHTTDTFMARAIAAIANLAVTAILAIAALASNGGNPNANPNANPNANPNANPNAGGEEFMARAISAIAELAKKAIEAIYRLADNHTTDKFMAAAIEAIALLATLAILAIALLASNGGNPNANPNANPNANPNANPNAGGEKFMARAIMAIAILAAKAIEAIYRLADNHTSPTYIEKAIEAIEKIARKAIKAIEMLAKNGGNPNANPNANPNANPNAGGEEYKEKAKKIIDIIRKLAKMAIKKLEDNRTLEHHHHHH
T33_dn10B_Major_loop234_PADRE_12	T33-dn10	NPNA in loops 2,3,4 + PADRE peptide on T33-dn10B	Yes	N/A	No	N/A	MIEEVVAEMIDILAESSKKSIEELARAADNKTTEKAVAEAIEEIARLATAAIQLIEALAKNLASNEEFMARAISAIAELAKKAIEAIYRLADNHTTDTFMARAIAAIANLAVTAILAIAALASNGGNPNANPNANPNANPNANPNAGGEEFMARAISAIAELAKKAIEAIYRLADNHTTDKFMAAAIEAIALLATLAILAIALLASNGGNPNANPNANPNANPNANPNAGGEKFMARAIMAIAILAAKAIEAIYRLADNHTSPTYIEKAIEAIEKIARKAIKAIEMLAKNGGNPNANPNANPNANPNAGGEEYKEKAKKIIDIIRKLAKMAIKKLEDNRTLEDESDESAKFVAAWTLKAAADESDESHHHHHH
T33_dn10B_Major_loop24_13	T33-dn10	NPNA in loops 2,4 on T33-dn10B	Yes	N/A	No	N/A	MIEEVVAEMIDILAESSKKSIEELARAADNKTTEKAVAEAIEEIARLATAAIQLIEALAKNLASNEEFMARAISAIAELAKKAIEAIYRLADNHTTDTFMARAIAAIANLAVTAILAIAALASNGGNPNANPNANPNANPNAGGEEFMARAISAIAELAKKAIEAIYRLADNHTTDKFMAAAIEAIALLATLAILAIALLASNHTTEKFMARAIMAIAILAAKAIEAIYRLADNHTSPTYIEKAIEAIEKIARKAIKAIEMLAKNGGNPNANPNANPNA

							NPNANPNAGGEEYKEKAKKIIDIIRKLAKMAIKKLEDNRTLEHHHHHH
T33_dn10B_Major_loop24_PADRE_14	T33-dn10	NPNA in loops 2,4 + PADRE peptide on T33-dn10B	Yes	N/A	No	N/A	MIEEVVAEMIDILAESSKKSIEELARAADNKTTEKAVAEAIEEIARLATAAIQLIEALAKNLASNEEFMARAISAIAELAKKAIEAIYRLADNHTTDTFMARAIAAIANLAVTAILAIAALASNGGNPNANPNANPNANPNANPNAGGEEFMARAISAIAELAKKAIEAIYRLADNHTTDKFMAAAIEAIALLATLAILAIALLASNHTTEKFMARAIMAIAILAAKAIEAIYRLADNHTSPTYIEKAIEAIEKIARKAIKAIEMLAKNGGNPNANPNANPNANPNANPNAGGEEYKEKAKKIIDIIRKLAKMAIKKLEDNRTLEDESDESAKFVAAWTLKAAADESDESHHHHHH
T33_dn10B_Minor_loop24_15	T33-dn10	NPNV in loops 2,4 on T33-dn10B	Yes	N/A	No	N/A	MIEEVVAEMIDILAESSKKSIEELARAADNKTTEKAVAEAIEEIARLATAAIQLIEALAKNLASEEFMARAISAIAELAKKAIEAIYRLADNHTTDTFMARAIAAIANLAVTAILAIAALASNGGNPNANPNVDPNANPNVDPNAGGEEFMARAISAIAELAKKAIEAIYRLADNHTTDKFMAAAIEAIALLATLAILAIALLASNHTTEKFMARAIMAIAILAAKAIEAIYRLADNHTSPTYIEKAIEAIEKIARKAIKAIEMLAKNGGNPNANPNVDPNANPNVDPNAGGEEYKEKAKKIIDIIRKLAKMAIKKLEDNRTLEHHHHHH
T33_dn10B_Minor_loop24_PADRE_16	T33-dn10	NPNV in loops 2,4 + PADRE peptide on T33-dn10B	Yes	N/A	No	N/A	MIEEVVAEMIDILAESSKKSIEELARAADNKTTEKAVAEAIEEIARLATAAIQLIEALAKNLASNEEFMARAISAIAELAKKAIEAIYRLADNHTTDTFMARAIAAIANLAVTAILAIAALASNGGNPNANPNVDPNANPNVDPNAGGEEFMARAISAIAELAKKAIEAIYRLADNHTTDKFMAAAIEAIALLATLAILAIALLASNHTTEKFMARAIMAIAILAAKAIEAIYRLADNHTSPTYIEKAIEAIEKIARKAIKAIEMLAKNGGNPNANPNVDPNANPNVDPNAGGEEYKEKAKKIIDIIRKLAKMAIKKLEDNRTLEDESDESAKFVAAWTLKAAADESDESHHHHHH
T33_dn10B_Junctional_loop24_17	T33-dn10	NPDP in loops 2,4 on T33-dn10B	Yes	N/A	No	N/A	MIEEVVAEMIDILAESSKKSIEELARAADNKTTEKAVAEAIEEIARLATAAIQLIEALAKNLASEEFMARAISAIAELAKKAIEAIYRLADNHTTDTFMARAIAAIANLAVTAILAIAALASNGGNPNANPNPDPNANPNVDPNAGGEEFMARAISAIAELAKKAIEAIYRLADNHTTDKFMAAAIEAIALLATLAILAIALLASNHTTEKFMARAIMAIAILAAKAIEAIYRLADNHTSPTYIEKAIEAIEKIARKAIKAIEMLAKNGGNPNANPNPDPNANPNVDPNAGGEEYKEKAKKIIDIIRKLAKMAIKKLEDNRTLEHHHHHH
T33_dn10B_Junctional_loop24_PADRE_18	T33-dn10	NPDP in loops 2,4 + PADRE peptide on T33-dn10B	Yes	N/A	No	N/A	MIEEVVAEMIDILAESSKKSIEELARAADNKTTEKAVAEAIEEIARLATAAIQLIEALAKNLASEEFMARAISAIAELAKKAIEAIYRLADNHTTDTFMARAIAAIANLAVTAILAIAALASNGGNPNANPNPDPNANPNVDPNAGGEEFMARAISAIAELAKKAIEAIYRLADNHTTDKFMAAAIEAIALLATLAILAIALLASNHTTEKFMARAIMAIAILAAKAIEAIYRLADNHTSPTYIEKAIEAIEKIARKAIKAIEMLAKNGGNPNANPNPDPNA

							NPNVDPNAGGEEYKEKAKKIIDII<br>RKLAKMAIKKLEDNRTLEDESD<br>ESAKFVAAWTLKAAADESDESH<br>HHHHH
I52.6_Junctional_Loop1234_01	I52.6	NPDP in all loops on i52.6	Yes	N/A	No	N/A	MSDEEERNELIKRIREAAQRAR<br>EAAERTGDPRVRELARELARLA<br>QRAFYLVLHDPSSSDVNEALKLI<br>VEAIEAAVRALEAAERTGDPKV<br>REEARELVRRAVEAAEEVQRNP<br>SSSEVNEKLKAIVVEIEVKVASLE<br>AKEVGGNPNANPNPDPNANPN<br>VDPNAGGPDKALKIAKKVIELAL<br>EAVKENPSTEALRAVLEAVRLAS<br>EVAKRVGGNPNANPNPDPNAN<br>PNVDPNAGGPDKALKIAKLVIEL<br>ALEAVKEDPSTDALRAVLEAVRL<br>ASEVAKRVGGNPNANPNPDPN<br>ANPNVDPNAGGPDKALKIAKLV<br>LELAAEAVKEDPSTDALRAAKE<br>AERLATEVAKRVGGNPNANPNP<br>DPNANPNVDPNAGGPKKAREIE<br>MLVLKLQMEAILAETEEVKKEIE<br>ESKKRPQSESAKNLILIMQLLIN<br>QIRLLALQIRMLALQLQEGSLEW<br>GGHHHHHH
I52.6_Major_Loop1234_02	I52.6	NPNA in all loops on i52.6	Yes	N/A	No	N/A	MSDEEERNELIKRIREAAQRAR<br>EAAERTGDPRVRELARELARLA<br>QRAFYLVLHDPSSSDVNEALKLI<br>VEAIEAAVRALEAAERTGDPKV<br>REEARELVRRAVEAAEEVQRNP<br>SSSEVNEKLKAIVVEIEVKVASLE<br>AKEVGGNPNANPNANPNANPN<br>ANPNAGGPDKALKIAKKVIELAL<br>EAVKENPSTEALRAVLEAVRLAS<br>EVAKRVGGNPNANPNANPNAN<br>PNANPNAGGPDKALKIAKLVIEL<br>ALEAVKEDPSTDALRAVLEAVRL<br>ASEVAKRVGGNPNANPNANPN<br>ANPNANPNAGGPDKALKIAKLV<br>LELAAEAVKEDPSTDALRAAK
I52.6_Minor_Loop1234_03	I52.6	NPNV in all loops on i52.6	Yes	N/A	No	N/A	MSDEEERNELIKRIREAAQRAR<br>EAAERTGDPRVRELARELARLA<br>QRAFYLVLHDPSSSDVNEALKLI<br>VEAIEAAVRALEAAERTGDPKV<br>REEARELVRRAVEAAEEVQRNP<br>SSSEVNEKLKAIVVEIEVKVASLE<br>AKEVGGNPNANPNVDPNANPN<br>VDPNAGGPDKALKIAKKVIELAL<br>EAVKENPSTEALRAVLEAVRLAS<br>EVAKRVGGNPNANPNVDPNAN<br>PNVDPNAGGPDKALKIAKLVIEL<br>ALEAVKEDPSTDALRAVLEAVRL<br>ASEVAKRVGGNPNANPNVDPN<br>ANPNVDPNAGGPDKALKIAKLV<br>LELAAEAVKEDPSTDALRAAKE<br>AERLATEVAKRVGGNPNANPNV<br>DPNANPNVDPNAGGPKKAREIE<br>MLVLKLQMEAILAETEEVKKEIE<br>ESKKRPQSESAKNLILIMQLLIN<br>QIRLLALQIRMLALQLQEGSLEW<br>GGHHHHHH
SAmut_CSP_5/3	N/A	PfCSP (3D7) with mutations C25S, K66S, K67S, R70A. Repeat region truncated to include only 5 major repeats and 3 minor repeats.	Yes	N/A	N/A	Not used	QEYQSYGSSSNTRVLNELNYD<br>NAGTNLYNELEMNYYGKQENW<br>YSLSSNSASLGENDDGNNEDN<br>EKLRKPKHKKLKQPADGNPDPN<br>ANPNVDPNANPNVDPNANPNV<br>DPNANPNANPNKNNQGNGQG<br>HNMPNDPNRNVDENANANSAV<br>KNNNNEEPSDKHIKEYLNKIQN<br>SLSTEWSPCSVTCGNGIQVRIK<br>PGSANKPKDELDYANDIEKKICK<br>MEKCSGSGLNDIFEAQKIEWHE<br>LEVLFQGPGHHHHHH

SAmut_CSP	N/A	PfCSP (3D7) with mutations C25S, K66S, K67S, R70A. 37694.68 kDa	Yes	N/A	N/A	Figure 5	QEYQSYGSSSNTRVLNELNYDNAGTNLYNELEMNYYGKQENWYSLSSNSASLGENDDGNNEDNEKLRKPKHKKLKQPADGNPDPNANPNVDPNANPNVDPNANPNVDPNANPNANPNANPNANPNANPNANPNANPNANPNANPNANPNANPNANPNANPNANPNANPNANPNANPNANPNVDPNANPNANPNANPNANPNANPNANPNANPNANPNANPNANPNANPNANPNANPNANPNANPNANPNANPNANPNANPNKNNQGNGQGHNMPNDPNRNVDENANANSAVKNNNNEEPSDKHIKEYLNKIQNSLSTEWSPCSVTCGNGIQVRIKPGSANKPKDELDYANDIEKKICKMEKCSGSGLNDIFEAQKIEWHELEVLFQGPGHHHHHH
SAmut-CSP-I53-50A	N/A	SAmut construct fused to I53-50A	Yes	N/A	Yes	Figure 5	MQEYQSYGSSSNTRVLNELNYDNAGTNLYNELEMNYYGKQENWYSLSSNSASLGENDDGNNEDNEKLRKPKHKKLKQPADGNPDPNANPNVDPNANPNVDPNANPNVDPNANPNANPNANPNANPNANPNANPNANPNANPNANPNANPNANPNANPNANPNANPNVDPNANPNANPNANPNANPNANPNANPNANPNANPNANPNANPNANPNANPNANPNANPNANPNANPNANPNANPNKNNQGNGQGHNMPNDPNRNVDENANANSAVKNNNNEEPSDKHIKEYLNKIQNSLSTEWSPCSVTCGNGIQVRIKPGSANKPKDELDYANDIEKKICKMEKCSKMEELFKKHKIVAVLRANSVEEAIEKAVAVFAGGVHLIEITFTVPDADTVIKALSVLKEKGAIIGAGTVTSVEQCRKAVESGAEFIVSPHLDEEISQFCKEKGVFYMPGVMTPTELVKAMKLGHDILKLFPGEVVGPQFVKAMKGPFPNVKFVPTGGVNLDNVCEWFKAGVLAVGVGDALVKGDPDEVREKAKKFVEKIRGCTELEHHHHHH
SAmut_CSP_5/3_SpyTag			Yes	N/A	Yes	Figure 1	QEYQSYGSSSNTRVLNELNYDNAGTNLYNELEMNYYGKQENWYSLSSNSASLGENDDGNNEDNEKLRKPKHKKLKQPADGNPDPNANPNVDPNANPNVDPNANPNVDPNANPNANPNKNNQGNGQGHNMPNDPNRNVDENANANSAVKNNNNEEPSDKHIKEYLNKIQNSLSTEWSPCSVTCGNGIQVRIKPGSANKPKDELDYANDIEKKICKMEKCSGSGLNDIFEAQKIEWHEFRGLISVNHHNVGLEVLFQGPGHHHHHH
SpyCatcher-C4b			Yes	N/A	Yes	Figure 1	MDAMKRGLCCVLLLCGAVFVSPSASMHHHHHHGAMVDTLSGLSSEQGQSGDMTIEEDSATHIKFSKRDEDGKELAGATMELRDSSGKTISTWISDGQVKDFYLYPGKYTFVETAAPDGYEVATAITFTVNEQGQVTVDGKATKGDAHIGGSGGSSKKQGDADVCGEVAYIQSVVSDCHVPTAELRTLLEIRKLFLEIQKLKVELQGLSKE
SpyCatcher-Ferritin			Yes	N/A	Yes	Figure 1	MDAMKRGLCCVLLLCGAVFVSPSASMHHHHHHGAMVDTLSGLSSEQGQSGDMTIEEDSATHIKFSKRDEDGKELAGATMELRDSSGKTISTWISDGQVKDFYLYPGKYTFVETAAPDGYEVATAITFTVNEQGQVTVNGKATKGDAHIGGSGGSGESQVRQQFSKDIEKLLNEQVNKEMQSSNLYMSMSSWCYTHS

							LDGAGLFLFDHAAEEYEHAKKLI<br>IFLNENNVPVQLTSISAPEHKFE<br>GLTQIFQKAYEHEQHISESINNIV<br>DHAIKSKDHATFNFLQWYVAEQ<br>HEEEVLFKDILDKIELIGNENHGL<br>YLADQYVKGIAKSRKSGSEIPRP<br>RPLANKGNL
SpyCatcher-I53/50			Yes	N/A	Yes	Figure 1	MHHHHHHSGAMVDTLSGLSSE<br>QGQSGDMTIEEDSATHIKFSKR<br>DEDGKELAGATMELRDSSGKTI<br>STWISDGQVKDFYLYPGKYTFV<br>ETAAPDGYEVATAITFTVNEQGQ<br>VTVNGKATKGDAHIGGSGGSG<br>GKMEELFKKHKIVAVLRANSVEE<br>AIEKAVAVFAGGVHLIEITFTVPD<br>ADTVIKALSVLKEKGAIIGAGTVT<br>SVEQCRKAVESGAEFIVSPHLD<br>EEISQFCKEKGVFYMPGVMTPT<br>ELVKAMKLGHDILKLFPGEVVGP<br>QFVKAMKGPFPNVKFVPTGGV<br>NLDNVCEWFKAGVLAVGVGDA<br>LVKGDPDEVREKAKKFVEKIRG<br>CTEGSLEHHHHHH
Peptide 20	Peptide	ELISA reagent	N/A	N/A	N/A	N/A	PADGNPDPNANPNVD
Peptide 21	Peptide	ELISA reagent	N/A	N/A	N/A	N/A	NPDPNANPNVDPNAN
Peptide 22	Peptide	ELISA reagent	N/A	N/A	N/A	N/A	NANPNVDPNANPNVD
Peptide 23	Peptide	ELISA reagent	N/A	N/A	N/A	N/A	NVDPNANPNVDPNAN
Peptide 27	Peptide	ELISA reagent	N/A	N/A	N/A	N/A	NVDPNANPNANPNAN
Peptide 29	Peptide	ELISA reagent	N/A	N/A	N/A	N/A	NANPNANPNANPNAN
Peptide 43	Peptide	ELISA reagent	N/A	N/A	N/A	N/A	NANPNANPNVDPNAN
Peptide 44	Peptide	ELISA reagent	N/A	N/A	N/A	N/A	NANPNVDPNANPNAN
Peptide 61	Peptide	ELISA reagent	N/A	N/A	N/A	N/A	NANPNANPNANPNKN
Peptide 20-23	Peptide	ELISA reagent	N/A	N/A	N/A	N/A	PADGNPDPNANPNVDPNANPN<br>VDPNAN
Repeat Peptide	Peptide	ELISA reagent	N/A	N/A	N/A	N/A	NANPNANPNANPNANPNANPN<br>ANPNANPNANPNANP

Supplementary Table 1. Table of all protein constructs and their sequences used in this study. Description of the scaffold, expression status, point of failure, nanoparticle production status, corresponding mouse study figure, and sequence are provided. The peptides listed were used for ELISA experiments.
